# Invisible Harms, Visible Benefits? Adverse Event Reporting in Physical Activity Interventions for Neurodevelopmental Disorders: A Meta-analysis and Critical Appraisal

**DOI:** 10.1186/s40798-026-01095-w

**Published:** 2026-09-02

**Authors:** Jinrong He, Changhui Peng, Lei Zhang, Dandan Wang, Xiaohuan Tan, Xili Wen, Xin Shen, Xueping Wu

**Affiliations:** 1 School of Physical Education, Shanghai University of Sport, Shanghai, 200438 China; 2 Department of Physical Education, University of Shanghai for Science and Technology, Shanghai, 200093 China

**Keywords:** Adverse events, Meta-analysis, Neurodevelopmental disorders, Physical activity

## Abstract

**Background:**

Clinical guidelines and practice increasingly endorse physical activity interventions for individuals with neurodevelopmental disorders. However, because monitoring and reporting of potential intervention-related harms are often incomplete, the current evidence base regarding safety remains limited.

**Methods:**

A systematic search of PubMed, Web of Science, Scopus, the Cochrane Library, and EBSCO was conducted on November 11, 2025, followed by three rounds of supplementary snowball searching completed on November 20, 2025. Eligible studies were randomized controlled trials of physical activity interventions in populations with neurodevelopmental disorders. Following prespecified procedures recommended by the CONSORT-Harms extension and the Cochrane Handbook for Systematic Reviews of Interventions, we characterized adverse event monitoring and reporting practices across studies, summarized reasons for participant withdrawal, and considered the potential safety signals these reasons might indicate. Random-effects meta-analyses estimated the relative risk of adverse events for physical activity interventions versus non-exercise controls, and potential moderators were examined via subgroup and meta-regression analyses.

**Results:**

Among the 41,852 deduplicated records, 309 randomized controlled trials were included, comprising 322 study arms and 13,228 participants with neurodevelopmental disorders. None of the included trials fully complied with CONSORT-Harms recommendations for systematic harm monitoring and reporting. Only 58 trials described adverse event monitoring, mostly using passive, non-prespecified procedures; among those reporting results, approximately one quarter reported at least one adverse event or adverse effect, most commonly intervention-induced delayed-onset muscle soreness, mild pain or irritation, and related psychological discomfort. Among the 201 studies that reported reasons for participant withdrawal, 38 statements were too vague to determine the event characteristics. Sixteen studies reported adverse events (e.g., health problems and emotional discomfort); however, because none used a systematic adjudication procedure, attributing these events to the intervention was difficult. Forty-five studies met the inclusion criteria for meta-analysis. The pooled analysis indicated that physical activity interventions did not significantly increase the risk of reported adverse events (RR = 1.14; 95% CI 0.67–1.94). However, trials with prespecified monitoring procedures had a significantly higher risk estimate (RR = 4.32, 95% CI 1.36–13.68).

**Conclusions:**

Existing reported results suggest that these interventions do not significantly increase the overall risk of adverse events. However, the strength of this conclusion is limited by widespread systematic underreporting in the original studies and by substantial heterogeneity in methods used to monitor, identify, and assess harms. In contrast, studies that implemented prespecified adverse event monitoring procedures were generally able to identify and report adverse events more comprehensively, rather than indicating that these trials carry a genuinely higher risk. Future research should establish and implement standardized procedures for adverse event monitoring and reporting, standardize procedures for adjudicating and attributing reasons for withdrawal, and systematically report key variables related to intervention implementation and descriptive adverse event data to strengthen the credibility of benefit–risk assessments of physical activity interventions in this population.

**Supplementary Information:**

The online version contains supplementary material available at https://doi.org/10.1186/s40798-026-01095-w.

## Key points

1. Current evidence suggests that physical activity interventions for individuals with neurodevelopmental disorders do not significantly increase the overall risk of reported adverse events, although this conclusion is constrained by pervasive underreporting and inconsistent harm surveillance across trials.
2. Trials that implemented prespecified adverse event monitoring procedures reported substantially higher risk estimates, indicating that structured surveillance likely improves harm detection rather than reflecting a genuinely greater intervention-related risk.
3. Future randomized controlled trials should adopt standardized, proactive frameworks for adverse event monitoring, withdrawal adjudication, and intervention reporting to enable more credible and clinically meaningful benefit–risk evaluations in neurodevelopmental populations.

**Supplementary Information:**

The online version contains supplementary material available at https://doi.org/10.1186/s40798-026-01095-w.

## Introduction

Neurodevelopmental disorders (NDDs) are a heterogeneous group of conditions that typically emerge in early development [1, 2]. They include attention-deficit/hyperactivity disorder, autism spectrum disorder, intellectual developmental disorder, as well as specific learning disorder, and communication and motor disorders [2]. These conditions are commonly characterized by persistent functional impairments and atypical patterns of information processing associated with neurodevelopmental differences, which may have lasting effects on development and adaptive functioning [1]. Estimates suggest that NDDs affect approximately 5% of the global population, and the associated burden places ongoing pressure on health care systems, educational services, and family caregivers, thereby posing a major challenge for rehabilitation medicine and public health [3, 4]. Although NDDs are heterogeneous in etiology, they share early-onset neurocognitive impairment and overlapping genetic and clinical features, which supports the view that these conditions can be conceptualized within a unified framework [2, 5–7].

In recent years, physical activity interventions have attracted increasing attention in evidence-based medicine and public health because they are cost-effective and have substantial potential for scalable implementation [8, 9]. Multiple authoritative clinical guidelines have incorporated physical activity interventions into practice frameworks for NDDs as a key component of comprehensive intervention strategies [10–13]. However, despite the rapidly growing evidence supporting the effectiveness of these interventions, adverse events among individuals with NDDs during physical activity interventions have not been sufficiently synthesized in previous systematic reviews and meta-analyses [9, 14, 15]. This gap limits the integration and application of safety-related evidence. In addition, CONSORT 2025 identifies harm-related information as a core reporting element for randomized trials [16]. All randomized controlled trials should systematically and rigorously collect and report descriptive data on relevant harms and implement a monitoring strategy that integrates prespecified procedures (i.e., a priori active surveillance plans) with non-prespecified procedures (i.e., participant self-reports), thereby providing a more reliable basis for risk assessment and supporting more cautious, evidence-informed intervention decisions [16, 17].

Based on this background, the present study examined how adverse events related to physical activity interventions have been reported in randomized controlled trials involving people with NDDs. Drawing on the CONSORT Harms Extension and the *Cochrane Handbook for Systematic Reviews of Interventions*, this study defines harms as any outcomes that occur during or after a physical activity intervention and negatively affect participants (e.g., sports-related injuries, emotional dysregulation, or functional decline) [17, 18]. Within this framework, “harm” encompasses two dimensions: adverse events and adverse effects. An adverse event is any unfavorable or harmful outcome temporally associated with a physical activity intervention but not necessarily caused by it. In contrast, an adverse effect is an adverse event for which there are reasonable grounds to suggest a causal relationship with the intervention [18].

This study focuses on the following three core questions:

1. Current status and characteristics of adverse events/adverse effects reporting: we quantified the proportion of included studies that reported adverse events or adverse effects and summarized their patterns and major clinical manifestations.
2. Withdrawal reporting and potential safety signals: we quantified the proportion of studies that reported participant withdrawal and the reasons provided, summarized the categories and content of the reported reasons, and identified adverse events or adverse effects that may have been implicitly reflected in these reasons.
3. Relative risk of adverse events and potential moderators: we estimated the relative risk of adverse events among individuals with NDDs who received physical activity interventions compared with non-exercising controls and examined whether diagnostic subtype and intervention type, intensity, dose, and delivery characteristics moderated this risk.

## Methods

This review was guided by the Preferred Reporting Items for Systematic Reviews and Meta-Analyses Statement (PRISMA) [19]. The review protocol was prospectively registered with the International Prospective Register of Systematic Reviews (PROSPERO) in November 2025 (ID: CRD420261279910). Compared with the registered protocol, SPORTDiscus was added to the database search to improve the comprehensiveness of the literature retrieval, given the review’s focus on physical activity interventions.

### Information Sources

Before initiating the formal systematic literature search, one author (J.H.) conducted a preliminary search of the PubMed database using the keywords “Neurodevelopmental Disorders” and “Physical Activity”, encompassing publications from 2000 through September 15, 2025, to identify contemporary evidence on physical activity interventions. During this preparatory phase, all retrieved records were individually screened, and studies deemed potentially relevant to physical activity-based interventions for individuals with NDDs were systematically archived in EndNote (version 20; Clarivate Analytics, Philadelphia, PA, USA). To identify high-frequency terminology and characterize prevailing research directions within this literature, the research team applied the Word Frequency Analyser available through the Systematic Review Accelerator platform (https://tera-tools.com/) to conduct keyword frequency analyses of the full texts of the initially included studies. Guided by these results and informed by established systematic reviews, we subsequently refined and operationalized a structured search strategy [15, 20]. The final search strategy was organized around two core concepts: NDDs and physical activity interventions (Supplementary Table 2).

### Search Strategy

On November 11, 2025, the research team conducted a systematic search of titles and abstracts across PubMed, Web of Science, Scopus, the Cochrane Library, and multiple sub-databases within the EBSCO platform, including SPORTDiscus, APA PsycInfo, APA PsycArticles, Psychology and Behavioral Sciences Collection, ERIC, and Medline (Supplementary Table 3). To increase the sensitivity and comprehensiveness of the search, three rounds of systematic snowballing strategies were also implemented: (a) reviewing the reference lists of the included studies; (b) identifying subsequent studies that cited the included articles; and (c) using the “Similar Articles” or “Find Similar” functions in PubMed and Embase to locate additional relevant literature. All formal search procedures were completed on November 20, 2025.

### Selection Process

All records retrieved from the searches were first imported into EndNote for automated deduplication and then manually verified, line by line, by an independent reviewer (J.H.), yielding 41,852 unique records. Subsequently, title and abstract screening was conducted via ASReview, an open-source active-learning system that iteratively reorders records based on reviewer feedback (see Supplementary Sect. 3 for an overview of ASReview and the screening strategy) [21–23]. Two reviewers (J.H. and C.P.) then independently screened titles and abstracts in ASReview, and any disagreements were resolved in consultation with a third reviewer (X.W.), who adjudicated the final inclusion decisions.

### Eligibility Criteria

A priori inclusion and exclusion criteria were applied to determine study eligibility according to the Population, Intervention, Comparison, Outcomes, and Study Design (PICOS) framework; details are provided in Supplementary Table 4. We included participants with a clearly established diagnosis of an NDD, with no restrictions on age, sex, geographic location, or the intervention delivery setting [1]. Eligible interventions focused on physical activity or included physical activity as a major component, with active exercise training accounting for at least 50% of the session time. To strengthen internal validity and minimize confounding when attributing adverse events, we excluded studies in which exercise was delivered concurrently with a multicomponent intervention that also included medication, surgery, or electrical stimulation/electrotherapy. We also excluded nonactive approaches in which voluntary muscular activity was minimal and the intervention relied primarily on passive modalities or static positioning/stretching (e.g., whole-body vibration, facial exercise, stretching or range-of-motion exercises only, and bladder training) [24]. For the meta-analysis, the control conditions included non-exercise treatment controls, usual care or wait-list, attention controls, health education interventions (e.g., “back school” and similar programs), placebo, and nutraceuticals. To avoid overlooking evidence relevant to adverse event reporting because of differences in comparators, the critical review imposed no restrictions on whether the control groups included an exercise component. We restricted the study design to randomized controlled trials (RCTs) that used a random allocation mechanism, including individually randomized parallel-group trials, cluster-randomized trials, randomized crossover trials, and stepped-wedge designs.

### Data Extraction

Two reviewers (J.H. and C.P.) independently extracted data using a customized Excel form (Microsoft Excel, version 16.93; Redmond, WA, USA) finalized before full-text screening began (see Supplementary Sect. 4 for detailed coding items). To identify and extract information on adverse events/adverse effects and participant withdrawals, both reviewers performed keyword searches of the full-text PDFs of the included studies using the following terms: “adverse event”, “adverse effect”, “side effect”, “complications”, “harm*”, “attrition”, “withdr*”, and “dropout”. If none of these keywords were identified in a study, the reviewers further examined the Methods section and relevant tables/figures to determine whether adverse events occurred and whether withdrawals and their reasons were reported. With respect to adverse event coding, studies were classified as either reporting adverse events (regardless of whether events occurred) or providing no relevant information. Adverse events were coded as adverse effects only under two conditions: (1) the original study authors explicitly stated that the event was related to, caused by, or attributable to the intervention or trial; or (2) according to the coding rules in the Cochrane Handbook, the event type could be clearly determined to be intervention-related [18]. On this basis, the 2 reviewers also recorded the number of withdrawals in each study and, using the same decision rules, classified the reasons for withdrawal as adverse events, adverse reactions, or unclear because the original reports provided insufficient information for attribution. Additionally, the reviewers independently extracted study characteristics (e.g., study design, setting/context, and post-randomization sample sizes by group), participant characteristics (e.g., diagnostic subtype, age, sex distribution, and country), and intervention characteristics (e.g., intervention type, interventionist type, intervention format, and intervention dose). A blinded coding auditor assessed data extraction reliability via Cohen’s kappa (κ) for categorical variables and the ICC(2,1) for continuous variables [25, 26]. Interrater agreement was substantial (κ = 0.85, categorical variables; ICC(2,1) = 0.89 for continuous variables). All discrepancies were resolved through discussion between the two reviewers without third-party arbitration.

### Risk of Bias

The risk of bias was assessed via the Cochrane-recommended risk of bias 2 tool (RoB 2) to systematically evaluate the methodological quality of the included studies across five domains: (1) bias arising from the randomization process; (2) bias due to deviations from intended interventions; (3) bias due to missing outcome data; (4) bias in outcome measurement; and (5) bias in the selection of the reported result, including potential bias in harm reporting [27]. For each domain, the risk of bias was judged as low, some concerns, or high. Disagreements were resolved through discussion between the reviewers (J.H. and C.P.); if consensus could not be reached, a third reviewer (X.W.) was consulted.

### Statistical Analysis

First, consistent with the variable type and prespecified analytic objectives, we calculated frequencies and percentages to describe adverse event reporting characteristics and the distribution of related outcomes. Second, we calculated the relative risks (RRs) of adverse events in R (version 4.5.2; R Core Team, Vienna, Austria) using the metafor package [28, 29]. Given the sparse distribution of adverse event data, we pooled effect sizes using a random-effects model with the Mantel–Haenszel (MH) method [30, 31]. For studies in which neither the physical activity group nor the control group reported any adverse events, we applied a treatment-arm continuity correction (TACC), allocating the correction proportionally to the between-group sample-size ratio to reduce bias due to unequal group sizes and avoid estimation bias and computational instability associated with zero-event studies [32, 33].

Interstudy heterogeneity was quantified via the *I*^2^ statistic. The interpretation of *I*^2^ followed prespecified thresholds: 0–25% indicated low heterogeneity, 25–50% indicated moderate heterogeneity, 50–75% indicated substantial heterogeneity, and  > 75% indicated considerable heterogeneity [34]. To examine potential moderators, we conducted subgroup analyses and meta-regression with statistical tests performed separately for categorical and continuous variables [35, 36]. In addition, we fitted meta-regression models with linear, quadratic, and cubic terms to evaluate the associations between the intervention dose and the relative risk (RR) of adverse events and to assess potential dose–response relationships [37, 38].

Publication bias was assessed using multiple complementary approaches. For visual assessment, we generated contour-enhanced funnel plots to identify patterns consistent with selective publication potentially related to statistical significance [39]. For statistical testing, we applied Egger’s linear regression test, Peters’ test, and Begg’s rank correlation test (Kendall’s τ), with *P* < 0.05 as the threshold for statistical significance and 0.05 ≤ *P* < 0.10 indicating marginal significance (suggesting a possible trend toward bias) [40–42]. To evaluate the robustness of the primary findings to analytic assumptions, we repeated the meta-analyses via the Mantel–Haenszel method with the Battaglia continuity correction, inverse-variance weighting with a restricted maximum likelihood (REML) estimator, and inverse-variance weighting with a DerSimonian–Laird estimator [31, 43, 44]. We then compared the effect estimates, confidence intervals, and statistical significance across these methodologically distinct strategies to assess whether the conclusions were consistent and not dependent on a single analytic approach.

### Certainty of the Evidence

The certainty of evidence for this meta-analysis was evaluated via the Grading of Recommendations, Assessment, Development, and Evaluation (GRADE) approach. GRADE evaluates evidence across five domains: study limitations, inconsistency, indirectness, imprecision, and other sources of bias [45]. Each domain is rated as not serious, serious, or very serious. After downgrading across domains, the overall certainty was classified as high, moderate, low, or very low. The ratings are summarized in an evidence profile table (Supplementary Table 6). The GRADE assessments were conducted independently by J.H. and verified by a second reviewer (X.W.) to ensure accuracy and consistency.

## Results

### Studies Retrieved

**Fig. 1:**
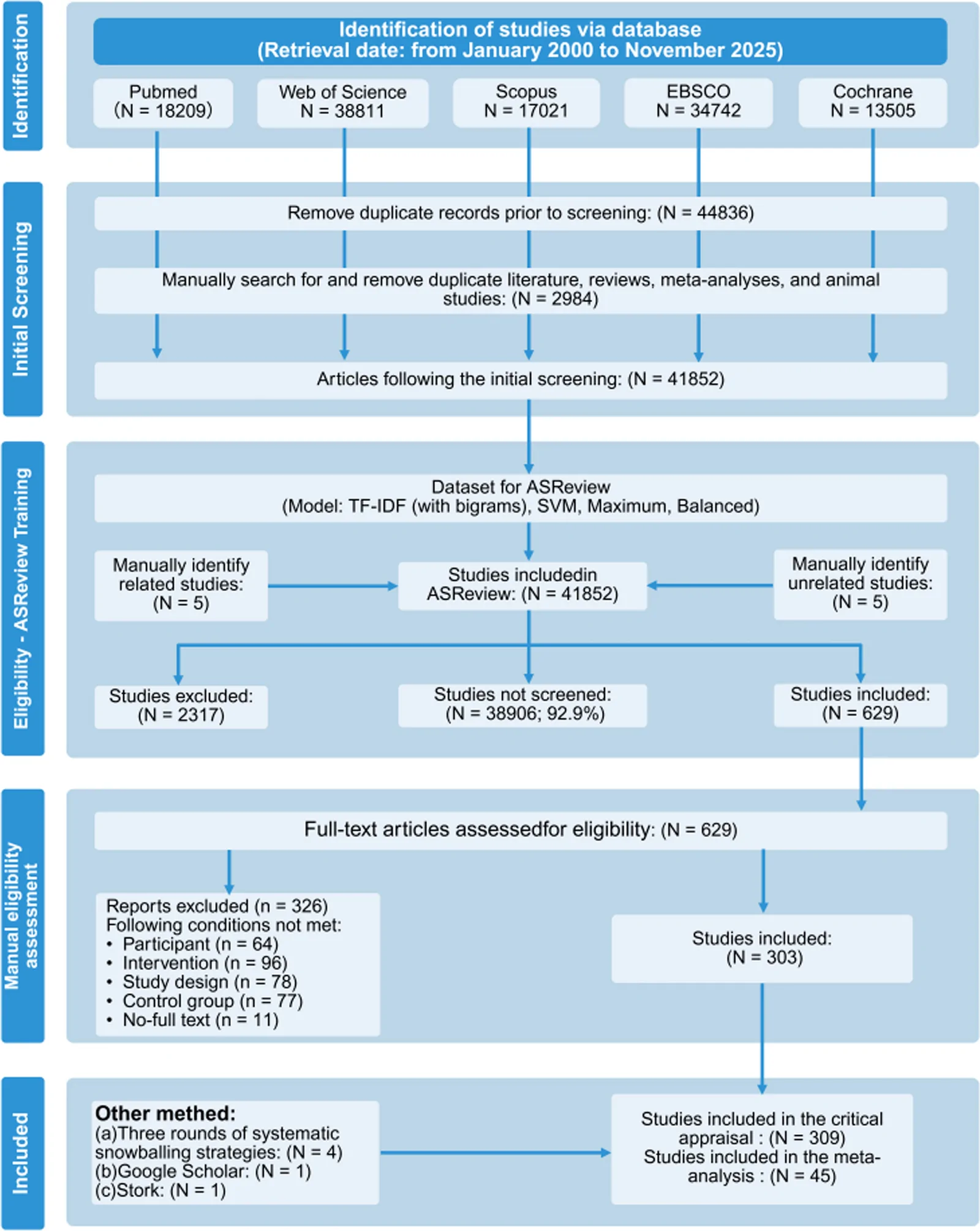
PRISMA flow diagram for included and excluded studies

In accordance with the PRISMA framework, 41,852 records remained after deduplication. After screening the titles and abstracts, 629 articles underwent full-text assessment, and 303 studies were deemed eligible (Fig. 1). These eligible studies were identified through backwards citation searching (*n* = 4) and supplementary searches via Google Scholar and Stork (*n* = 2). A total of 309 randomized controlled trials were included in this study. After excluding studies that did not report adverse event data and those in which the control group received a physical activity intervention, 45 trials were included in the meta-analysis.

### Study Characteristics and Global Landscape

**Fig. 2:**
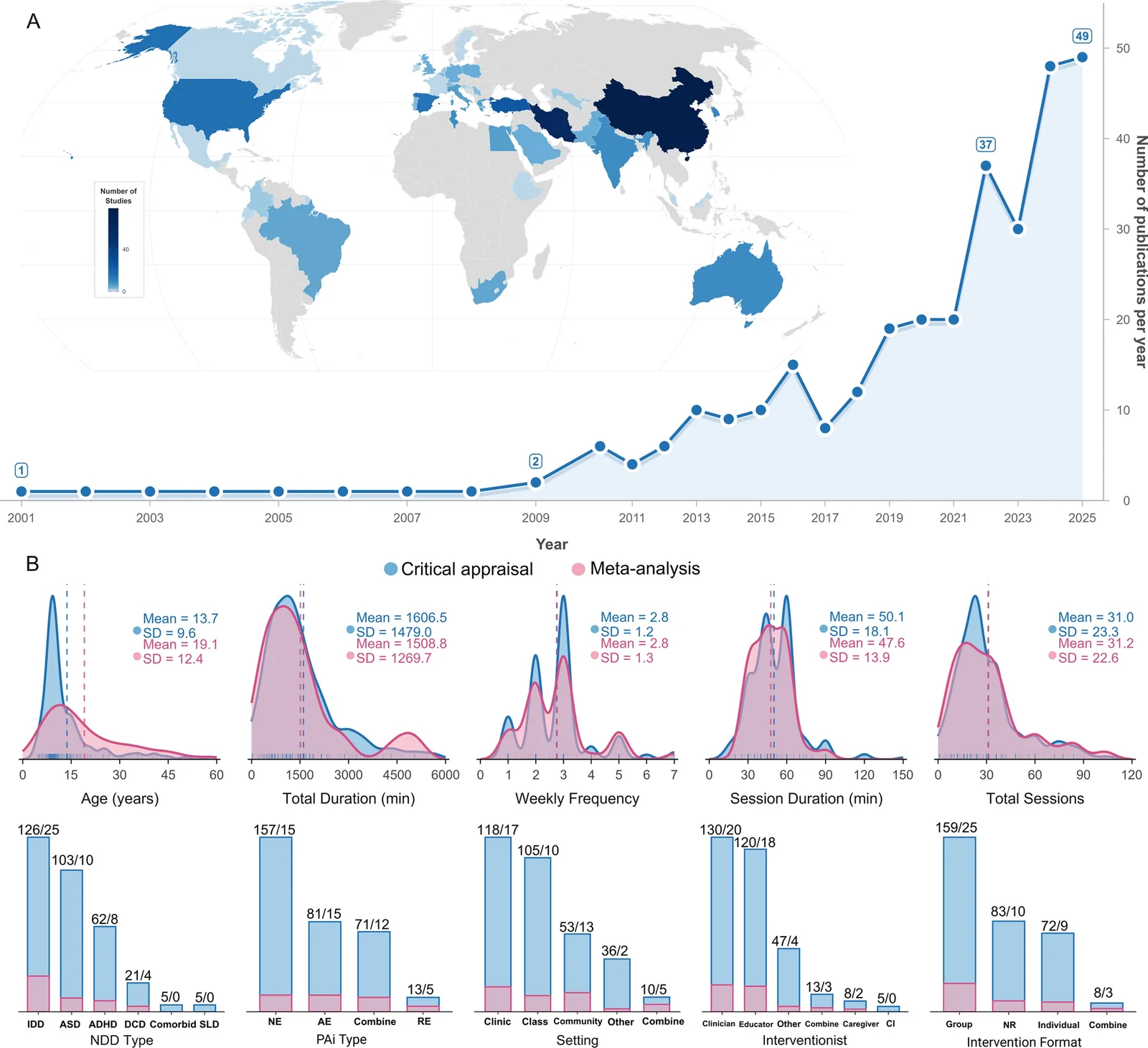
**A** Global trends in research on physical activity interventions for NDDs. **B** Overall frequency of intervention-related variables across studies. *ADHD* attention-deficit/hyperactivity disorder, *ASD* autism spectrum disorder, *AT* aerobic training, *CI* computer-based instruction, *DCD* developmental coordination disorder, *IDD* intellectual developmental disorder, *SLD* specific learning disorders, *NR* not reported, *NT* neuromuscular training, *PAi* physical activity intervention, *RT* resistance/strengthening training

Over the past 5 years, research on physical activity interventions for NDDs has markedly increased. The dataset synthesized in this study comprised 322 independent study arms involving 13,228 individuals with NDDs. Geographically, the literature was concentrated on a small number of countries, with the largest number of studies conducted in China (*n* = 78), Iran (*n* = 59), and Turkey (*n* = 26); in contrast, reports from Africa were scarce (Fig. 2A). The mean participant age was 13.96 years (range, 3.19–63.42; SD = 9.64), with a weighted male proportion of 71.79% (male-to-female ratio, approximately 2.54:1). The study population was predominantly children and adolescents, with studies enrolling participants aged 3–18 years accounting for 84% (*n* = 268). With respect to NDD subtypes, most studies have focused on intellectual developmental disorders (*n* = 126), autism spectrum disorders (*n* = 103), and attention-deficit/hyperactivity disorders (*n* = 62). In contrast, research on developmental coordination disorders, specific learning disorders, and comorbid conditions is limited, with only 31 studies.

The intervention variables in the included studies are shown in Fig. 2B. Overall, neuromuscular training was the most common intervention type (*n* = 157), followed by aerobic training (*n* = 81) and combined interventions (*n* = 71). In contrast, resistance/strengthening training was less common, appearing in only 13 studies. Interventions were delivered primarily in clinical, classroom-based, or home/community settings, and were most often undertaken by clinicians or educators/coaches not involved in the study design. More than half of the studies used a group-based delivery format. The mean intervention duration was 2.8 ± 1.2 sessions per week, the mean session duration was 50.1 ± 18.1 min, the mean total number of sessions was 31 ± 22.6, and the mean total intervention duration was 1606.5 ± 1479.0 min. Notably, approximately 70% of the studies did not explicitly report intervention intensity. Detailed participant information and the intervention study characteristics are presented in Supplementary Table 5.

### Current Status and Characteristics of Adverse Event and Adverse Effect Reporting

None of the 309 studies fully adhered to the relevant recommendations of the CONSORT Harms Extension, including the use of prespecified monitoring procedures and active strategies to report potential harms associated with physical activity interventions systematically. Only 58 studies described procedures for adverse event monitoring. Of these, 44 used passive, non-prespecified approaches that relied primarily on spontaneous participant reporting. In contrast, only 14 studies reported prespecified adverse event monitoring procedures (Table 1). Notably, only one study assessed adverse events during the post-intervention follow-up to identify delayed harm or harm not captured during the intervention period [46].

**Table 1:** Prespecified adverse event monitoring procedures and adverse events/adverse effects in the physical activity intervention groups

Study	Age	NDD subtype	Intervention type	AE assessment	Reported prespecified AE monitoring and AE/AR reporting procedures
Shields et al. [47]	26.80	IDD	Resistance/strength training	Prespecified	Adverse events were prospectively documented by coaches in participants’ training logs, with standardized injury/issue assessments conducted pre- and post-session. No serious adverse events occurred. Four participants reported transient, training-related mild muscle soreness
Shields et al. [48]	15.60	IDD	Resistance/strength training	Prespecified	All adverse events were systematically documented in the training logs, and participants were proactively queried at both session initiation and completion regarding any injuries or other discomfort. Early in the program, 4–5 participants reported mild muscle soreness, and three reported hand soreness
Shields et al. [49]	18.00	IDD	Resistance/strength training	Prespecified	Detailed information on all injuries or issues (adverse events), as well as any absences (missed sessions), was recorded in the logbook. No sessions were missed due to protocol-related muscle soreness, injury, or illness
Melville et al. [46]	46.22	IDD	Aerobic training	Prespecified	Safety was monitored via participant- and caregiver-reported adverse events and researcher-led active inquiry at each data-collection time point. No trial-related adverse events were reported during the study
Shields et al. [50]	21.40	IDD	Aerobic training	Prespecified	The logbook included an incident section to document details of any injuries/problems (adverse events) and missed sessions. No adverse events occurred
Maharaj et al. [54]	10.02	DCD	Combinations	Prespecified	A first-aid kit was available for minor injuries, and a nurse and physician were on standby to manage any adverse effects during activities. No protocol deviations or adverse effects occurred
Altaye et al. [55]	14.41	IDD	Aerobic training	Prespecified	Participants and their parents/guardians could reduce exercise intensity or stop at any time and were instructed to report any discomfort promptly; if symptoms occurred during exercise, the trainer could reduce intensity or terminate the session as needed
Wang et al. [51]	14.17	IDD	Combinations	Prespecified	Potential intervention-related adverse effects were continuously monitored and documented; none were reported by participants during the study
Svedell et al. [57]	37.00	ADHD	Combinations	Prespecified	A study-developed, prespecified “Participant Experience” questionnaire assessed burden, time demands, adherence/compliance, and perceived well-being changes. Four participants reported stress related to make-up sessions
Zhao et al. [56]	8.40	ADHD	Neuromuscular training	Prespecified	Caregivers were instructed to promptly report any discomfort or adverse effects during the intervention. One child developed fever after three sessions and withdrew at the caregiver’s request
Siddiqui et al. [58]	7.98	IDD	Neuromuscular training	Prespecified	Safety measures included pre- and post-session recording of blood pressure and heart rate
Dhingra et al. [52]	9.75	ASD	Aerobic training	Prespecified	An exercise diary documented the total number of sessions, reasons for absenteeism, adverse-event incidence, and the number of repetitions
Wang et al. [59]	7.69	ASD	Neuromuscular training	Prespecified	Heart rate was monitored during exercise using a Polar heart-rate monitor to prevent excessive training intensity. Exercise was stopped immediately if symptoms occurred (e.g., dyspnea, dizziness, or abnormal sweating)
Li et al. [53]	8.40	ADHD	Neuromuscular training	Prespecified	No adverse events were reported throughout the study period. Safety outcomes, as one of the key outcomes, included adverse effects and events recorded during the intervention period
van Schijndel-Speet et al. [62]	58.05	IDD	Combinations	Non-prespecified	Three participants experienced falls during physical activity, and one participant with comorbid diabetes had an early-stage hypoglycemic episode
Mills et al. [63]	8.72	ASD	Combinations	Non-prespecified	One child released bodily fluids in the swimming pool, and another developed bilateral periknee bruising after the first hydrotherapy session
Obrusnikova et al. [61]	24.60	IDD	Resistance/strength training	Non-prespecified	Two participants reported mild muscle soreness after resistance training; two reported discomfort with physical prompting; two reported discomfort from prolonged headphone use; and one reported discomfort during stair-related tasks
Marzouki et al. [66]	6.34	ASD	Aerobic training	Non-prespecified	During the experimental period, 6 participants developed health issues not attributable to the training program
Downs et al. [65]	20.00	ASD	Aerobic training	Non-prespecified	Three minor adverse events were attributed to increased study-related activity (rash, blistering, hip pain). Two seizures and one lower respiratory tract infection were judged unrelated to the intervention
Tascioglu et al. [67]	9.89	ADHD	Resistance/strength training	Non-prespecified	One participant was diagnosed with calcaneal apophysitis
Zhu et al. [64]	8.53	ADHD	Neuromuscular training	Non-prespecified	One child sustained a left ankle sprain during exercise

*ADHD* attention-deficit/hyperactivity disorder, *ASD* autism spectrum disorder, *DCD* developmental coordination disorder, *IDD* intellectual developmental disorder, *NR* not reported

Overall, adverse event monitoring in these studies relied primarily on training-embedded recordings supplemented by follow-up queries and physiological monitoring. Specifically, seven studies used embedded monitoring frameworks with structured diaries to continuously record adverse events during training, enabling the capture of minor reactions (e.g., delayed-onset muscle soreness) [47–53]. Another six studies collected participant and caregiver reports at prespecified data collection time points, combined them with active researcher querying, and specified explicit intervention stopping rules [46–48, 54–56]. In addition, only three studies incorporated objective physiological indicators (e.g., blood pressure, heart rate, or exercise heart rate) to monitor training intensity during sessions, triggering stopping criteria when prespecified thresholds were reached; these studies also used experience questionnaires to assess intervention burden and related psychological responses [57–59]. Notably, four studies stated in the Methods section that prespecified adverse event monitoring procedures were in place. However, they did not report implementation details or findings in the Results or Discussion section.

Among the 54 studies that reported adverse event monitoring results, 41 explicitly reported that no adverse events were observed, whereas one study provided only a general statement that “a small number of adverse events were observed” [60]. In the physical activity intervention group, 12 studies reported adverse events or adverse effects during the intervention period (Table 1). Three resistance/strengthening training studies reported 11 cases of delayed-onset muscle soreness (adverse effects) during the intervention [47, 48, 61]. Two multicomponent training studies and one neuromuscular training study reported six mild injury- or pain-related events (adverse effects), primarily falls, a left-foot sprain, periknee bruising, and hypoglycemic episodes [62–64]. One aerobic-focused study reported that, as activity volume increased, three participants developed a rash, blisters, or hip pain (adverse effects) [65]. Three additional studies reported that seven participants experienced psychological distress during the intervention, which was attributed to overly rapid session progression, discomfort from wearing sports headphones, and difficulty adapting to the pool environment (adverse effects) [57, 61, 63]. In addition, four studies reported eight participants with fever, calcaneal apophysitis, seizures, lower respiratory tract infection, and other health problems; however, the authors explicitly stated that these events were not related to the physical activity intervention and were therefore classified as non-intervention-related adverse events [56, 65–67].

### Withdrawal Reporting and Potential Safety Signals

In the main text, 201 studies reported participant withdrawal or loss to follow-up. The quality and interpretability of withdrawal reason reporting varied substantially. Specifically, 133 studies reported reasons that did not involve adverse events, 38 used descriptions that were too vague to determine whether withdrawals were related to adverse events or adverse effects (e.g., stating that participants “withdrew for personal reasons”), and four reported withdrawal reasons that involved adverse events but did not specify the affected study group, precluding group-level classification and comparison [68–71].

With respect to the physical activity intervention groups, 16 studies reported at least one adverse event-related item related to withdrawal reasons (Table 2). Specifically, two studies reported that two participants were lost to follow-up due to fractures at different sites [72, 73]; 11 studies reported that 19 participants were lost to follow-up due to health problems [46, 56, 58, 62, 66, 74–79]; two studies reported that six participants discontinued the intervention for COVID-19-related reasons [67, 80]; and one study reported that one participant discontinued because of fear of horses [81]. Importantly, none of the included studies described a systematic procedure for determining withdrawal reasons. Instead, withdrawal reasons were typically presented as brief outcome statements (e.g., “withdrawal due to illness”) and generally lacked temporal information linking events to intervention exposure, clinical detail, and other elements needed to support causal inference (e.g., event timing, severity, management and outcome, and assessment of suspected intervention relatedness). Accordingly, the available reporting is insufficient to attribute specific adverse events to the physical activity intervention in a causal manner. In addition, only four studies showed overlap between adverse event reporting and withdrawal reasons, such that withdrawal reasons were also reported as adverse events. Specifically, these studies indicated in the CONSORT flow diagram that participants withdrew due to the corresponding adverse events, and then described these events in the main text [56, 62, 66, 82]. Finally, in four additional studies, withdrawal reasons for 10 participants were coded as adverse events (health problems), yet the reports also stated that “no adverse events occurred” [46, 74, 78, 83].

**Table 2:** Reasons for withdrawal associated with adverse events

Study	Age	NDD subtype	Intervention type	Reasons for participant withdrawal
El Kafy et al. [73]	9.36	IDD	Aerobic training	One participant discontinued the intervention due to a right elbow fracture
Melville et al. [46]	46.22	IDD	Aerobic training	One participant withdrew due to poor health
Boer et al. [74]	33.30	IDD	Aerobic training	Two participants withdrew due to serious illness
van Schijndel-Speet et al. [62]	58.05	IDD	Combinations	Two participants withdrew due to chronic illness and illness-related issues
Nissim et al. [75]	58.60	IDD	Neuromuscular training	One participant withdrew from the study on medical advice
Cortés-Amador et al. [72]	50.19	IDD	Neuromuscular training	One participant was lost to follow-up after sustaining a patellar fracture from a fall at home
Regaieg et al. [76]	8.85	IDD	Neuromuscular training	Three participants withdrew due to health-related issues
Sansi et al. [77]	8.51	ASD	Neuromuscular training	One female student with ASD withdrew due to health-related issues
Marzouki et al. [66]	6.34	ASD	Aerobic training	Six participants were excluded from the trial due to health issues unrelated to the training protocol
Zhao et al. [81]	5.90	ASD	Neuromuscular training	One participant withdrew due to fear of horses
Gutiérrez-Cruz et al. [80]	34.21	IDD	Resistance/strength training	Three participants discontinued the intervention due to COVID-19 infection
Steyn et al. [78]	44.50	IDD	Combinations	Three participants withdrew due to injury and a family crisis
Tasciogluet al. [67]	9.89	ADHD	Resistance/strength training	Three participants withdrew due to the COVID-19 pandemic
Zhao et al. [56]	8.40	ADHD	Neuromuscular training	One participant discontinued the intervention due to illness (fever)
Siddiqui et al. [58]	7.98	IDD	Neuromuscular training	Two participants discontinued the intervention due to health concerns
Kanzari et al. [79]	8.11	ASD	Combinations	One participant discontinued treatment due to illness

*ADHD* attention-deficit/hyperactivity disorder, *ASD* autism spectrum disorder, *DCD* developmental coordination disorder, *IDD* intellectual developmental disorder, *NR* not reported

### Relative Risk of Adverse Events and Potential Moderators

Because withdrawal events reported in the existing literature generally lacked key information needed to assess their relationship to intervention exposure, they were not included as adverse events in the quantitative synthesis of the meta-analysis. The pooled analysis indicated that compared with non-exercising controls, physical activity interventions did not significantly increase the risk of adverse events (RR = 1.14; 95% CI 0.67–1.94; *I*^2^ = 2%; *p* = 0.62; low GRADE). Meta-regression further revealed that age, modeled as a study-level covariate, did not significantly moderate the relative risk (RR) of adverse events (*β* = 1.01; 95% CI 0.97–1.04; *p* = 0.54).

**Fig. 3:**
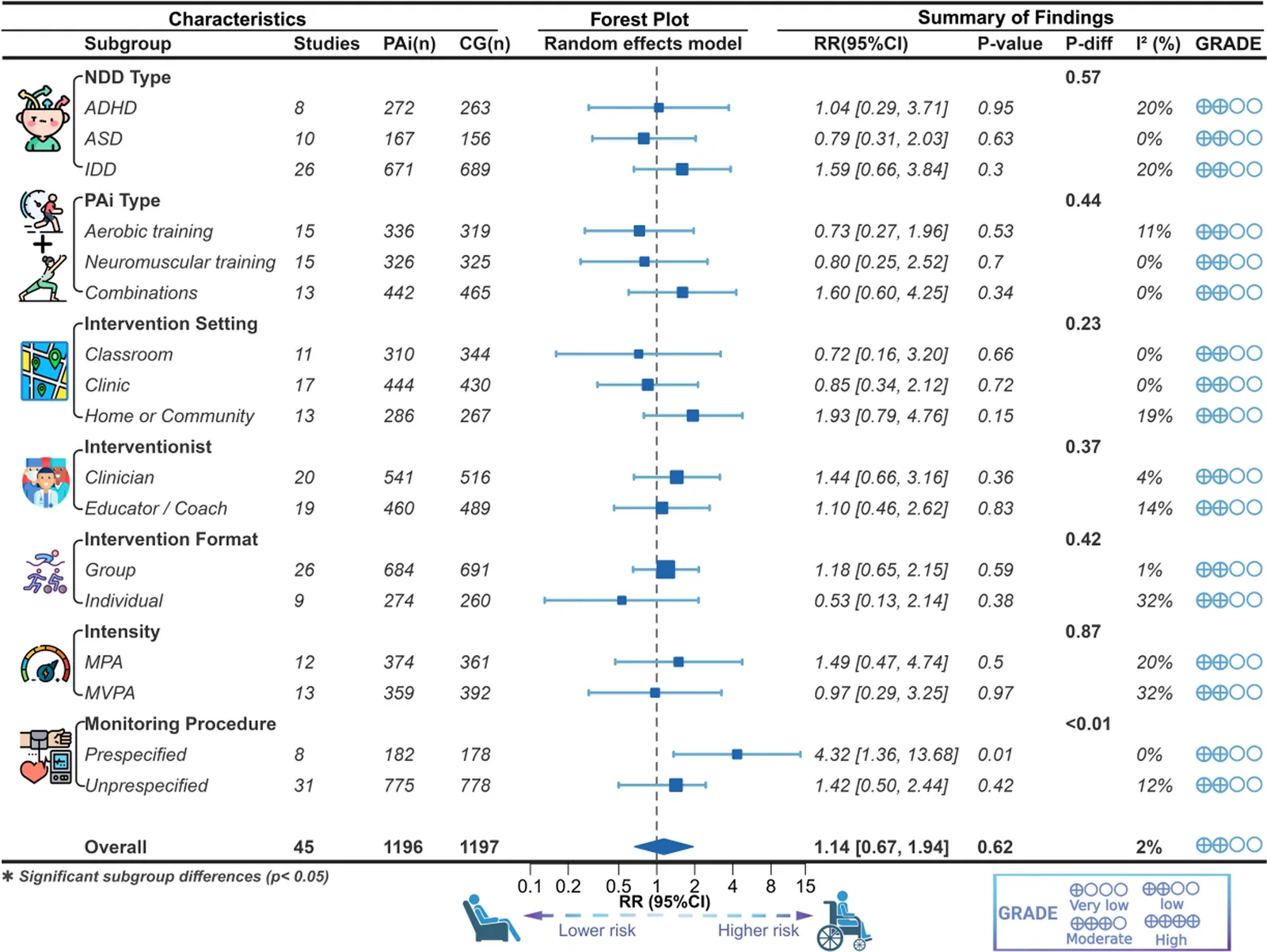
Forest plot of relative risk (RR) for adverse events, subgrouped by primary study characteristics. *ADHD* attention-deficit/hyperactivity disorder, *ASD* autism spectrum disorder, *CG* control group, *GRADE* Grading of Recommendations Assessment, Development, and Evaluation (a system for evaluating the quality of evidence and strength of recommendations), *IDD* intellectual developmental disorder, *MPA* moderate-intensity physical activity, *MVPA* moderate-to-vigorous physical activity, *PAi* physical activity intervention, *P value* statistically significant *p* values for pooled results, *I*^*2*^ quantitative indicators of heterogeneity, *95% CI* 95% confidence interval

Given that the limited number of included studies may have reduced the statistical power and increased the risk of false-negative findings, subgroup analyses were restricted to strata with ≥5 studies [84]. We compared the results by NDD type, physical activity type, intervention setting, intervention provider, delivery format, intervention intensity, and adverse event monitoring procedures. Statistically significant effects and clear between-subgroup differences were observed only for studies with prespecified adverse event monitoring procedures (RR = 4.32; 95% CI 1.36–13.68; *I*^2^ = 0%; *p* = 0.01; *p* for interaction < 0.01; low GRADE). The corresponding meta-regression indicated that this moderator explained 90% of the between-study variance in effect sizes. This finding suggests that the observed difference more likely reflects more complete detection and reporting of adverse events under prespecified monitoring procedures rather than an increase in the underlying true risk. In contrast, none of the remaining subgroup strata yielded statistically significant effects, and no additional between-subgroup differences were observed (Fig. 3).

### Dose‒Response Relationship

**Fig. 4:**
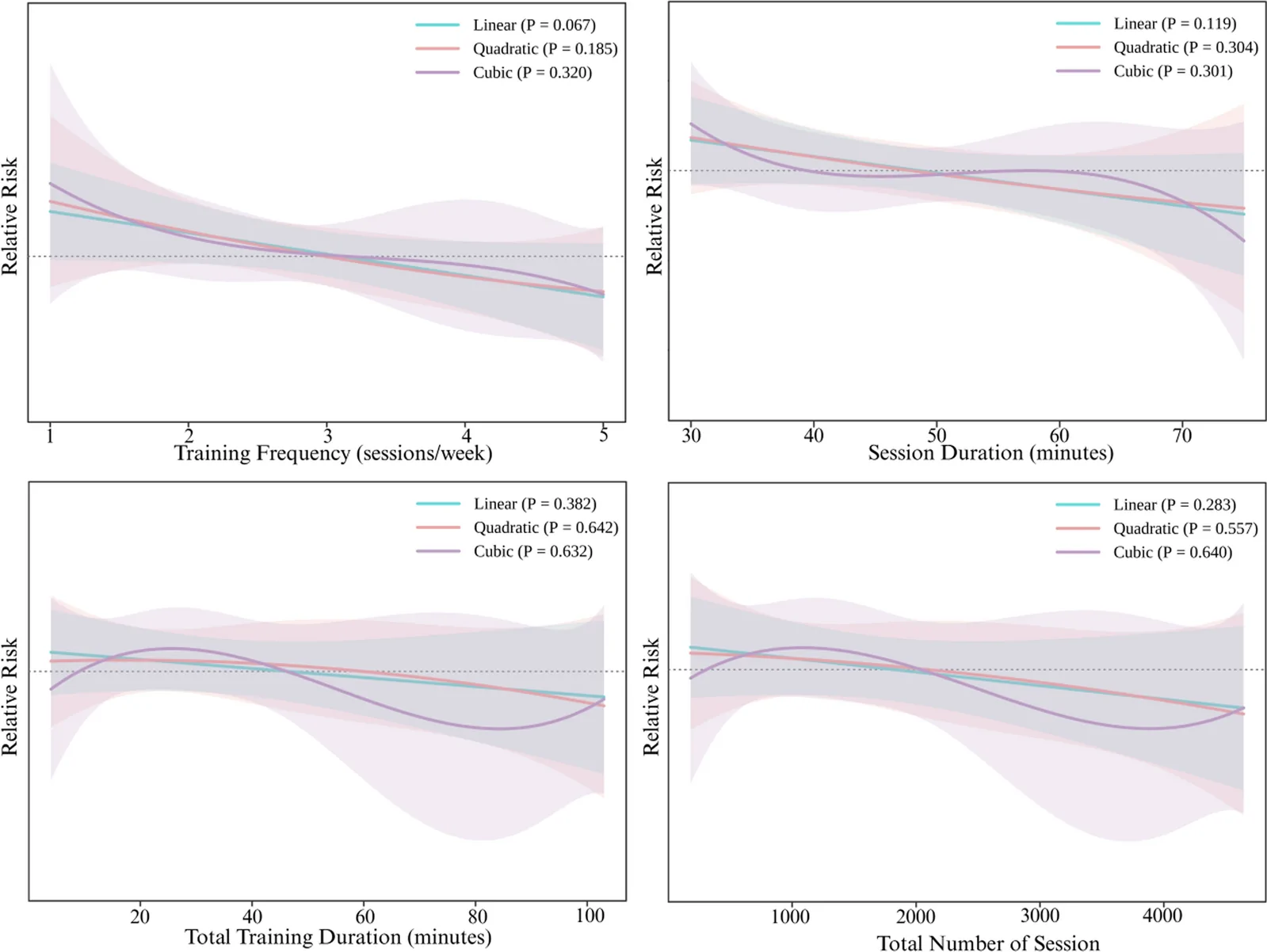
Associations between adverse events and physical activity intervention dose (weeks and minutes), with shaded areas indicating 95% confidence intervals for each model

Dose–response analyses indicated that, regardless of how intervention dose was modeled (i.e., weekly training frequency, session duration, total number of sessions, or total training time), no statistically significant association was found with the relative risk (RR) of adverse events (Fig. 4). Meta-regression further supported these results, indicating that the included covariates did not significantly explain between-study heterogeneity or reduce between-study variance (*p* = 0.07–0.54). However, given the limited number of eligible studies and the suboptimal quality of adverse event reporting, these findings should not be interpreted as evidence that no dose–response relationship exists. In addition, because few of the included studies reported exercise intensity, we could not quantitatively evaluate the association between exercise intensity and adverse event risk.

### Risk of Bias

The risk-of-bias assessments for each study are presented in Supplementary Fig. 5. Most studies did not meet the standards for allocation concealment, and key details regarding random sequence generation and allocation implementation were not adequately reported. Accordingly, the randomization process domain was generally rated as of some concern, with one study rated as high risk because the authors explicitly acknowledged deficiencies in the allocation design [71]. In addition, attrition was substantial, and some studies did not include complete datasets in intention-to-treat (ITT) analyses; as a result, approximately 20% of the studies were rated as some concerns in the missing outcome data domain. For outcome measurement, nearly half of the studies did not clearly report blinding procedures, did not implement blinding, or did not include safeguards to maintain blinding feasibility, and were therefore also rated as having some concerns. In contrast, only one study preregistered its protocol in a public registry and reported results consistent with its prespecified analyses, including a predefined adverse event-monitoring procedure. However, most studies were either not preregistered or provided incomplete preregistration information, thereby increasing the risk of selective outcome reporting [46]. Overall, reporting was insufficient and/or implementation was suboptimal across multiple domains, and the overall risk of bias was predominantly judged as some concerns.

### Publication Bias and Sensitivity Analyses

Publication bias was evaluated via contour-enhanced funnel plots in combination with multiple statistical tests. Overall, the funnel plot appeared reasonably symmetric, providing preliminary evidence of no clear publication bias (Supplementary Fig. 6). The results of the quantitative tests were consistent with this pattern. Egger’s linear regression test detected no significant small-study effects or funnel plot asymmetry (*P* = 0.621), and Begg’s rank correlation test (Kendall’s *τ*) did not indicate asymmetry (*P* = 0.655). Notably, Peters’ regression test, which is more robust for binary outcomes, was marginally significant (*P* = 0.053), suggesting a possible mild tendency toward small-study effects.

We conducted sensitivity analyses via multiple statistical models to evaluate whether the conclusions were robust to different pooling strategies and approaches for handling zero-event studies. The results were highly consistent across methods. Using the Mantel–Haenszel (MH) approach with the Battaglia continuity correction, the pooled relative risk (RR) was 1.13 (95% CI 0.67–1.90; *I*^2^ = 1%; *P* = 0.65). Under inverse-variance models, the pooled RR remained at approximately 1.13, regardless of whether *τ*^2^ was estimated via restricted maximum likelihood (REML) or the DerSimonian–Laird (DL) estimator, with comparable confidence intervals and heterogeneity (95% CI ≈ 0.67–1.91; *I*^2^ = 1%; *P* ≈ 0.65–0.66). These findings were consistent with the primary analysis (MH + TACC: RR = 1.14; 95% CI 0.67–1.94; *I*^2^ = 2%; *P* = 0.62), suggesting that the conclusions were not dependent on a specific pooling method or continuity-correction approach.

## Discussion

This study systematically synthesized 309 randomized controlled trials, including 13,228 participants with NDDs, by collating primary study reports of adverse events, adverse effects, or other harms (see the graphical abstract in Supplementary Fig. 1). We also incorporated withdrawal reasons that could reasonably be classified as adverse events or adverse effects to extract and summarize adverse event-related data. To our knowledge, this study is the first to systematically review and quantitatively synthesize safety evidence on physical activity interventions for individuals with NDDs from an adverse event-reporting perspective. Our findings indicate that although the number of studies and the geographic scope of the evidence base have increased substantially in recent years, most trials did not proactively prespecify adverse event monitoring procedures. Moreover, even when adverse events or adverse effects occur, they are often not labeled or reported in a standardized and routine manner. The primary meta-analysis of studies reporting adverse events indicated that, compared with non-exercising controls, physical activity interventions did not increase the overall risk of reported adverse events. However, the strength of this conclusion is limited by the widespread underreporting of such events in the original studies and by substantial heterogeneity in methods for harm monitoring, identification, and assessment. In addition, studies that implemented prespecified adverse event monitoring procedures generally identified and reported relevant adverse events more comprehensively.

In the included studies, adverse events primarily reflected participants’ health problems and family crises, whereas adverse effects fell into several categories. These included delayed-onset muscle soreness in resistance/strength training; minor falls, sprains, bruising, or hypoglycemic episodes during combined neuromuscular training; rashes, blisters, or hip pain associated with increasing exercise volume during aerobic training; and psychological discomfort related to sensory stimulation or environmental adaptation, such as discomfort from wearing devices, an overly rapid pace, and difficulty adapting to the pool environment. Although only approximately 17% of the studies reported adverse event monitoring procedures and results, among these studies, nearly one quarter reported at least one adverse event or adverse effect. Similarly, among studies reporting reasons for participant withdrawal, a comparable proportion described adverse events or adverse effects. More importantly, most studies relied on passive, non-prespecified adverse event monitoring (e.g., primarily participant self-reports), which may be insufficient to capture NDD-specific differences in recognizing and expressing exercise tolerance, pain experiences and observable manifestations, fluctuations in adherence over time, and adaptation to unfamiliar settings or intervention procedures [85–89]. Because individuals with NDDs may have difficulty identifying and communicating internal states, exclusive reliance on passive, unstructured monitoring may lead to missed or misclassified adverse events and to systematic underestimation at the study level [90–92]. Given these limitations, the true rate of intervention-related adverse events in physical activity interventions for individuals with NDDs may be higher than that currently reported in the existing literature. Despite clear underreporting in the current evidence base, the available data still suggest that approximately one quarter of the intervention studies documented adverse events. In addition, the CONSORT 2025 guidelines explicitly require standardized, transparent reporting of participant withdrawals, attrition, and loss to follow-up [16]. Specifically, investigators should report withdrawal reasons for each group and provide clear descriptions of withdrawal events, distinguishing attrition related to the intervention from attrition due to other causes. However, in the included studies, almost none used a structured, traceable procedure to collect and adjudicate withdrawal reasons, or to handle vague descriptions such as “personal reasons” or “health problems”. This limitation also prevented further evaluation of the adverse effects. This may have two consequences. First, truly intervention-related adverse effects may be embedded in withdrawal reasons and therefore go unrecognized. Second, if withdrawals reflect intervention tolerability and differ between groups, the efficacy estimates may be systematically biased, undermining the accuracy and credibility of the study findings [93].

Consistent with a prior meta-analysis of 375 primary exercise studies that excluded NDD populations, physical activity interventions did not significantly increase overall adverse event risk compared with non-exercising controls (RR = 1.14, 95% CI 0.67–1.94), and no statistically significant differences were observed by age or intervention dose [24]. However, the absence of significant differences across NDD subtypes should not be interpreted as evidence of identical risk profiles. A descriptive appraisal of the reported events suggested that adverse events among individuals with IDD predominantly involved physical or medical problems [47, 62]. Reports involving individuals with ASD included discomfort associated with specific intervention environments, such as difficulties adapting to aquatic exercise and equine-assisted interventions [63, 81], whereas the limited evidence for ADHD included psychological burden associated with intervention arrangements [56]. Evidence for other NDD subtypes was insufficient to characterize their potential adverse event profiles. These preliminary patterns may inform subtype-specific safety monitoring strategies, with greater attention to physical symptoms and medical risks in IDD, sensory responses and environmental adaptation in ASD, and psychological burden and intervention-related stress in ADHD. Nevertheless, given the limited and unevenly distributed evidence base and the generally incomplete reporting of adverse events, these observations should be considered exploratory and require validation in large-scale prospective studies using standardized monitoring procedures. By contrast, prespecified adverse event monitoring was the only significant moderator identified, with a higher observed risk among studies using such procedures (RR = 4.32, 95% CI 1.36–13.68); this moderator explained a substantial proportion of the between-study variability in effect sizes. This finding is consistent with recent reviews of exercise intervention efficacy that have highlighted substantial gaps in safety data collection and reporting in primary studies, suggesting that the apparent “safety” of physical activity interventions may reflect incomplete ascertainment and reporting of adverse events [94–96]. When studies actively seek adverse events by training logs, proactive querying at prespecified time points, physiological monitoring, and explicit stopping thresholds, participant symptoms, including mild muscle soreness, transient pain, friction-related skin irritation, and emotional discomfort, are more likely to be detected and recorded. In contrast, studies that rely solely on spontaneous reports from participants or caregivers often overlook or fail to formally report minor events. This pattern aligns with the central logic of the CONSORT Harms framework: more proactive and structured monitoring is more likely to identify mild but genuine harm [17]. Therefore, this finding suggests that prespecified, systematic, and proactive monitoring makes real but mild adverse events visible rather than indicating that monitoring itself increases risk [97].

In summary, the shortcomings in adverse event reporting and handling of participant withdrawal in this field likely reflect the interplay of multiple factors rather than a single deficiency. Specifically, some studies did not establish a systematic operational framework for adverse event monitoring during protocol development, resulting in subsequent monitoring procedures that lacked consistent standards or traceability [98, 99]. In addition, during trial implementation, adverse events and withdrawal data may not have been consistently collected and documented, weakening the completeness and verifiability of the evidence base [100]. Even when such information is collected, the apparent “invisibility” or marked attenuation of adverse events in the literature may also reflect conflicts of interest [101]. Although the present study did not formally assess these factors, previous research suggests that when particular conclusions confer academic, professional, or resource-related benefits, researchers may selectively disclose information or report it incompletely, resulting in restricted reporting [102, 103]. In addition, findings may be presented in ways that do not align with the underlying data, or existing data may be omitted intentionally or unintentionally, creating systematic information gaps [104]. Additionally, in physical activity intervention research, social desirability bias may reduce investigators’ sensitivity to risk signals and shape interpretive framing, leading adverse events and withdrawal information to be downplayed in reporting [105]. Finally, the lack of mandatory requirements and unified journal standards for adverse event reporting may weaken external oversight and accountability, allowing these problems to persist and become entrenched in the publication process, thereby contributing to a cumulative, snowball-like cycle [106].

### Future Directions and Recommendations

In current physical activity intervention studies involving individuals with NDDs, the reporting of adverse events and withdrawals is often insufficient to determine what occurred or to classify incidents as adverse events or adverse effects. Therefore, future randomized controlled trials in this field should not only systematically monitor and report adverse events and adverse effects, but also transparently incorporate this monitoring framework into the study protocol from the outset as a core element of study design. Specifically, study protocols should prespecify general active monitoring procedures applicable to a broad range of physical activity interventions. Examples include brief pre-session health screening (e.g., acute discomfort, pain, insufficient sleep, and medication changes), objective indicators and structured recording of adverse events during training (e.g., heart rate, heart rate variability, and stress-related physiological markers), and proactive post-session follow-up to assess potentially delayed responses (e.g., delayed-onset muscle soreness, accumulated fatigue, or functional limitations). Second, in addition to general monitoring, intervention-specific active surveillance should be implemented as needed. Examples include tracking sensory responses to horses, fear or avoidance behaviors in equine-assisted therapy, and monitoring movement compensation and technique deviations across muscle groups during resistance training. Finally, studies should incorporate spontaneous-report monitoring, with caregiver proxy reporting when appropriate, to capture additional adverse events not anticipated or prespecified in the protocol, thereby reducing underreporting and strengthening the completeness and credibility of safety evidence.

Multiple studies consistently indicate that intervention intensity is a key determinant of adverse event risk during physical activity interventions [107, 108]. However, nearly 70% of studies have not adequately documented or reported intervention intensity, limiting the interpretation of risk attribution across studies and the assessment of dose–response relationships. Therefore, after implementing systematic monitoring procedures, future RCTs should also report key “denominator information” in real-world physical activity intervention contexts. Specifically, the planned dose specified at the design stage and the delivered dose achieved during implementation should be clearly defined and reported transparently and consistently, using standardized conventions, to minimize bias and misinterpretation [109]. At minimum, studies should report the per-group sample size included in the final analysis and the inclusion and exclusion process; participants’ baseline characteristics, including key demographic and clinical features; discrepancies between planned and delivered doses and the reasons for those discrepancies; and core contextual and implementation variables during delivery (e.g., setting, provider qualifications and training, delivery format, adherence, and fidelity) [110–112]. Full disclosure of this “denominator information” would improve comparability and reproducibility across studies, enabling more refined stratified analyses and tests of effect modification. For example, such reporting could help identify NDD subgroups more likely to experience adverse events under specific intervention conditions, thereby supporting risk stratification, optimizing monitoring strategies, and enabling individualized dose adjustments [15].

Although the CONSORT Harms statement recommends reporting group-specific effect estimates and their precision (e.g., confidence intervals) for adverse events, Ioannidis [113] noted that statistically significant between-group differences are often difficult to detect for a single adverse event [17, 113]. He further cautioned that aggregating adverse events that differ in nature, severity, or mechanisms into a single overall adverse event outcome to increase statistical power can increase heterogeneity in outcome definitions, thereby reducing interpretability and clinical relevance [113]. Based on these considerations, future RCTs should systematically collect and transparently report participant-level descriptive data on adverse events to generate high-quality evidence that is both informative and clinically meaningful [106]. Furthermore, if primary studies provide adequate and transparent reporting of adverse events, subsequent meta-analyses can aggregate and stratify these data across studies, enabling more robust and verifiable inferences about potential associations between physical activity interventions and adverse events, with greater statistical power and stronger external validity [113].

### Potential Limitations

Although this study provides a systematic and comprehensive evaluation of adverse event monitoring and reporting practices in physical activity interventions for individuals with NDDs, several limitations are unavoidable and should be addressed in future research. First, instead of fully adopting Niemeijer et al.’s classification of adverse events as non-serious or serious, we distinguished between adverse events and adverse effects [24]. This decision was based on the fact that individuals with NDDs often have varying degrees of cognitive impairment and adaptive behavior difficulties that can substantially affect daily functioning, making severity judgments difficult to apply and compare with those used for typically developing populations using the same thresholds [15, 20]. Accordingly, mechanically applying existing severity grading criteria may rely on insufficiently validated assumptions and increase the risk of misclassification. Second, classifying an adverse event as intervention-related is inherently subjective to some extent, and validity may vary by reporter perspective and study context. To minimize subjectivity, our coding followed the definitions and operational guidance of the Cochrane Handbook [34]. The high interrater agreement (Cohen’s *κ* = 0.85) supports the robustness and reproducibility of our classification and synthesis. Future research should develop severity grading standards and attribution procedures better tailored to adverse events in NDD populations to improve the precision of adverse event monitoring and reporting and to enhance cross-study comparability. Third, given the pervasive underreporting in the original studies and the substantial heterogeneity in methods used for harm monitoring, identification, and assessment, the pooled quantitative findings of the present study should be interpreted as reflecting “a lack of evidence for excess risk” rather than “evidence of the absence of excess risk”. Nevertheless, a preliminary quantitative synthesis based on the currently available evidence remains both necessary and practically meaningful. On the one hand, systematic evidence on adverse event reporting for physical activity interventions in NDD populations remains scarce; avoiding quantitative synthesis solely because primary reporting is imperfect could further fragment the evidence base and delay the accumulation and translation of safety evidence into practice. Moreover, our synthesis extended beyond conventional study descriptors, such as participant characteristics and implementation features, by evaluating adverse event monitoring procedures. These quantitative findings were consistent with our critical appraisal, underscoring the need for future studies to prespecify systematic and standardized adverse event monitoring procedures with clear operational definitions at the protocol stage.

## Conclusion

In recent years, research on physical activity interventions for individuals with NDDs has increased substantially, and existing reported results suggest that these interventions do not significantly increase the overall risk of adverse events. However, the strength of this conclusion is limited by widespread systematic underreporting in the original studies and by substantial heterogeneity in methods used to monitor, identify, and assess harms. In contrast, studies that implemented prespecified adverse event monitoring procedures generally identified and reported relevant adverse events more comprehensively. Therefore, clinicians and guideline developers should continue to interpret the current evidence on safety with caution. When evaluating the benefit–risk profile of physical activity interventions for individuals with NDDs, greater weight should be given to trials that used prespecified, active adverse event monitoring. Future research should establish and implement standardized procedures for adverse event monitoring and reporting, standardize procedures for adjudicating and attributing reasons for withdrawal, and systematically report key variables related to intervention implementation and descriptive adverse event data to strengthen the credibility of benefit–risk assessments of physical activity interventions in this population.

## Supplementary Information

## Data Availability

The data and code supporting this study were uploaded to Open Science Framework (https://osf.io/b9ta2/overview).

## Abbreviations

ADHD: Attention-deficit/hyperactivity disorder
AE: Adverse event
AR: Adverse reaction
ASD: Autism spectrum disorder
AT: Aerobic training
CG: Control group
CONSORT: Consolidated Standards of Reporting Trials
DCD: Developmental coordination disorder
GRADE: Grading of Recommendations, Assessment, Development, and Evaluation
IDD: Intellectual developmental disorder
ITT: Intention-to-treat
MPA: Moderate-intensity physical activity
MVPA: Moderate-to-vigorous physical activity
NDD: Neurodevelopmental disorder
NT: Neuromuscular training
PAi: Physical activity intervention
PICOS: Population, Intervention, Comparison, Outcomes, and Study Design
PRISMA: Preferred Reporting Items for Systematic Reviews and Meta-Analyses
PROSPERO: International Prospective Register of Systematic Reviews
RCT: Randomized controlled trial
RoB 2: Risk of Bias 2
RR: Relative risk
RT: Resistance/strengthening training
SLD: Specific learning disorder
