# Supplementary material for: Invisible Harms, Visible Benefits? Adverse Event Reporting in Physical Activity Interventions for Neurodevelopmental Disorders: A Meta-analysis and Critical Appraisal
Source: Sports Med Open. 2026 Sep 2;12:129. doi: 10.1186/s40798-026-01095-w (PMC13538331; doi:10.1186/s40798-026-01095-w)
Supplement: Supplementary file 1 [file 40798_2026_1095_MOESM1_ESM.docx]

**Supplementary material** for

**Invisible Harms, Visible Benefits? Adverse Event Reporting in Physical Activity Interventions for Neurodevelopmental Disorders: A Meta-analysis and Critical Appraisal**

**Content**

[1. Graphical abstract	2](#_Toc18333)

[2. PRISMA 2020 Checklist	3](#_Toc30938)

[3. Keyword Selection and Search Strategy	8](#_Toc6675)

[4. ASReview—Rationale, Architecture, and Study-Specific	20](#_Toc28428)

[5. Codebook	23](#_Toc32745)

[6. Supplementary results	32](#_Toc12522)

[References	52](#_Toc31311)

1. **Graphical abstract**

**Supplementary figure 1.** Graphical abstract

2. **PRISMA 2020 Checklist**

**Supplementary Table 1**

| **Section and Topic** | **Item #** | **Checklist item** | **Location where item is reported** |
| --- | --- | --- | --- |
| **TITLE** | | |  |
| Title | 1 | Identify the report as a systematic review. | Page 1  (manuscript) |
| **ABSTRACT** | | |  |
| Abstract | 2 | See the PRISMA 2020 for Abstracts checklist. | Page 1-2  (manuscript) |
| **INTRODUCTION** | | |  |
| Rationale | 3 | Describe the rationale for the review in the context of existing knowledge. | Page 3  (manuscript) |
| Objectives | 4 | Provide an explicit statement of the objective(s) or question(s) the review addresses. | Page 3  (manuscript) |
| **METHODS** | | |  |
| Eligibility criteria | 5 | Specify the inclusion and exclusion criteria for the review and how studies were grouped for the syntheses. | Page 4  (manuscript) Page 19  (supplementary material) |
| Information sources | 6 | Specify all databases, registers, websites, organisations, reference lists and other sources searched or consulted to identify studies. Specify the date when each source was last searched or consulted. | Page 9-18  (supplementary material) |
| Search strategy | 7 | Present the full search strategies for all databases, registers and websites, including any filters and limits used. | Page 9-18  (supplementary material) |
| Selection process | 8 | Specify the methods used to decide whether a study met the inclusion criteria of the review, including how many reviewers screened each record and each report retrieved, whether they worked independently, and if applicable, details of automation tools used in the process. | Page 4-5  (manuscript) |
| Data collection process | 9 | Specify the methods used to collect data from reports, including how many reviewers collected data from each report, whether they worked independently, any processes for obtaining or confirming data from study investigators, and if applicable, details of automation tools used in the process. | Page 5  (manuscript) |
| Data items | 10a | List and define all outcomes for which data were sought. Specify whether all results that were compatible with each outcome domain in each study were sought (e.g. for all measures, time points, analyses), and if not, the methods used to decide which results to collect. | Page 5  (manuscript) |
|  | 10b | List and define all other variables for which data were sought (e.g. participant and intervention characteristics, funding sources). Describe any assumptions made about any missing or unclear information. | Page 22-30  (supplementary material) |
| Study risk of bias assessment | 11 | Specify the methods used to assess risk of bias in the included studies, including details of the tool(s) used, how many reviewers assessed each study and whether they worked independently, and if applicable, details of automation tools used in the process. | Page 5-6  (manuscript) |
| Effect measures | 12 | Specify for each outcome the effect measure(s) (e.g. risk ratio, mean difference) used in the synthesis or presentation of results. | Page 6  (manuscript) |
| Synthesis methods | 13a | Describe the processes used to decide which studies were eligible for each synthesis (e.g. tabulating the study intervention characteristics and comparing against the planned groups for each synthesis (item #5)). | Page 6  (manuscript) |
|  | 13b | Describe any methods required to prepare the data for presentation or synthesis, such as handling of missing summary statistics, or data conversions. | Page 6  (manuscript) |
|  | 13c | Describe any methods used to tabulate or visually display results of individual studies and syntheses. | Page 5-6  (manuscript) |
|  | 13d | Describe any methods used to synthesize results and provide a rationale for the choice(s). If meta-analysis was performed, describe the model(s), method(s) to identify the presence and extent of statistical heterogeneity, and software package(s) used. | Page 5-6  (manuscript) |
|  | 13e | Describe any methods used to explore possible causes of heterogeneity among study results (e.g. subgroup analysis, meta-regression). | Page 5-6  (manuscript) |
|  | 13f | Describe any sensitivity analyses conducted to assess robustness of the synthesized results. | Page 5-6  (manuscript) |
| Reporting bias assessment | 14 | Describe any methods used to assess risk of bias due to missing results in a synthesis (arising from reporting biases). | Page 5-6  (manuscript) |
| Certainty assessment | 15 | Describe any methods used to assess certainty (or confidence) in the body of evidence for an outcome. | Page 6  (manuscript) |
| **RESULTS** | | |  |
| Study selection | 16a | Describe the results of the search and selection process, from the number of records identified in the search to the number of studies included in the review, ideally using a flow diagram. | Page 7  (manuscript) |
|  | 16b | Cite studies that might appear to meet the inclusion criteria, but which were excluded, and explain why they were excluded. | Page 7  (manuscript) |
| Study characteristics | 17 | Cite each included study and present its characteristics. | Page 31-49  (supplementary material) |
| Risk of bias in studies | 18 | Present assessments of risk of bias for each included study. | Page 14  (manuscript) |
| Results of individual studies | 19 | For all outcomes, present, for each study: (a) summary statistics for each group (where appropriate) and (b) an effect estimate and its precision (e.g. confidence/credible interval), ideally using structured tables or plots. | Page 13  (manuscript) |
| Results of syntheses | 20a | For each synthesis, briefly summarise the characteristics and risk of bias among contributing studies. | Page 13-14  (manuscript) |
|  | 20b | Present results of all statistical syntheses conducted. If meta-analysis was done, present for each the summary estimate and its precision (e.g. confidence/credible interval) and measures of statistical heterogeneity. If comparing groups, describe the direction of the effect. | Page 13-14  (manuscript) |
|  | 20c | Present results of all investigations of possible causes of heterogeneity among study results. | Page 13  (manuscript) |
|  | 20d | Present results of all sensitivity analyses conducted to assess the robustness of the synthesized results. | Page 15  (manuscript) |
| Reporting biases | 21 | Present assessments of risk of bias due to missing results (arising from reporting biases) for each synthesis assessed. | Page 15  (manuscript) |
| Certainty of evidence | 22 | Present assessments of certainty (or confidence) in the body of evidence for each outcome assessed. | Page 48  (supplementary material) |
| **DISCUSSION** | | |  |
| Discussion | 23a | Provide a general interpretation of the results in the context of other evidence. | Page 15  (manuscript) |
|  | 23b | Discuss any limitations of the evidence included in the review. | Page 18  (manuscript) |
|  | 23c | Discuss any limitations of the review processes used. | Page 18  (manuscript) |
|  | 23d | Discuss implications of the results for practice, policy, and future research. | Page 17-18  (manuscript) |
| **OTHER INFORMATION** | | |  |
| Registration and protocol | 24a | Provide registration information for the review, including register name and registration number, or state that the review was not registered. | Page 4  (manuscript) |
|  | 24b | Indicate where the review protocol can be accessed, or state that a protocol was not prepared. | Page 4  (manuscript) |
|  | 24c | Describe and explain any amendments to information provided at registration or in the protocol. | Page 4  (manuscript) |
| Support | 25 | Describe sources of financial or non-financial support for the review, and the role of the funders or sponsors in the review. | Page 19  (manuscript) |
| Competing interests | 26 | Declare any competing interests of review authors. | Page 19  (manuscript) |
| Availability of data, code and other materials | 27 | Report which of the following are publicly available and where they can be found: template data collection forms; data extracted from included studies; data used for all analyses; analytic code; any other materials used in the review. | Page 19  (manuscript) |

3. **Keyword Selection and Search Strategy**

**Supplementary Table 2:** MeSH Headings and Free-Text Terms (PICOS Framework)

| **PICOS** | **Scope/MeSH** | **Term (Join with Boolean operator OR)** |
| --- | --- | --- |
| Participants | Neurodevelopmental Disorders | ("neurodevelopmental disorder*" OR "developmental disorder*" OR "autism spectrum disorder*" OR "ASD" OR "autism" OR "asperger*" OR "Pervasive developmental Disorder*" OR "childhood disintegrative disorder" OR "attention deficit hyperactivity disorder*" OR "ADHD" OR "attention deficit disorder*" OR "ADD" OR "attention deficit and disruptive behavior disorder*" OR "intellectual disabilit*" OR "intellectually disabled" OR "mental retardation" OR "intellectual developmental disorder" OR "global developmental delay" OR "specific learning disorder*" OR "learning disabilit*" OR "dyslexia" OR "dyscalculia" OR "Acalculia" OR "reading disorder" OR "mathematics disorder" OR "disorder of written expression" OR "motor disorder*" OR "developmental coordination disorder*" OR "motor skills disorder" OR "stereotypic movement disorder*" OR "communication disorder*" OR "language disorder" OR "phonological disorder" OR "speech sound disorder" OR "stuttering" OR "childhood-onset fluency disorder" OR "tic disorder*" OR "tourette's syndrome" OR "Tourettes disease" OR "Down syndrome" OR "Trisomy 21" OR "Fragile X Syndrome" OR "Martin-Bell syndrome" OR "Williams syndrome" OR "williams beuren syndrome" OR "Prader Willi syndrome" OR "Angelman syndrome" OR "Rett Syndrome" OR "Turners Syndrome" OR "Smith-Magenis syndrome" OR "Tuberous sclerosis" OR "DiGeorge syndrome" OR "velocardiofacial syndrome" OR "22q11 deletion syndrome" OR "Fetal Alcohol Spectrum Disorder*" OR "FASD" OR "cerebral palsy") |
| Intervention | "Physical activity" OR "Exercise" | ("physical activit*" OR "physical fitness" OR "physical exercise" OR "physical education" OR "leisure activit*" OR "motor activit*" OR "sport*" OR "sport* participation" OR "fitness" OR "cardiovascular fitness" OR "athletic*" OR "exercis*" OR "acute exercise" OR "chronic exercise" OR "healthy exercise" OR "yoga" OR "pilates" OR "aerobic exercise" OR "aerobic" OR "aerobic training" OR "resistance exercise" OR "anaerobic exercise" OR "intervention" OR "baseball" OR "football" OR "soccer" OR "basketball" OR "racket sports" OR "racquet sports" OR "badminton" OR "tennis" OR "cricket" OR "track and field" OR "running" OR "sprint" OR "long distance running" OR "jump" OR "bike" OR "bicycling" OR "boxing" OR "taekwondo" OR "judo" OR "golf" OR "gymnastics" OR "hockey" OR "climbing" OR "hiking" OR "Tai Ji" OR "martial arts" OR "swimming" OR "aquatic exercise" OR "aquatic therapy" OR "diving" OR "skating" OR "snow sports" OR "skiing" OR "sports for persons with disabilities" OR "youth sports" OR "wrestling" OR "weight lifting" OR "game*" OR "virtual sports game" OR "recreation*" OR "motor skills" OR "motor intervention" OR "functional exercise" OR "motor learning" OR "behaviour skills training" OR "task specific training" OR "strength training" OR "horse riding" OR "equestrian" OR "equine-assisted therapy" OR "hippotherapy" OR "trampoline" OR "snowshoeing" OR "skating" OR "exergaming" OR "skateboarding" OR "dance" OR "walking" OR "treadmill" OR "karate") |
| Comparison | "Control Groups" OR "Placebos" OR "Standard of Care" OR "Waiting Lists" | ("control group*" OR "comparison group*" OR comparator* OR controlled OR "controlled trial" OR "between-group*" OR "between group*" OR "parallel group*" OR "group design" OR "active control" OR "attention control" OR "contact control" OR "minimal contact" OR "education only" OR "information only" OR "no-contact control" OR "no treatment" OR "no-treatment control" OR "delayed treatment" OR "delayed intervention" OR waitlist* OR "wait-list" OR "waiting list" OR WL OR WLC OR "waitlist control" OR placebo* OR "placebo control*" OR sham OR "sham control*" OR "sham training" OR "sham stimulation" OR "dummy treatment" OR "usual care" OR UC OR "care as usual" OR CAU OR "treatment as usual" OR TAU OR "standard of care" OR "standard care" OR "routine care" OR "usual practice" OR "standard practice" OR "services as usual" OR SAU OR "business as usual" OR BAU OR "enhanced usual care" OR EUC OR "optimized usual care" OR OUC) |
| Outcome | Any outcome | N/A |
| Study  Design | "Randomized Controlled Trials" | (random* OR randomi?ed OR randomi?ation OR "random allocation" OR "random assignment" OR "randomly assigned" OR "randomly allocated" OR RCT OR RCTs OR "randomized controlled trial" OR "randomised controlled trial" OR "randomized trial" OR "randomised trial" OR "randomized clinical trial" OR "randomised clinical trial" OR "clinical trial, randomized" OR "trial, randomized") |

**Supplementary Table 3:** Retrieval Strategy Across Databases

| **Database** | **Search number** | **Query** | **Results** |
| --- | --- | --- | --- |
| Pubmed | 1 | ( "Neurodevelopmental Disorders"[Mesh] OR "neurodevelopmental disorder"[tiab] OR "neurodevelopmental disorders"[tiab] OR "developmental disorder"[tiab] OR "developmental disorders"[tiab] OR "autism spectrum disorder"[tiab] OR "autism spectrum disorders"[tiab] OR ASD[tiab] OR autism[tiab] OR asperger*[tiab] OR "pervasive developmental disorder"[tiab] OR "pervasive developmental disorders"[tiab] OR "childhood disintegrative disorder"[tiab] OR "attention deficit hyperactivity disorder"[tiab] OR "attention deficit hyperactivity disorders"[tiab] OR ADHD[tiab] OR "attention deficit disorder"[tiab] OR "attention deficit disorders"[tiab] OR ADD[tiab] OR "attention deficit and disruptive behavior disorder"[tiab] OR "attention deficit and disruptive behavior disorders"[tiab] OR "intellectual disability"[tiab] OR "intellectual disabilities"[tiab] OR "intellectually disabled"[tiab] OR "mental retardation"[tiab] OR "intellectual developmental disorder"[tiab] OR "global developmental delay"[tiab] OR "specific learning disorder"[tiab] OR "specific learning disorders"[tiab] OR "learning disability"[tiab] OR "learning disabilities"[tiab] OR dyslexia[tiab] OR dyscalculia[tiab] OR acalculia[tiab] OR "reading disorder"[tiab] OR "mathematics disorder"[tiab] OR "disorder of written expression"[tiab] OR "motor disorder"[tiab] OR "motor disorders"[tiab] OR "developmental coordination disorder"[tiab] OR "developmental coordination disorders"[tiab] OR "motor skills disorder"[tiab] OR "stereotypic movement disorder"[tiab] OR "stereotypic movement disorders"[tiab] OR "communication disorder"[tiab] OR "communication disorders"[tiab] OR "language disorder"[tiab] OR "phonological disorder"[tiab] OR "speech sound disorder"[tiab] OR stuttering[tiab] OR "childhood-onset fluency disorder"[tiab] OR "tic disorder"[tiab] OR "tic disorders"[tiab] OR "tourette's syndrome"[tiab] OR "tourettes disease"[tiab] OR "down syndrome"[tiab] OR "trisomy 21"[tiab] OR "fragile x syndrome"[tiab] OR "martin-bell syndrome"[tiab] OR "williams syndrome"[tiab] OR "williams beuren syndrome"[tiab] OR "prader willi syndrome"[tiab] OR "angelman syndrome"[tiab] OR "rett syndrome"[tiab] OR "turners syndrome"[tiab] OR "smith-magenis syndrome"[tiab] OR "tuberous sclerosis"[tiab] OR "digeorge syndrome"[tiab] OR "velocardiofacial syndrome"[tiab] OR "22q11 deletion syndrome"[tiab] OR "fetal alcohol spectrum disorder"[tiab] OR "fetal alcohol spectrum disorders"[tiab] OR FASD[tiab] OR "cerebral palsy"[tiab] ) | 402,920 |
|  | 2 | ( "Physical activity"[Mesh] OR "Exercise"[Mesh] OR "physical activit*"[tiab] OR "physical fitness"[tiab] OR "physical exercise"[tiab] OR "physical education"[tiab] OR "leisure activit*"[tiab] OR "motor activit*"[tiab] OR "sport*"[tiab] OR "sport* participation"[tiab] OR "fitness"[tiab] OR "cardiovascular fitness"[tiab] OR "athletic*"[tiab] OR "exercis*"[tiab] OR "acute exercise"[tiab] OR "chronic exercise"[tiab] OR "healthy exercise"[tiab] OR "yoga"[tiab] OR "pilates"[tiab] OR "aerobic exercise"[tiab] OR "aerobic"[tiab] OR "aerobic training"[tiab] OR "resistance exercise"[tiab] OR "anaerobic exercise"[tiab] OR "intervention"[tiab] OR "baseball"[tiab] OR "football"[tiab] OR "soccer"[tiab] OR "basketball"[tiab] OR "racket sports"[tiab] OR "racquet sports"[tiab] OR "badminton"[tiab] OR "tennis"[tiab] OR "cricket"[tiab] OR "track and field"[tiab] OR "running"[tiab] OR "sprint"[tiab] OR "long distance running"[tiab] OR "jump"[tiab] OR "bike"[tiab] OR "bicycling"[tiab] OR "boxing"[tiab] OR "taekwondo"[tiab] OR "judo"[tiab] OR "golf"[tiab] OR "gymnastics"[tiab] OR "hockey"[tiab] OR "climbing"[tiab] OR "hiking"[tiab] OR "Tai Ji"[tiab] OR "martial arts"[tiab] OR "swimming"[tiab] OR "aquatic exercise"[tiab] OR "aquatic therapy"[tiab] OR "diving"[tiab] OR "skating"[tiab] OR "snow sports"[tiab] OR "skiing"[tiab] OR "sports for persons with disabilities"[tiab] OR "youth sports"[tiab] OR "wrestling"[tiab] OR "weight lifting"[tiab] OR "game*"[tiab] OR "virtual sports game"[tiab] OR "recreation*"[tiab] OR "motor skills"[tiab] OR "motor intervention"[tiab] OR "functional exercise"[tiab] OR "motor learning"[tiab] OR "behaviour skills training"[tiab] OR "task specific training"[tiab] OR "strength training"[tiab] OR "horse riding"[tiab] OR "equestrian"[tiab] OR "equine-assisted therapy"[tiab] OR "hippotherapy"[tiab] OR "trampoline"[tiab] OR "snowshoeing"[tiab] OR "skating"[tiab] OR "exergaming"[tiab] OR "skateboarding"[tiab] OR "dance"[tiab] OR "walking"[tiab] OR "treadmill"[tiab] OR "karate"[tiab] ) | 2,102,766 |
|  | 3 | ( "Control Groups"[Mesh] OR "Placebos"[Mesh] OR "Standard of Care"[Mesh] OR "Waiting Lists"[Mesh] OR "control group*"[All Fields] OR "comparison group*"[All Fields] OR comparator*[All Fields] OR controlled[All Fields] OR "controlled trial"[All Fields] OR "between-group*"[All Fields] OR "between group*"[All Fields] OR "parallel group*"[All Fields] OR "group design"[All Fields] OR "active control"[All Fields] OR "attention control"[All Fields] OR "contact control"[All Fields] OR "minimal contact"[All Fields] OR "education only"[All Fields] OR "information only"[All Fields] OR "no-contact control"[All Fields] OR "no treatment"[All Fields] OR "no-treatment control"[All Fields] OR "delayed treatment"[All Fields] OR "delayed intervention"[All Fields] OR waitlist*[All Fields] OR "wait-list"[All Fields] OR "waiting list"[All Fields] OR WL[All Fields] OR WLC[All Fields] OR "waitlist control"[All Fields] OR placebo*[All Fields] OR "placebo control*"[All Fields] OR sham[All Fields] OR "sham control*"[All Fields] OR "sham training"[All Fields] OR "sham stimulation"[All Fields] OR "dummy treatment"[All Fields] OR "usual care"[All Fields] OR UC[All Fields] OR "care as usual"[All Fields] OR CAU[All Fields] OR "treatment as usual"[All Fields] OR TAU[All Fields] OR "standard of care"[All Fields] OR "standard care"[All Fields] OR "routine care"[All Fields] OR "usual practice"[All Fields] OR "standard practice"[All Fields] OR "services as usual"[All Fields] OR SAU[All Fields] OR "business as usual"[All Fields] OR BAU[All Fields] OR "enhanced usual care"[All Fields] OR EUC[All Fields] OR "optimized usual care"[All Fields] OR OUC[All Fields] ) | 2,825,349 |
|  | 4 | ( "Randomized Controlled Trials"[Mesh] OR random*[All Fields] OR randomi?ed[All Fields] OR randomi?ation[All Fields] OR "random allocation"[All Fields] OR "random assignment"[All Fields] OR "randomly assigned"[All Fields] OR "randomly allocated"[All Fields] OR RCT[All Fields] OR RCTs[All Fields] OR "randomized controlled trial"[All Fields] OR "randomised controlled trial"[All Fields] OR "randomized trial"[All Fields] OR "randomised trial"[All Fields] OR "randomized clinical trial"[All Fields] OR "randomised clinical trial"[All Fields] OR "clinical trial, randomized"[All Fields] OR "trial, randomized"[All Fields] ) | 1,973,915 |
|  | 1 AND 2 AND 3 AND 4 | | 18,209 |
| Web of Science | 1 | TS = ("neurodevelopmental disorder*" OR "developmental disorder*" OR "autism spectrum disorder*" OR "ASD" OR "autism" OR "asperger*" OR "Pervasive developmental Disorder*" OR "childhood disintegrative disorder" OR "attention deficit hyperactivity disorder*" OR "ADHD" OR "attention deficit disorder*" OR "ADD" OR "attention deficit and disruptive behavior disorder*" OR "intellectual disabilit*" OR "intellectually disabled" OR "mental retardation" OR "intellectual developmental disorder" OR "global developmental delay" OR "specific learning disorder*" OR "learning disabilit*" OR "dyslexia" OR "dyscalculia" OR "Acalculia" OR "reading disorder" OR "mathematics disorder" OR "disorder of written expression" OR "motor disorder*" OR "developmental coordination disorder*" OR "motor skills disorder" OR "stereotypic movement disorder*" OR "communication disorder*" OR "language disorder" OR "phonological disorder" OR "speech sound disorder" OR "stuttering" OR "childhood-onset fluency disorder" OR "tic disorder*" OR "tourette's syndrome" OR "Tourettes disease" OR "Down syndrome" OR "Trisomy 21" OR "Fragile X Syndrome" OR "Martin-Bell syndrome" OR "Williams syndrome" OR "williams beuren syndrome" OR "Prader Willi syndrome" OR "Angelman syndrome" OR "Rett Syndrome" OR "Turners Syndrome" OR "Smith-Magenis syndrome" OR "Tuberous sclerosis" OR "DiGeorge syndrome" OR "velocardiofacial syndrome" OR "22q11 deletion syndrome" OR "Fetal Alcohol Spectrum Disorder*" OR "FASD" OR "cerebral palsy") | 1,262,048 |
|  | 2 | TS = ("Physical activity" OR "Exercise" OR "physical activit*" OR "physical fitness" OR "physical exercise" OR "physical education" OR "leisure activit*" OR "motor activit*" OR "sport*" OR "sport* participation" OR "fitness" OR "cardiovascular fitness" OR "athletic*" OR "exercis*" OR "acute exercise" OR "chronic exercise" OR "healthy exercise" OR "yoga" OR "pilates" OR "aerobic exercise" OR "aerobic" OR "aerobic training" OR "resistance exercise" OR "anaerobic exercise" OR "intervention" OR "baseball" OR "football" OR "soccer" OR "basketball" OR "racket sports" OR "racquet sports" OR "badminton" OR "tennis" OR "cricket" OR "track and field" OR "running" OR "sprint" OR "long distance running" OR "jump" OR "bike" OR "bicycling" OR "boxing" OR "taekwondo" OR "judo" OR "golf" OR "gymnastics" OR "hockey" OR "climbing" OR "hiking" OR "Tai Ji" OR "martial arts" OR "swimming" OR "aquatic exercise" OR "aquatic therapy" OR "diving" OR "skating" OR "snow sports" OR "skiing" OR "sports for persons with disabilities" OR "youth sports" OR "wrestling" OR "weight lifting" OR "game*" OR "virtual sports game" OR "recreation*" OR "motor skills" OR "motor intervention" OR "functional exercise" OR "motor learning" OR "behaviour skills training" OR "task specific training" OR "strength training" OR "horse riding" OR "equestrian" OR "equine-assisted therapy" OR "hippotherapy" OR "trampoline" OR "snowshoeing" OR "skating" OR "exergaming" OR "skateboarding" OR "dance" OR "walking" OR "treadmill" OR "karate") | 6,762,334 |
|  | 3 | TS = ("Control Groups" OR "Placebos" OR "Standard of Care" OR "Waiting Lists" OR "control group*" OR "comparison group*" OR comparator* OR controlled OR "controlled trial" OR "between-group*" OR "between group*" OR "parallel group*" OR "group design" OR "active control" OR "attention control" OR "contact control" OR "minimal contact" OR "education only" OR "information only" OR "no-contact control" OR "no treatment" OR "no-treatment control" OR "delayed treatment" OR "delayed intervention" OR waitlist* OR "wait-list" OR "waiting list" OR WL OR WLC OR "waitlist control" OR placebo* OR "placebo control*" OR sham OR "sham control*" OR "sham training" OR "sham stimulation" OR "dummy treatment" OR "usual care" OR UC OR "care as usual" OR CAU OR "treatment as usual" OR TAU OR "standard of care" OR "standard care" OR "routine care" OR "usual practice" OR "standard practice" OR "services as usual" OR SAU OR "business as usual" OR BAU OR "enhanced usual care" OR EUC OR "optimized usual care" OR OUC) | 18,548,606 |
|  | 4 | TS = ("Randomized Controlled Trials" OR random* OR randomi?ed OR randomi?ation OR "random allocation" OR "random assignment" OR "randomly assigned" OR "randomly allocated" OR RCT OR RCTs OR "randomized controlled trial" OR "randomised controlled trial" OR "randomized trial" OR "randomised trial" OR "randomized clinical trial" OR "randomised clinical trial" OR "clinical trial, randomized" OR "trial, randomized") | 3,907,844 |
|  | 1 AND 2 AND 3 AND 4 | | 38,811 |
| EBSCO | 1 | S1  (MH "Neurodevelopmental Disorders+")  OR  TX (  "neurodevelopmental disorder*" OR "developmental disorder*" OR "autism spectrum disorder*" OR ASD OR autism OR asperger*  OR "Pervasive developmental Disorder*" OR "childhood disintegrative disorder"  OR "attention deficit hyperactivity disorder*" OR ADHD OR "attention deficit disorder*" OR ADD  OR "attention deficit and disruptive behavior disorder*"  OR "intellectual disabilit*" OR "intellectually disabled" OR "mental retardation" OR "intellectual developmental disorder"  OR "global developmental delay"  OR "specific learning disorder*" OR "learning disabilit*" OR dyslexia OR dyscalculia OR Acalculia  OR "reading disorder" OR "mathematics disorder" OR "disorder of written expression"  OR "motor disorder*" OR "developmental coordination disorder*" OR "motor skills disorder" OR "stereotypic movement disorder*"  OR "communication disorder*" OR "language disorder" OR "phonological disorder" OR "speech sound disorder"  OR stuttering OR "childhood-onset fluency disorder"  OR "tic disorder*" OR "tourette's syndrome" OR "Tourettes disease"  OR "Down syndrome" OR "Trisomy 21"  OR "Fragile X Syndrome" OR "Martin-Bell syndrome"  OR "Williams syndrome" OR "williams beuren syndrome"  OR "Prader Willi syndrome" OR "Angelman syndrome"  OR "Rett Syndrome" OR "Turners Syndrome" OR "Smith-Magenis syndrome"  OR "Tuberous sclerosis"  OR "DiGeorge syndrome" OR velocardiofacial syndrome OR "22q11 deletion syndrome"  OR "Fetal Alcohol Spectrum Disorder*" OR FASD  OR "cerebral palsy"  ) | 983,637 |
|  | 2 | S2  (MH "Physical activity" OR MH "Exercise")  OR  TX (  "physical activit*" OR "physical fitness" OR "physical exercise" OR "physical education" OR "leisure activit*"  OR "motor activit*" OR sport* OR "sport* participation" OR fitness OR "cardiovascular fitness" OR athletic*  OR exercis* OR "acute exercise" OR "chronic exercise" OR "healthy exercise" OR yoga OR pilates  OR "aerobic exercise" OR aerobic OR "aerobic training" OR "resistance exercise" OR "anaerobic exercise"  OR intervention  OR baseball OR football OR soccer OR basketball OR "racket sports" OR "racquet sports" OR badminton OR tennis OR cricket  OR "track and field" OR running OR sprint OR "long distance running" OR jump  OR bike OR bicycling OR boxing OR taekwondo OR judo OR golf OR gymnastics OR hockey OR climbing OR hiking  OR "Tai Ji" OR "martial arts"  OR swimming OR "aquatic exercise" OR "aquatic therapy" OR diving OR skating OR "snow sports" OR skiing  OR "sports for persons with disabilities" OR "youth sports"  OR wrestling OR "weight lifting"  OR game* OR "virtual sports game" OR recreation*  OR "motor skills" OR "motor intervention" OR "functional exercise" OR "motor learning"  OR "behaviour skills training" OR "task specific training" OR "strength training"  OR "horse riding" OR equestrian OR "equine-assisted therapy" OR hippotherapy OR trampoline OR snowshoeing  OR exergaming OR skateboarding OR dance OR walking OR treadmill OR karate  ) | 5,658,108 |
|  | 3 | S3  (MH "Control Groups" OR MH "Placebos" OR MH "Standard of Care" OR MH "Waiting Lists")  OR  TX (  "control group*" OR "comparison group*" OR comparator* OR controlled OR "controlled trial"  OR "between-group*" OR "between group*" OR "parallel group*" OR "group design"  OR "active control" OR "attention control" OR "contact control" OR "minimal contact"  OR "education only" OR "information only" OR "no-contact control" OR "no treatment" OR "no-treatment control"  OR "delayed treatment" OR "delayed intervention"  OR waitlist* OR "wait-list" OR "waiting list" OR WL OR WLC OR "waitlist control"  OR placebo* OR "placebo control*" OR sham OR "sham control*" OR "sham training" OR "sham stimulation" OR "dummy treatment"  OR "usual care" OR UC OR "care as usual" OR CAU OR "treatment as usual" OR TAU  OR "standard of care" OR "standard care" OR "routine care" OR "usual practice" OR "standard practice"  OR "services as usual" OR SAU OR "business as usual" OR BAU  OR "enhanced usual care" OR EUC OR "optimized usual care" OR OUC  ) | 13,748,658 |
|  | 4 | S4  (MH "Randomized Controlled Trials")  OR  TX (  random* OR randomi?ed OR randomi?ation OR "random allocation" OR "random assignment"  OR "randomly assigned" OR "randomly allocated"  OR RCT OR RCTs  OR "randomized controlled trial" OR "randomised controlled trial"  OR "randomized trial" OR "randomised trial"  OR "randomized clinical trial" OR "randomised clinical trial"  OR "clinical trial, randomized" OR "trial, randomized"  ) | 2,141,843 |
|  | S5 = S1 AND S2 AND (S4 OR S3) | | 34,742 |
| Scopus | 1 | (  TITLE-ABS-KEY(  "neurodevelopmental disorder*" OR "developmental disorder*" OR "autism spectrum disorder*" OR ASD OR autism OR asperger*  OR "pervasive developmental disorder*" OR "childhood disintegrative disorder"  OR "attention deficit hyperactivity disorder*" OR ADHD OR "attention deficit disorder*" OR ADD  OR "attention deficit and disruptive behavior disorder*"  OR "intellectual disabilit*" OR "intellectually disabled" OR "mental retardation" OR "intellectual developmental disorder"  OR "global developmental delay"  OR "specific learning disorder*" OR "learning disabilit*" OR dyslexia OR dyscalculia OR Acalculia  OR "reading disorder" OR "mathematics disorder" OR "disorder of written expression"  OR "motor disorder*" OR "developmental coordination disorder*" OR "motor skills disorder" OR "stereotypic movement disorder*"  OR "communication disorder*" OR "language disorder" OR "phonological disorder" OR "speech sound disorder"  OR stuttering OR "childhood-onset fluency disorder"  OR "tic disorder*" OR "tourette's syndrome" OR "tourettes disease"  OR "down syndrome" OR "trisomy 21"  OR "fragile x syndrome" OR "martin-bell syndrome"  OR "williams syndrome" OR "williams beuren syndrome"  OR "prader willi syndrome" OR "angelman syndrome"  OR "rett syndrome" OR "turners syndrome" OR "smith-magenis syndrome"  OR "tuberous sclerosis"  OR "digeorge syndrome" OR velocardiofacial syndrome OR "22q11 deletion syndrome"  OR "fetal alcohol spectrum disorder*" OR FASD  OR "cerebral palsy"  )  )  AND  (  TITLE-ABS-KEY(  "physical activit*" OR "physical fitness" OR "physical exercise" OR "physical education" OR "leisure activit*"  OR "motor activit*" OR sport* OR "sport* participation" OR fitness OR "cardiovascular fitness" OR athletic*  OR exercis* OR "acute exercise" OR "chronic exercise" OR "healthy exercise" OR yoga OR pilates  OR "aerobic exercise" OR aerobic OR "aerobic training" OR "resistance exercise" OR "anaerobic exercise"  OR intervention  OR baseball OR football OR soccer OR basketball OR "racket sports" OR "racquet sports" OR badminton OR tennis OR cricket  OR "track and field" OR running OR sprint OR "long distance running" OR jump  OR bike OR bicycling OR boxing OR taekwondo OR judo OR golf OR gymnastics OR hockey OR climbing OR hiking  OR "tai ji" OR "martial arts"  OR swimming OR "aquatic exercise" OR "aquatic therapy" OR diving OR skating OR "snow sports" OR skiing  OR "sports for persons with disabilities" OR "youth sports"  OR wrestling OR "weight lifting"  OR game* OR "virtual sports game" OR recreation*  OR "motor skills" OR "motor intervention" OR "functional exercise" OR "motor learning"  OR "behaviour skills training" OR "task specific training" OR "strength training"  OR "horse riding" OR equestrian OR "equine-assisted therapy" OR hippotherapy OR trampoline OR snowshoeing  OR exergaming OR skateboarding OR dance OR walking OR treadmill OR karate  )  )  AND  (  TITLE-ABS-KEY(  random* OR randomi?ed OR randomi?ation OR "random allocation" OR "random assignment"  OR "randomly assigned" OR "randomly allocated" OR RCT OR RCTs  OR "randomized controlled trial" OR "randomised controlled trial"  OR "randomized trial" OR "randomised trial"  OR "randomized clinical trial" OR "randomised clinical trial"  OR "clinical trial, randomized" OR "trial, randomized"  )  AND  TITLE-ABS-KEY(  "control group*" OR "comparison group*" OR comparator* OR controlled OR "controlled trial"  OR "between-group*" OR "between group*" OR "parallel group*" OR "group design"  OR "active control" OR "attention control" OR "contact control" OR "minimal contact"  OR "education only" OR "information only" OR "no-contact control" OR "no treatment" OR "no-treatment control"  OR "delayed treatment" OR "delayed intervention"  OR waitlist* OR "wait-list" OR "waiting list" OR WL OR WLC OR "waitlist control"  OR placebo* OR "placebo control*" OR sham OR "sham control*" OR "sham training" OR "sham stimulation" OR "dummy treatment"  OR "usual care" OR UC OR "care as usual" OR CAU OR "treatment as usual" OR TAU  OR "standard of care" OR "standard care" OR "routine care" OR "usual practice" OR "standard practice"  OR "services as usual" OR SAU OR "business as usual" OR BAU  OR "enhanced usual care" OR EUC OR "optimized usual care" OR OUC  )  ) | 17,021 |
| Cochrane Library | 1 | MeSH descriptor: [Neurodevelopmental Disorders] explode all trees | 11954 |
|  | 2 | ((neurodevelopmental NEXT disorder*):ti,ab,kw OR  (developmental NEXT disorder*):ti,ab,kw OR  (autism NEXT spectrum NEXT disorder*):ti,ab,kw OR  ASD:ti,ab,kw OR  autism:ti,ab,kw OR  asperger*:ti,ab,kw OR  (pervasive NEXT developmental NEXT disorder*):ti,ab,kw OR  (childhood NEXT disintegrative NEXT disorder):ti,ab,kw OR  (attention NEXT deficit NEXT hyperactivity NEXT disorder*):ti,ab,kw OR  ADHD:ti,ab,kw OR  (attention NEXT deficit NEXT disorder*):ti,ab,kw OR  ADD:ti,ab,kw OR  (attention NEXT deficit NEXT disruptive NEXT behavior NEXT disorder*):ti,ab,kw OR  (intellectual NEXT disabilit*):ti,ab,kw OR  (intellectually NEXT disabled):ti,ab,kw OR  (mental NEXT retardation):ti,ab,kw OR  (intellectual NEXT developmental NEXT disorder):ti,ab,kw OR  (global NEXT developmental NEXT delay):ti,ab,kw OR  (specific NEXT learning NEXT disorder*):ti,ab,kw OR  (learning NEXT disabilit*):ti,ab,kw OR  dyslexia:ti,ab,kw OR  dyscalculia:ti,ab,kw OR  Acalculia:ti,ab,kw OR  (reading NEXT disorder):ti,ab,kw OR  (mathematics NEXT disorder):ti,ab,kw OR  (disorder NEXT of NEXT written NEXT expression):ti,ab,kw OR  (motor NEXT disorder*):ti,ab,kw OR  (developmental NEXT coordination NEXT disorder*):ti,ab,kw OR  (motor NEXT skills NEXT disorder):ti,ab,kw OR  (stereotypic NEXT movement NEXT disorder*):ti,ab,kw OR  (communication NEXT disorder*):ti,ab,kw OR  (language NEXT disorder):ti,ab,kw OR  (phonological NEXT disorder):ti,ab,kw OR  (speech NEXT sound NEXT disorder):ti,ab,kw OR  stuttering:ti,ab,kw OR  (childhood-onset NEXT fluency NEXT disorder):ti,ab,kw OR  (tic NEXT disorder*):ti,ab,kw OR  (tourette's NEXT syndrome):ti,ab,kw OR  (Tourettes NEXT disease):ti,ab,kw OR  (Down NEXT syndrome):ti,ab,kw OR  (Trisomy NEXT 21):ti,ab,kw OR  (Fragile NEXT X NEXT Syndrome):ti,ab,kw OR  (Martin-Bell NEXT syndrome):ti,ab,kw OR  (Williams NEXT syndrome):ti,ab,kw OR  (williams NEXT beuren NEXT syndrome):ti,ab,kw OR  (Prader NEXT Willi NEXT syndrome):ti,ab,kw OR  (Angelman NEXT syndrome):ti,ab,kw OR  (Rett NEXT Syndrome):ti,ab,kw OR  (Turners NEXT Syndrome):ti,ab,kw OR  (Smith-Magenis NEXT syndrome):ti,ab,kw OR  (Tuberous NEXT sclerosis):ti,ab,kw OR  (DiGeorge NEXT syndrome):ti,ab,kw OR  (velocardiofacial NEXT syndrome):ti,ab,kw OR  (22q11 NEXT deletion NEXT syndrome):ti,ab,kw OR  (Fetal NEXT Alcohol NEXT Spectrum NEXT Disorder*):ti,ab,kw OR  FASD:ti,ab,kw OR  (cerebral NEXT palsy):ti,ab,kw) | 48637 |
|  | 3 | MeSH descriptor: [Exercise] explode all trees | 42085 |
|  | 4 | ((physical NEXT activit*):ti,ab,kw OR  "physical fitness":ti,ab,kw OR  "physical exercise":ti,ab,kw OR  "physical education":ti,ab,kw OR  (leisure NEXT activit*):ti,ab,kw OR  (motor NEXT activit*):ti,ab,kw OR  sport*:ti,ab,kw OR  (sport* NEXT participation):ti,ab,kw OR  fitness:ti,ab,kw OR  "cardiovascular fitness":ti,ab,kw OR  athletic*:ti,ab,kw OR  exercis*:ti,ab,kw OR  "acute exercise":ti,ab,kw OR  "chronic exercise":ti,ab,kw OR  "healthy exercise":ti,ab,kw OR  yoga:ti,ab,kw OR  pilates:ti,ab,kw OR  "aerobic exercise":ti,ab,kw OR  aerobic:ti,ab,kw OR  "aerobic training":ti,ab,kw OR  "resistance exercise":ti,ab,kw OR  "anaerobic exercise":ti,ab,kw OR  intervention:ti,ab,kw OR  baseball:ti,ab,kw OR  football:ti,ab,kw OR  soccer:ti,ab,kw OR  basketball:ti,ab,kw OR  "racket sports":ti,ab,kw OR  "racquet sports":ti,ab,kw OR  badminton:ti,ab,kw OR  tennis:ti,ab,kw OR  cricket:ti,ab,kw OR  "track and field":ti,ab,kw OR  running:ti,ab,kw OR  sprint:ti,ab,kw OR  "long distance running":ti,ab,kw OR  jump:ti,ab,kw OR  bike:ti,ab,kw OR  bicycling:ti,ab,kw OR  boxing:ti,ab,kw OR  taekwondo:ti,ab,kw OR  judo:ti,ab,kw OR  golf:ti,ab,kw OR  gymnastics:ti,ab,kw OR  hockey:ti,ab,kw OR  climbing:ti,ab,kw OR  hiking:ti,ab,kw OR  "Tai Ji":ti,ab,kw OR  "martial arts":ti,ab,kw OR  swimming:ti,ab,kw OR  "aquatic exercise":ti,ab,kw OR  "aquatic therapy":ti,ab,kw OR  diving:ti,ab,kw OR  skating:ti,ab,kw OR  "snow sports":ti,ab,kw OR  skiing:ti,ab,kw OR  "sports for persons with disabilities":ti,ab,kw OR  "youth sports":ti,ab,kw OR  wrestling:ti,ab,kw OR  "weight lifting":ti,ab,kw OR  game*:ti,ab,kw OR  (virtual NEXT sports NEXT game):ti,ab,kw OR  recreation*:ti,ab,kw OR  "motor skills":ti,ab,kw OR  "motor intervention":ti,ab,kw OR  "functional exercise":ti,ab,kw OR  "motor learning":ti,ab,kw OR  "behaviour skills training":ti,ab,kw OR  "task specific training":ti,ab,kw OR  "strength training":ti,ab,kw OR  "horse riding":ti,ab,kw OR  equestrian:ti,ab,kw OR  "equine-assisted therapy":ti,ab,kw OR  hippotherapy:ti,ab,kw OR  trampoline:ti,ab,kw OR  snowshoeing:ti,ab,kw OR  skating:ti,ab,kw OR  exergaming:ti,ab,kw OR  skateboarding:ti,ab,kw OR  dance:ti,ab,kw OR  walking:ti,ab,kw OR  treadmill:ti,ab,kw OR  karate:ti,ab,kw) | 785994 |
|  | 5 | MeSH descriptor: [Control Groups] explode all trees | 476 |
|  | 6 | ((control NEXT group*):ti,ab,kw OR  (comparison NEXT group*):ti,ab,kw OR  comparator*:ti,ab,kw OR  controlled:ti,ab,kw OR  "controlled trial":ti,ab,kw OR  (between-group*):ti,ab,kw OR  (between NEXT group*):ti,ab,kw OR  (parallel NEXT group*):ti,ab,kw OR  "group design":ti,ab,kw OR  "active control":ti,ab,kw OR  "attention control":ti,ab,kw OR  "contact control":ti,ab,kw OR  "minimal contact":ti,ab,kw OR  "education only":ti,ab,kw OR  "information only":ti,ab,kw OR  "no-contact control":ti,ab,kw OR  "no treatment":ti,ab,kw OR  "no-treatment control":ti,ab,kw OR  "delayed treatment":ti,ab,kw OR  "delayed intervention":ti,ab,kw OR  waitlist*:ti,ab,kw OR  "wait-list":ti,ab,kw OR  "waiting list":ti,ab,kw OR  WL:ti,ab,kw OR  WLC:ti,ab,kw OR  "waitlist control":ti,ab,kw OR  placebo*:ti,ab,kw OR  (placebo NEXT control*):ti,ab,kw OR  sham:ti,ab,kw OR  (sham NEXT control*):ti,ab,kw OR  "sham training":ti,ab,kw OR  "sham stimulation":ti,ab,kw OR  "dummy treatment":ti,ab,kw OR  "usual care":ti,ab,kw OR  UC:ti,ab,kw OR  "care as usual":ti,ab,kw OR  CAU:ti,ab,kw OR  "treatment as usual":ti,ab,kw OR  TAU:ti,ab,kw OR  "standard of care":ti,ab,kw OR  "standard care":ti,ab,kw OR  "routine care":ti,ab,kw OR  "usual practice":ti,ab,kw OR  "standard practice":ti,ab,kw OR  "services as usual":ti,ab,kw OR  SAU:ti,ab,kw OR  "business as usual":ti,ab,kw OR  BAU:ti,ab,kw OR  "enhanced usual care":ti,ab,kw OR  EUC:ti,ab,kw OR  "optimized usual care":ti,ab,kw OR  OUC:ti,ab,kw) | 1445027 |
|  | 7 | MeSH descriptor: [Randomized Controlled Trial] explode all trees | 34 |
|  | 8 | (random*:ti,ab,kw OR  randomi?ed:ti,ab,kw OR  randomi?ation:ti,ab,kw OR  (random NEXT allocation):ti,ab,kw OR  (random NEXT assignment):ti,ab,kw OR  (randomly NEXT assigned):ti,ab,kw OR  (randomly NEXT allocated):ti,ab,kw OR  RCT:ti,ab,kw OR  RCTs:ti,ab,kw OR  (randomized NEXT controlled NEXT trial):ti,ab,kw OR  (randomised NEXT controlled NEXT trial):ti,ab,kw OR  (randomized NEXT trial):ti,ab,kw OR  (randomised NEXT trial):ti,ab,kw OR  (randomized NEXT clinical NEXT trial):ti,ab,kw OR  (randomised NEXT clinical NEXT trial):ti,ab,kw OR  (clinical NEXT trial NEXT randomized):ti,ab,kw OR  (trial NEXT randomized):ti,ab,kw) | 1464201 |
|  | S9 = (S1 OR S2) AND (S3 OR S4) AND (S5 OR S6) AND (S7 OR S8) | | 13505 |

**Supplementary Table 4:** Inclusion and exclusion criteria in PICOS frame.

| **PICOS Framework** | | | |
| --- | --- | --- | --- |
| **PICOS** | **Inclusion criteria** | | **Exclusion criteria** |
| Population | • Individuals with a confirmed neurodevelopmental disorder (NDD) diagnosis.  • Diagnosis confirmed via (i) clinician assessment using an authoritative nosological system (e.g., DSM-5) and/or (ii) validated standardized diagnostic instruments meeting established thresholds (e.g., ADOS-2).  • Mixed-sample studies included only if NDD subgroup data are separately extractable or the NDD proportion meets a prespecified threshold (e.g., ≥80%).  • No restrictions on age, sex/gender, geographic region, or intervention setting. | | • Participants without a clearly defined NDD diagnosis or without evidence of meeting standardized diagnostic criteria/thresholds.  • Mixed-sample studies excluded if NDD-specific outcomes are not independently extractable and the NDD proportion is below the prespecified threshold (e.g., <80%).  • Studies excluded if diagnosis is based solely on non-validated screening tools or self-report without clinical confirmation or standardized diagnostic validation. |
| Intervention | • Physical activity interventions (PAIs) with ≥50% of session time devoted to participant-performed active exercise.  • Eligible modalities: resistance/strength, aerobic, neuromuscular (sensorimotor/postural control; balance/coordination), or combinations.  • Passive adjuncts permitted only if the ≥50% active criterion is maintained; any usual care must be described and equivalent across arms. | | • Multimodal interventions (e.g., pharmacotherapy, surgery, electrotherapy) in which exercise effects or exercise-related adverse events cannot be isolated.  • Predominantly passive or static protocols with minimal active muscle work (e.g., whole-body vibration).  • Stretching and/or ROM-only programs without systematic resistance, aerobic, or neuromuscular training.  • Primarily behavioral or habit-training programs not driven by physical activity (e.g., bladder training). |
| Comparison | Meta-  analysis | • Usual care or wait-list control with no physical activity/exercise component.  • Attention control, education/psychoeducation, placebo, or nutritional supplementation comparators, provided they include no structured or therapeutic physical activity.  • Comparator protocols explicitly described as non-exercise to isolate physical activity vs non-physical activity effects. | • Any comparator with exercise-related content (e.g., planned training, therapeutic exercise, or stretching/flexibility programs).  • Comparators primarily involving pharmacotherapy, surgical/invasive procedures, electrotherapy, or other physical-agent modalities. |
|  | Critical Appraisal | • Any comparator condition, including exercise/physical activity, usual care/wait-list, attention control, health education/psychoeducation (e.g., “back school”), placebo, and nutraceuticals. | • Comparators primarily involving pharmacotherapy, surgical/invasive procedures, electrotherapy, or other physical-agent modalities. |
| Outcome | Primary outcome: adverse events occurrence during intervention and follow-up, comparing relative risk (RR) between intervention and control groups. Secondary outcomes: adverse events reporting rate; withdrawals/dropouts and reasons (including reasons suggestive of adverse events/adverse effects); adverse event types; and quality of adverse event monitoring. | | |
| Study design | • Randomized controlled trials (RCTs) with explicit random allocation to ≥2 groups and outcome assessment at baseline and post-intervention and/or follow-up.  • Eligible designs: individually randomized parallel, cluster-randomized, randomized crossover, stepped-wedge, and other randomized variants (randomized allocation/order required). | | • Nonrandom or quasi-experimental allocation (e.g., order/date/time/site, clinician or participant preference).  • Studies lacking a clearly described randomization procedure sufficient to verify truly random allocation. |

4. **ASReview—Rationale, Architecture, and Study-Specific**

**3.1 ASReview in brief**

ASReview is a free, open-source platform that uses pool-based active learning to prioritise citations during title and abstract screening in evidence syntheses.^1^ Rather than screening records in an arbitrary or database-defined order, the system iteratively trains a supervised model on the reviewer’s relevance judgements and re-ranks the remaining pool to present the most promising records first.^2^ The software generates a reproducible audit trail, supports interactive LAB-mode screening and includes simulation and exploration modes for benchmarking and training.^3^ In large screening tasks, this approach can substantially reduce manual workload while maintaining high recall of eligible studies.^4^

At the implementation level, the platform uses a modular pipeline with four independently configurable components: (i) feature extraction (e.g. TF–IDF, Doc2Vec or Sentence-BERT embeddings); (ii) a binary classifier (e.g. multinomial naïve Bayes, logistic regression, linear SVM, random forest or shallow neural networks); (iii) a query strategy that selects the next record to label (e.g. exploitation of the highest predicted relevance, uncertainty sampling of instances near the decision boundary or hybrid explore–exploit rules); and (iv) a class-imbalance remedy (e.g. dynamic resampling) to address the scarcity of relevant studies early in screening.^1^ The system is usually initialised with minimal prior knowledge (at least one relevant and one irrelevant record) to start the learning loop; after each label, the model is refitted and the remaining pool is re-ranked.^2^

For stopping decisions, the software does not prescribe a single universal rule. Current practice relies on data-driven heuristics (most commonly, stopping after a prespecified number of consecutive irrelevant records) and on conservative frameworks such as SAFE, which combine multiple diagnostics to control the risk of halting before most relevant records have been retrieved.^5^ In practice, heuristic thresholds are scaled to dataset size and recall targets, and SAFE provides a principled, conservative template.^6^

Consistent with these design choices, empirical evaluations report substantial workload reductions at high recall. For example, simulations and case studies have documented scenarios in which approximately 95% of eligible studies were identified after screening only about 8–33% of the pool, with the remaining records deprioritised by the model, although exact savings depend on the topic, data quality and model settings.^7-9^

**3.2 How ASReview was used in this review**

After completing the database searches, all records were imported into EndNote (version 20) for automated deduplication, followed by manual verification by an independent reviewer (J.H.). The deduplicated corpus contained 41,852 unique records, which were exported in RIS format and imported into ASReview LAB for screening. To initialise the model, each screener first labelled five relevant and five irrelevant records. ASReview then began the active-learning loop: at each step, the model re-ranked the unscreened pool and presented the highest-ranked record for human judgement; after each decision, it retrained on the expanded labelled set and updated the ranking for the next iteration.

**Model components (pre-specified before screening)**

- **Feature extraction: TF-IDF bag-of-words (unigram and bigram) on titles and abstracts.**
- **Classifier: Support Vector Machine (SVM) (linear, for sparse high-dimensional text features).**
- **Query strategy: maximum-probability exploitation, presenting first the records with the highest estimated probability of relevance.**
- **Class imbalance: Balanced (cost-sensitive learning via class weighting to counter skewed label distributions).**

We selected TF-IDF features with a support vector machine (SVM) classifier and a maximum-probability query strategy as the primary configuration for three methodological reasons. First, TF–IDF produces a sparse, high-dimensional representation that aligns well with linear SVMs, which learn large-margin decision boundaries and tend to generalize well in text classification tasks where informative signals are distributed across many terms.^10^ Second, relative to probabilistic generative baselines, SVMs often achieve strong discriminative performance with limited labeled data—an important consideration in active screening, where early iterations are label-scarce and decision boundaries must be estimated from few positive examples. Third, this approach remains relatively transparent: feature weights can be examined to identify which terms contribute most to the relevance signal, thereby supporting the auditability of screening decisions.^11 12^

Two reviewers (J.H., C.P.) independently screened titles and abstracts in separate, identically configured ASReview projects. Conflicts—defined as any discordant inclusion or exclusion decision—were exported for discussion, and unresolved cases were adjudicated by a third reviewer (X.W.). All labelling logs, settings files and model metadata were archived to ensure reproducibility. ASReview was used strictly as a prioritisation aid; all inclusion and exclusion decisions were made by human reviewers according to prespecified criteria.

To operationalise a conservative stopping point while leveraging dynamic prioritisation, we adopted a consecutive-irrelevant heuristic scaled to corpus size: screening ceased once 600 consecutive records (approximately 2% of the total pool) had been labelled irrelevant with no newly discovered eligible study. Under a maximum-probability query strategy, this threshold indicates that the top tranche of the ranking has been exhausted without yielding additional relevant records, making the remaining tail unlikely to materially affect eligibility. This practice aligns with contemporary usage and complements more formal procedures such as SAFE.^5 6^

**Prioritized screening yield and conservative stopping point**

**Supplementary figure 2.** The curve depicts the cumulative number of eligible studies across the ranked screening order generated by ASReview under a maximum-probability (greedy) query strategy. The trajectory shows a steep early rise (shaded region), followed by a long, low-yield tail. We prespecified a conservative consecutive-irrelevant stopping rule: screening ceased after 600 consecutive records had been labelled irrelevant with no new inclusions (approximately 2% of the corpus), indicating that the high-priority segment of the ranking had been exhausted and that the remaining tail was unlikely to materially change study eligibility. No additional eligible studies were identified beyond this point.

**Cumulative discovery under prioritised active learning versus random ordering**

**Supplementary figure 3.** Cumulative yield of potentially eligible (relevant) records during title–abstract screening. The yellow curve shows screening guided by ASReview LAB using a maximum-probability (greedy) query strategy, whereas the grey curve provides a random-ordering comparator. ASReview concentrated relevant records early in the ranked list: after screening ~20%, ~40% and ~60% of records (≈480, 960 and 1,440 of ~2,400), approximately ~55%, ~90% and >95% of all relevant records had already been identified (final yield ≈600). This corresponds to substantial workload savings (WSS@85≈65% and WSS@95≈45%). The subsequent plateau reflects a long, low-yield tail consistent with our prespecified conservative stopping rule (termination after 600 consecutive irrelevant judgements without new inclusions), and a targeted post hoc sweep of the residual pool did not identify any additional eligible records.

**Gaps Between Consecutive Eligible Records**

**Supplementary figure 4.** Wave plot of gaps between consecutive eligible records during ASReview-guided screening. Each point represents the number of ineligible records encountered between two successive eligible records as a function of the cumulative number of records screened. Across most of the ranked corpus, gaps remained short, indicating a high density of eligible studies. Towards the tail of the ranking, gaps increased steeply and exceeded the prespecified stopping threshold of 600 consecutive irrelevant records, suggesting that the remaining pool consisted predominantly of ineligible records.

5. **Codebook**

**Part I: Basic Study Information**

**A:** Reference ID
**B:** Year of publication

**C:** Country

The country should be coded according to the setting in which the intervention was actually delivered, rather than the institutional affiliation of the first or corresponding author.

**Part II: Participant Characteristics**

**Notes:**

Report all of the following data and, where necessary, combine treatment and control groups using a weighted mean. The combined mean should be calculated as:

Here, mean1 and mean2 represent the means of the treatment and control groups, respectively, and n1 and n2 are their corresponding sample sizes (an Excel tool is provided in the practical coding file).

1. If the study reports a mean for the entire sample, use the reported mean directly.
2. If the study reports the median and range but not the mean, use the Excel tool provided in the practical coding file to estimate the mean.
3. If only the median is reported and no range is given, use the median as the best available estimate of the mean.
4. If data are reported at multiple time points, use the data from the first time point (i.e. at study enrolment). If the first time point is not reported, use data from the earliest available time point.
5. If an item is not reported, enter “NR” to indicate missing data.
6. If group means or sample sizes reported in the text differ from those in the tables, use the values reported in the tables.

**D:** Types of neurodevelopmental disorders

Neurodevelopmental disorders are a class of neuropsychiatric conditions that typically begin in early childhood and are characterized by persistent impairments in cognitive and/or developmental functioning that may continue across the lifespan. According to the DSM-5, these disorders include attention-deficit/hyperactivity disorder (ADHD), autism spectrum disorder (ASD), intellectual developmental disorders (IDD), specific learning disorder (SLD), communication disorders, and motor disorders (e.g., developmental coordination disorder). Guided by our research question and the existing evidence base, we categorized eligible neurodevelopmental disorder populations as **ADHD**, **ASD**, **SLD**, **IDD**, and developmental coordination disorder (**DCD**). Participants with two or more of these diagnoses were classified as having comorbid neurodevelopmental disorders (**comorbid NDD**).

**E:** Actual age at study entry (years, to two decimal places)

If age is reported in months, divide the value by 12 and round to two decimal places (e.g. 18 months = 1.50). If age is reported in decimal years, record the value directly (e.g. 3.25 years = 3.25). If age is reported in a years:months format (e.g. 3 years 6 months), multiply the years by 12, add the months, divide by 12 and round to two decimal places (e.g. 3 years 6 months = (3×12+6)/12 = 3.50). All age data should be recorded in Arabic numerals and rounded to two decimal places.

**F:** Male proportion
Calculate the male proportion by summing the number of male participants across all groups, dividing by the total number of participants in the study and multiplying by 100.

**Part III: Intervention**
Note: If a study compares two interventions with a control group, record data separately for each intervention.

**G:** Type of intervention (with definition)

This variable was used to classify the type of physical activity intervention according to the primary content of the intervention protocol and its training objectives. The operational definitions for each category are provided below.

a) **Aerobic training:** Aerobic training aims primarily to enhance cardiorespiratory endurance, improve metabolic function and promote cardiovascular health. Performed under conditions of adequate oxygen supply, it involves rhythmic, repetitive and continuous dynamic contractions of large muscle groups.^13 14^ Common forms include running, swimming, cycling, brisk walking and fitness training.
b) **Resistance/strengthening training:** An exercise intervention primarily designed to improve skeletal muscle strength, muscular endurance, or power. It typically involves repeated muscle contractions against external resistance or body weight and is systematically prescribed using a specified load and progression (e.g., sets and repetitions). Common examples include resistance training with dumbbells, barbells, resistance bands, or strength-training machines, as well as functional strengthening exercises such as weighted squats, step-ups, bridges, and core strengthening.
c) **Neuromuscular training:** An exercise intervention primarily designed to improve sensorimotor control and functional stability. These interventions typically involve structured training that targets balance, postural control, proprioception, coordination, agility, and functional movement patterns. Common components include static and dynamic balance training; trunk and postural control exercises; proprioceptive and coordination training; agility and change-of-direction drills; and training to improve functional movement quality.
d) **Combined (multicomponent) exercise interventions:** Interventions that systematically integrate two or more exercise types (e.g., aerobic, resistance, and/or neuromuscular) within a single program. Each component represents a substantive and repeatedly implemented element of the intervention, such that the program cannot be readily classified as a single exercise type.

**H:** Intervention setting
(Select the location in which ≥50% of the intervention takes place.)

**a) Home or community:** The intervention is conducted primarily in the home or in community settings. “Community” is defined broadly as any natural setting in which children typically engage in daily activities, excluding clinics, classrooms and the home, such as parks, libraries, playgrounds or early childhood centres. Applicable scenarios include parent-led home interventions, community-based PA programmes, community childcare or social-skills training.

**b) Clinic:** The intervention takes place in clinical settings, including professional facilities that provide services for children, such as rehabilitation centres, hospital intervention rooms, speech therapy rooms or physical training centres. Applicable scenarios include PA programmes led by professional therapists or coaches.

**c) Classroom:** The intervention is conducted primarily within educational institutions, such as general or special education classrooms, preschools or childcare centres. Applicable scenarios include teacher-implemented inclusive PA or behaviour-support strategies and school-based educational intervention plans (e.g. individualised education programmes [IEPs]).

**d) Combination:** Select this category if intervention time is approximately evenly distributed across two or three of the settings above. Applicable scenarios include cases in which each setting accounts for roughly one-third of the total intervention time, or where two settings each account for around 40%, and all participants receive a broadly balanced dose of intervention across multiple locations.

**e) Other:** Select “Other” if the intervention setting is not clearly defined or does not fit any of the categories above, or if intervention settings differ across participant groups (e.g. some participants receive the intervention at home, others in classrooms). Applicable scenarios include unclearly or vaguely described settings, or considerable individual variation that prevents classification into a single category.

**I:** Interventionist type
Select the appropriate interventionist type based on their role and the proportion of intervention time they delivered in the study:

**a) Caregiver:** Select this category if the interventionist is a parent, caregiver or other family member who delivers more than 60% of the intervention time, indicating that family members are the primary implementers.

**b) Clinician:** An intervention was classified as the clinician type when it was delivered primarily by professional therapists or clinical service providers (e.g., rehabilitation therapists, clinicians, psychologists, or researchers) and these providers accounted for >60% of total delivery time. This classification typically applies to interventions that primarily target therapeutic or rehabilitative outcomes and are delivered under professional supervision or within a clinical framework.

**c) Educator/Coach:** An intervention was classified as the educator/coach type when it was delivered primarily by professionals in educational or training settings (e.g., classroom teachers, special education teachers, teaching assistants, school physical education teachers, or sports/fitness coaches who provide structured physical activity training). These professionals served as the primary implementers—typically accounting for >60% of total delivery time—and were not formal members of the research team involved in study design or data collection. This classification most commonly applies to interventions delivered in natural classroom settings, school-based physical education, after-school clubs, or community sport and fitness programs.

**d) Combination:** Select this category if the intervention involves at least two of the following roles—caregivers, clinicians and educators—with each contributing no less than 40% of the intervention time. This may occur, for example, when caregivers and clinicians jointly implement the intervention, or when the study explicitly states that each role provides at least 40% of the intervention dose. For instance, if a study notes that parents deliver 40% of the intervention and clinicians another 40%, this category should be selected.

**e) Computer-based instruction:** Select this category if the primary mode of intervention delivery is via computer or other electronic media, even if a clinician is present during the session. This typically applies to interventions delivered through computer programmes, online platforms or other electronic delivery systems.

**f) Other:** Select this category if the interventionist type does not fall into any of the categories above or if the role is not clearly defined.

**J:** Intervention format (group vs. individual)

**a) Group intervention (Group):** An intervention was classified as group-based when two or more participants with NDD participated simultaneously and the proportion of time during which multiple participants took part in the same exercise program at the same time exceeded 60%. Group-based interventions are typically delivered as small-group classes, classroom activities, group training camps, or sport club/team training. In these formats, multiple participants receive the same or highly similar instruction at the same time and in the same setting (or on the same online platform). Even when individualized adjustments are made within the group (e.g., tailoring difficulty), the intervention is still classified as group-based as long as the primary format involves simultaneous participation by multiple individuals. Examples include a therapist-led six-person exercise group delivered twice per week; whole-class physical activity sessions implemented in general or special education classrooms; and group dance classes or team-based ball-sport training.

**b) Individual intervention (Individual):** An intervention was classified as individual-based when it was delivered primarily on an individual basis and the proportion of time during which participants completed exercise/physical activity independently (or in a one-to-one setting) exceeded 60%. Typical scenarios include one-to-one therapeutic exercise sessions (e.g., individualized training delivered by a therapist to a single participant); individualized home-based exercise prescriptions completed independently (with optional remote follow-up or telephone prompts); and participants completing a prescribed training plan independently in a gym or community setting, even when others are present, provided there are no organized group sessions in which participants follow the same protocol together. Examples include 45-minute one-to-one postural control sessions conducted between a therapist and a child three times per week; individualized home-exercise manuals implemented with parental assistance but without any group sessions among participants; and individualized aerobic training using a treadmill or cycle ergometer, with each session delivered to a single participant.

**c) Combination format (group + individual):** An intervention was classified as a combination format when it included both group-based and individual-based delivery and each format accounted for ≥40% of total intervention delivery time. These interventions typically have a predefined structure that allocates a relatively balanced dose to each delivery mode. Examples include one group training session per week plus multiple prescribed doses of individualized home practice, with the two components contributing approximately equally to total training time; or an intervention delivered one-on-one during the first half and in a group format during the second half, with the two phases similar in overall duration (each ≥40%).

**d) Unclear/other:** An intervention was classified as unclear/other when the description of intervention format was insufficient to determine whether delivery was primarily group-based or individual-based, or when participants’ exposure was highly heterogeneous such that no single primary format could be defined. This category applies when articles provide only general statements (e.g., “participated in program activities” or “performed physical exercise”) without specifying whether delivery involved small-group classes, classroom activities, or individual training; when some participants attended group sessions while others primarily received individual or home-based intervention and the study did not report time proportions across formats, precluding inference about the primary delivery mode; or when delivery was highly flexible (e.g., “arranged according to family/school circumstances”), making it impossible to identify a consistent group or individual format.

**K: Physical Activity Intensity:** Categorise intensity as LMPA (light-to-moderate physical activity), MPA (moderate physical activity), MVPA (moderate-to-vigorous physical activity) or NR (not reported).

**Notes:**

1. Give priority to whether the following quantitative indicators are reported:

| Indicator | Extraction Recommendation | Classification Reference Value |
| --- | --- | --- |
| METs | Use the value explicitly reported in the study, if available. | 1.5–3.9 = LMPA; 4.0–5.9 = MPA; ≥6.0 = MVPA |
| %HRmax or HRR (heart rate reserve) | Use values clearly reported or indirectly inferred from the text or tables. | <55% = LMPA; 55–70% = MPA; ≥70% = MVPA |
| RPE (Borg 6–20 scale) | Use the score if the Borg 6–20 scale is clearly indicated. | ≤11 = LMPA; 12–13 = MPA; ≥14 = MVPA |

*****Classification thresholds are based on standard PA-intensity conventions.

2. Although qualitative descriptions are not direct indicators of intensity, they may be used as supplementary evidence. For example, “stretching for 10 minutes” is typically classified as LMPA, whereas “treadmill running for 30 minutes at 75% HRmax” is clearly MVPA.

3. Pay close attention to intensity-related keywords. If authors explicitly use terms such as “light-to-moderate”, “moderate”, “vigorous” or “MVPA”, map these descriptors directly to the corresponding intensity category.

**L-O:** Intervention Dosage

**Notes:**

To calculate the total intervention duration, first estimate the number of hours per week and then multiply by the estimated number of intervention weeks. Apply the following rules:

1. **Record only the dose directly received by the child.** For example, if the intervention includes parent training and parents receive 1 hour of training per week while being encouraged to provide 30 minutes of intervention to the child each day, record only the 30 minutes per day directed towards the child. If the intervention is parent-mediated and involves direct parent–child interaction, include the full duration of this parent–child contact in the total intervention dose.
2. **Prioritise actual over planned duration.** If both the actual and planned intervention durations are reported, use the actual duration (or the median, if this is reported). If duration information appears in the Discussion section, ensure that this section is checked for relevant details.
3. **Duration reported in months.** If the duration is reported in months—even for 12 months—treat each month as 4.3 weeks when converting to weeks.
4. **Duration reported in years.** If the intervention is delivered weekly over multiple years, calculate duration by counting each year as 52 weeks.
5. **Missing duration.** If the intervention duration is not reported, enter “NR” (not available).

**L:** Frequency of intervention sessions per week

**M:** Duration per intervention session (minutes)
**N:** Total duration of intervention (minutes)
**O:** Total number of intervention sessions

**P:** Control group involvement in exercise intervention (i.e., non-exercising control): This variable captured whether, in a randomized controlled trial, the control group received no structured exercise/physical activity intervention (i.e., qualified as a non-exercising control).

**Yes = non-exercising control**: The control group received no additionally scheduled, structured exercise/physical activity intervention. Acceptable comparator conditions included usual care/standard rehabilitation or educational services, wait-list control, attention control, sham/placebo conditions, nutraceuticals (e.g., dietary supplements), and education-only counseling or “back school”–style health education programs; however, the control group must not have received any additional prescribed exercise or physical activity plan.

**No = exercise-involved control**: The control group was assigned any structured exercise or physical activity intervention, regardless of intensity or frequency and regardless of whether it matched the experimental modality. Examples included aerobic, resistance, or neuromuscular training; walking, running, cycling, or ball sports; yoga, tai chi, dance, or gymnastics; and stretching or range-of-motion (ROM) exercises. Any exercise exposure in the control group (including stretching) was considered inconsistent with a non-exercising control.

**NR = insufficient information/unclear**: NR was assigned when the control condition was described too vaguely to determine whether it included a structured exercise/physical activity component, or when control exposure varied substantially across participants such that a single classification could not be made.

**Q: Total number randomized to the intervention group:** The total number of participants with NDD who were randomly assigned to the intervention (exercise/physical activity) group, based on the sample size at randomization (i.e., the intention-to-treat randomized count, not the number who completed follow-up). Preferentially record the counts reported in the CONSORT flow diagram or in the Methods section (e.g., under “allocated to intervention” or “randomized to the X group”). For cluster randomized trials, record the number of individual participants rather than the number of clusters. For crossover designs, record the number of participants initially randomized to the exercise condition.

**R: Total number randomized to the control group:** The total number of participants with NDD who were randomly assigned to the control condition (e.g., non-exercising control, usual care, or standard services), using the same principles described above.

**Part III: adverse event**

Note: Search each study PDF using terms like "adverse event," "adverse effect," "side effect," complications, harm*, attrition, withdr*, and dropou to locate relevant information. If these terms are not found, carefully review the "Methods" section for safety descriptions and the "Results" section figures/tables for data on adverse events or reasons for dropout.

**S: Adverse event reporting:** The key criterion is whether the study explicitly reported adverse events/adverse effects or described monitoring for adverse events. Examples include: “No intervention-related serious adverse events were reported during the 2-year study period,” “No adverse events were observed in either group,” “No adverse effect were reported,” and “One child in the parent-training group reported feeling stressed because of the intervention.”

**Yes:** The study reported adverse events and/or safety information.

**No:** The study did not report adverse events or safety information.

**T: Total number of adverse event:** Any reported side effect, illness, injury, symptom worsening, or other specific adverse outcome should be recorded, provided that it is explicitly stated in the study, regardless of whether it is associated with the intervention.

**U: Total number of adverse effects**: This item captured the total number of adverse effects, defined as adverse events for which there were reasonable grounds to suspect a possible causal relationship with the physical activity intervention. Coding was based on a prespecified operational attribution rule rather than on general reviewer impression. An event was coded as an adverse effect only if it met at least one of the attribution criteria below.

**Explicit author attribution:** The original study authors explicitly stated or clearly implied that the event was related to, caused by, triggered by, or attributable to the intervention, trial, program, or exercise exposure. Examples of such wording included “intervention-related,” “trial-related,” “exercise-related,” “caused by the intervention,” “due to increased activity,” “protocol-related,” and “reported feeling stressed because of the intervention.”

**Clearly intervention-related event type according to the prespecified rule (based on the Cochrane Handbook).** If explicit attribution was not provided, the event was coded as an adverse effect only if all of the following conditions were met:

1. **Temporal proximity:** The event occurred during exercise participation, immediately after a session, or in close temporal relation to intervention exposure.
2. **Clinical or contextual consistency:** The event type was inherently consistent with the nature of the physical activity intervention.
3. **No explicit contradiction:** The study did not state that the event was unrelated to the intervention.
4. **Sufficient minimum detail:** The report contained enough information to support attribution, at minimum an identifiable event type and a temporal or contextual link to intervention exposure.

**Examples of event types included** exercise-induced delayed-onset muscle soreness; musculoskeletal pain occurring during or shortly after training; joint pain, soft-tissue discomfort, or transient symptom exacerbation temporally associated with exercise exposure; minor strains, sprains, or overuse-related symptoms occurring during supervised or home-based intervention activities; falls, bruises, abrasions, friction injuries, skin irritation, or blisters sustained during intervention sessions or through the use of intervention-related equipment; transient dizziness, fatigue, nausea, or shortness of breath occurring during or immediately after exercise participation; pain flare-ups following increases in exercise intensity, duration, or volume; and psychological distress clearly linked to the intervention context, such as distress related to rapid task progression, discomfort associated with required equipment, anxiety or embarrassment during participation, or difficulty adapting to the physical or social intervention environment.

**V: Adverse event monitoring approach (prespecified vs. non-prespecified):** This variable assesses whether harms/adverse events in a randomized controlled trial were captured using prespecified procedures (i.e., whether the protocol or Methods section defined adverse event outcomes in advance and specified how and when they would be collected), rather than being recorded ad hoc or without explanation.

**Prespecified recording procedures:** “Prespecified” does not require a standardized scale. It also includes studies that specify, a priori, that all spontaneously reported adverse events would be recorded and/or that adverse events would be actively queried at each visit. The key criterion is that, before trial initiation, the study clearly defined which adverse events would be recorded and how and when they would be recorded.

**Non-prespecified (not prespecified/unspecified recording procedures):** Adverse events are mentioned only incidentally in the Results or Discussion, without any prior plan or methodological description of collection procedures.

**NR:** Adverse events were not reported.

**W:** Number of adverse events in the intervention group

**X:** Number of adverse events in the control group

**Y:** Verbatim text for adverse event/adverse effect reporting: Extract key verbatim text from the article that describes adverse event/adverse effect occurrence and/or monitoring procedures, for qualitative analysis and verification (e.g., statements indicating that adverse events were monitored, definitions of adverse events, and results such as “no adverse events were observed”).

**Part IV: participant withdrawal**

**Z:** Whether participant withdrawals were reported: Did the study explicitly report participant withdrawals after randomization, including the number of withdrawals and/or the reasons for withdrawal? Please carefully examine the main text, tables, or the CONSORT flow diagram.

**AA: Total number of withdrawals:** The total number of participants who withdrew or dropped out after randomization and before the primary outcome assessment.

**AB: Are reasons for withdrawal determinable (Yes/No):** Based on the withdrawal information reported in the study, can specific reasons for withdrawal be clearly identified from the text, tables, or figures.

**AC: Number of adverse events:** This item should be coded solely on the basis of the study authors’ direct reporting in the main text, tables, supplementary materials, or CONSORT flow diagram; adverse events should not be inferred merely from the occurrence of withdrawal. According to the *Cochrane Handbook for Systematic Reviews of Interventions*, this item should be counted only when the study explicitly states that withdrawal was related to an adverse event, side effect, illness, injury, symptom exacerbation, or another specific unfavorable outcome. Nonspecific reasons for withdrawal, or reasons defined solely in administrative or procedural terms (e.g., personal reasons, scheduling conflicts, withdrawal of consent, loss to follow-up, family burden, or lack of motivation to participate), should not be classified as adverse events.

**AD: Number of adverse effects:** The total number of adverse effects reported in the study, as defined or attributed by the trial authors, extracted specifically within the context of participant withdrawal or attrition when such information is reported separately. This item should capture only those withdrawal-related events for which the study authors explicitly indicated, or clearly implied, a possible causal relationship with the intervention. If adverse effects are not reported separately in the withdrawal/attrition context, this item should be coded on the basis of the study’s reported attribution within the relevant participant flow, outcome, or safety reporting sections, provided that the event is clearly linked to withdrawal. Nonspecific withdrawal counts, or withdrawals reported without sufficient information to determine that they were due to an intervention-related adverse effect, should not be included.

**AE: Number of withdrawals due to adverse events in the intervention group:** The number of participants in the intervention group who withdrew after randomization due to adverse events/adverse effects.

**AF: Number of withdrawals due to adverse events in the control group:** The number of participants in the control group who withdrew after randomization due to adverse events/adverse effects.

**AG: Verbatim text for withdrawal reporting:** Store key verbatim excerpts describing withdrawals and withdrawal reasons to facilitate verification of judgments for AA–AF and to support qualitative analysis.

**Part V: Other**

**AH: Post-intervention follow-up for adverse events:** After the intervention ended, did the study include one or more prespecified follow-up time points at which adverse events were reassessed? In particular, determine whether follow-up reporting included safety/adverse-event information and/or withdrawals due to adverse events. (Decision criteria: (1) follow-up occurred; and (2) follow-up included adverse events or withdrawals due to adverse events.)

**AI: Notes (overlap between withdrawals and adverse event reporting):** Use this field to document the relationship, overlap, or inconsistencies between participant withdrawal reporting and adverse event/adverse effect reporting (or lack thereof). Pay particular attention to whether withdrawals and adverse events are double-counted or described in overlapping ways, and whether withdrawal information and adverse-event information appear inconsistent or mismatched.

6. **Supplementary results**

**Supplementary Table 5:** The characteristics for the studies included

| **Basic Study Information** | | | **Participant Characteristics** | | | | | **Intervention** | | | | | | | | | **Adverse Event/Reaction Reporting and Monitoring** | **Whether Participant Withdrawals Are Reported** |
| --- | --- | --- | --- | --- | --- | --- | --- | --- | --- | --- | --- | --- | --- | --- | --- | --- | --- | --- |
| **ID** | **Authors**  **(Year)** | **Country** | **NDD**  **Type** | **Age**  **(Year)** | **Male%** | **Intervention group (n)** | **Control**  **group (n)** | **Type of PA** | **Intervention**  **Setting** | **Interventionist**  **Type** | **Intervention format** | **Intensity** | **Session·wk⁻¹** | **Min·session⁻¹** | **Total Minutes** | **Total Sessions** |  |  |
| 1 | Maria et al.(2001)^15^ | Portugal | IDD | 21.40 | 100.00 | 8 | 8 | Aerobic exercise | Home or Community | Clinician | Group | MVPA | 2.60 | 17.40 | 723.84 | 41.6 | NO | NO |
| 2 | Eli et al.(2002)^16^ | Israel | IDD | 63.42 | 38.46 | 16 | 10 | Aerobic exercise | Clinic | Clinician | Individual | LMPA | 3.00 | 36.00 | 2700 | 75 | NO | Yes |
| 3 | Pauline et al.(2004)^17^ | Australia | ADHD | 10.09 | 100.00 | 11 | 8 | Neuromuscular exercise | Clinic | Clinician | Group | LMPA | 0.70 | 60.00 | 834 | 13.90 | NO | Yes |
| 4 | Anita et al.(2006)^18^ | South Africa | DCD | 7.00 | 50.00 | 11 | 8 | Neuromuscular exercise | Classroom | Clinician | Group | NR | 2.00 | 45.00 | 720 | 16 | NO | NO |
| 5 | Tarik et al.(2007)^19^ | Turkey | IDD | 11.20 | 100.00 | 16 | 14 | Aerobic exercise | Classroom | Clinician | Group | MVPA | 2.94 | 60.00 | 1764 | 29.4 | NO | NO |
| 6* | Nora et al.(2008)^20^ | Australia | IDD | 26.80 | 65.00 | 9 | 11 | Resistance / Strengthening exercise | Home or Community | Educator / Coach | Group | NR | 1.86 | NR | NR | 18.56 | Yes | Yes |
| 7 | Khalili et al.(2009)^21^ | Iran | IDD | 11.72 | 56.82 | 24 | 20 | Aerobic exercise | Clinic | Educator / Coach | Unclear | MPA | 5.00 | 30.00 | 1200 | 40 | NO | Yes |
| 8 | Sami et al.(2009)^22^ | Belgium | IDD | 18.00 | NR | 15 | 15 | Combinations | Classroom | Clinician | Group | MVPA | 3.00 | 50.00 | 1500 | 30 | NO | Yes |
| 9 | MRosety et al.(2010)^23^ | Spain | IDD | 16.26 | 100.00 | 31 | 7 | Aerobic exercise | Clinic | Clinician | Individual | MVPA | 3.00 | 52.50 | 1890 | 36 | NO | NO |
| 10 | Pitetti et al.(2010)^24^ | Turkey | IDD | 14.70 | 78.00 | 25 | 25 | Combinations | Classroom | Other | Unclear | NR | 3.00 | 45.00 | 1620 | 36 | NO | NO |
| 11* | Nora et al.(2010)^25^ | Australia | IDD | 15.60 | 73.91 | 11 | 13 | Resistance / Strengthening exercise | Home or Community | Educator / Coach | Unclear | MPA | 1.80 | NR | NR | 18 | Yes | Yes |
| 12 | Ouraniao et al.(2010)^26^ | Greece | IDD | 10.00 | 45.45 | 16 | 6 | Neuromuscular exercise | Classroom | Clinician | Individual | NR | 3.00 | NR | NR | 3 | NO | NO |
| 13 | Susan et al.(2010)^27^ | Australia | DCD | 7.08 | 76.92 | 7 | 6 | Neuromuscular exercise | Clinic | Clinician | Individual | NR | 1.00 | 30.00 | 180 | 6 | NO | Yes |
| 14* | Hung et al.(2010)^28^ | China | DCD | 8.00 | 82.61 | 12 | 11 | Neuromuscular exercise | Clinic | Combination | Combination | NR | 1.00 | 45.00 | 360 | 8 | Yes | Yes |
| 15* | Alejandro et al.(2011)^29^ | Spain | IDD | 14.58 | 50.00 | 13 | 13 | Neuromuscular exercise | Clinic | Clinician | Group | NR | 2.00 | 25.00 | 1050 | 42 | Yes | Yes |
| 16* | Ulrich et al.(2011)^30^ | USA | IDD | 12.17 | 43.48 | 36 | 36 | Neuromuscular exercise | Home or Community | Clinician | Individual | NR | 5.00 | 75.00 | 375 | 5 | NO | Yes |
| 17 | Ahmed et al.(2011)^31^ | Saudi Arabia | ADHD | 13.85 | 64.29 | 42 | 42 | Aerobic exercise | Classroom | Other | Group | MPA | 3.50 | 43.71 | 1530 | 35 | NO | NO |
| 18 | Calders et al.(2011)^32^ | Belgium | IDD | 42.00 | 40.00 | 15 | 15 | Combinations | Clinic | Clinician | Unclear | MVPA | 2.00 | 70.00 | 2800 | 40 | Yes | Yes |
| 19 | Attilio et al.(2012)^33^ | Italy | IDD | 40.10 | 59.26 | 14 | 13 | Combinations | Clinic | Clinician | Group | NR | 2.00 | 60.00 | 1440 | 24 | No | No |
| 20 | Fatimah et al.(2012)^34^ | Iran | ASD | 9.13 | 86.67 | 15 | 15 | Neuromuscular exercise | Clinic | Educator / Coach | Individual | NR | 4.00 | 72.86 | 4080 | 56 | No | Yes |
| 21* | Fong et al.(2012)^35^ | China | DCD | 7.54 | 79.54 | 21 | 23 | Neuromuscular exercise | Combination | Combination | Combination | NR | 1.00 | 60.00 | 5040 | 84 | Yes | Yes |
| 22 | Javier et al.(2013)^36^ | Spain | IDD | 24.88 | 0.00 | 11 | 9 | Aerobic exercise | Clinic | Clinician | Unclear | MPA | 3.00 | 42.50 | 1275 | 30 | No | No |
| 23* | Ching et al.(2012)^37^ | China | IDD | 15.25 | 46.74 | 46 | 46 | Combinations | Clinic | Educator / Coach | Individual | MPA | 3.00 | 35.00 | 630 | 18 | Yes | Yes |
| 24 | Mokhtar et al.(2012)^38^ | Iran | IDD | 11.13 | 50.00 | 40 | 40 | Neuromuscular exercise | Classroom | Other | Group | NR | NR | 45.00 | 540 | 12 | No | No |
| 25 | Chang et al.(2012)^39^ | China | ADHD | 10.43 | 92.50 | 20 | 20 | Aerobic exercise | Clinic | Clinician | Individual | MPA | 1.00 | 30.00 | 30 | 1 | No | No |
| 26 | Rahmat et al.(2013)^40^ | Iran | IDD | 11.16 | 100.00 | 17 | 14 | Neuromuscular exercise | Home or Community | Other | Individual | NR | 3.00 | NR | NR | 24 | No | No |
| 27 | Ahmadreza et al.(2013)^41^ | Iran | ASD | 9.13 | 86.67 | 15 | 15 | Neuromuscular exercise | Clinic | Educator / Coach | Group | NR | 4.00 | 72.86 | 4080 | 56 | No | Yes |
| 28* | Manuel et al.(2013)^42^ | Spain | IDD | 23.70 | 100.00 | 24 | 16 | Resistance / Strengthening exercise | Clinic | Clinician | Group | NR | 3.00 | NR | NR | 36 | Yes | Yes |
| 29 | Nora et al.(2013)^43^ | Australia | IDD | 18.00 | 55.88 | 34 | 34 | Resistance / Strengthening exercise | Home or Community | Educator / Coach | Individual | MVPA | 2.00 | 52.50 | 1050 | 20 | Yes | Yes |
| 30 | Paraskevi et al.(2013)^44^ | Greece | IDD | 10.30 | 77.78 | 9 | 9 | Neuromuscular exercise | Classroom | Educator / Coach | Individual | NR | 7.00 | 20.00 | 1680 | 84 | No | No |
| 31 | Paraskevi et al.(2013)-2^45^ | Greece | IDD | 15.30 | 100.00 | 10 | 9 | Neuromuscular exercise | Clinic | Clinician | Individual | NR | 2.00 | 35.00 | 980 | 28 | No | No |
| 32 | Sharif et al.(2013)^46^ | Iran | IDD | 9.34 | 50.00 | 24 | 24 | Aerobic exercise | Other | Educator / Coach | Unclear | NR | 3.00 | 45.00 | 2322 | 51.6 | No | No |
| 33* | Shirley et al.(2013)^47^ | China | DCD | 7.54 | 79.55 | 21 | 23 | Neuromuscular exercise | Combination | Combination | Combination | NR | 7.00 | 60.00 | 5040 | 84 | Yes | Yes |
| 34 | ATTILIO et al.(2014)^48^ | Italy | IDD | 40.10 | 59.26 | 14 | 13 | Aerobic exercise | Clinic | Other | Group | NR | 2.00 | 60.00 | 1440 | 24 | No | No |
| 35 | Chen et al.(2014)^49^ | United States | IDD | 20.16 | 100.00 | 12 | 8 | Aerobic exercise | Clinic | Clinician | Individual | MVPA | 1.00 | 20.00 | 20 | 1 | No | No |
| 36 | Ehab et al.(2014)^50^ | Egypt | IDD | 9.36 | 54.29 | 18 | 17 | Aerobic exercise | Clinic | Clinician | Individual | MVPA | 3.00 | 25.00 | 900 | 36 | No | Yes |
| 37* | Ordonez et al.(2013)^51^ | Spain | IDD | 24.88 | 0.00 | 11 | 9 | Aerobic exercise | Clinic | Other | Individual | MPA | 3.00 | 55.00 | 1650 | 30 | Yes | Yes |
| 38 | Lee et al.(2014)^52^ | Korea | IDD | 15.67 | 52.50 | 21 | 21 | Neuromuscular exercise | Classroom | Clinician | Unclear | NR | 2.00 | 40.00 | 640 | 16 | No | Yes |
| 39 | Rosety et al.(2014)^53^ | Spain | IDD | 24.88 | 0.00 | 11 | 9 | Aerobic exercise | Other | Other | Unclear | MPA | 3.00 | 35.00 | 1050 | 30 | No | No |
| 40* | Boer et al.(2014)^54^ | Belgium | IDD | 17.70 | 64.52 | 18 | 18 | Combinations | Classroom | Clinician | Unclear | MVPA | 2.00 | 40.00 | 1200 | 30 | Yes | Yes |
| 40-2* | Boer et al.(2014)^54^ | Belgium | IDD | 17.05 | 65.52 | 18 | 18 | Aerobic exercise | Classroom | Clinician | Unclear | MVPA | 2.00 | 40.00 | 1200 | 30 | Yes | Yes |
| 41 | Zhao et al.(2024)^55^ | China | IDD | 10.57 | 100.00 | 14 | 14 | Combinations | Clinic | Educator / Coach | Group | MPA | 3.00 | 60.00 | 1548 | 36 | Yes | Yes |
| 42 | Chan et al.(2015)^56^ | China | ASD | 10.75 | 93.50 | 22 | 22 | Neuromuscular exercise | Clinic | Clinician | Group | NR | 2.00 | 60.00 | 480 | 8 | No | Yes |
| 43 | Betsy et al.(2014)^57^ | United States | ADHD | 6.83 | 54.00 | 104 | 98 | Aerobic exercise | Classroom | Clinician | Group | MVPA | 5.00 | 31.00 | 1860 | 60 | No | No |
| 44* | Craig et al.(2015)^58^ | UK | IDD | 46.22 | 55.88 | 54 | 48 | Aerobic exercise | Home or Community | Clinician | Individual | NR | 5.00 | 30.00 | 1800 | 60 | Yes | Yes |
| 45 | Bremer et al.(2014)^59^ | Canada | ASD | 4.31 | 87.50 | 5 | 4 | Neuromuscular exercise | Clinic | Clinician | Group | NR | 1.00 | 60.00 | 720 | 12 | No | Yes |
| 46 | Ezgi et al.(2015)^60^ | Turkey | IDD | 17.12 | 77.46 | 37 | 34 | Aerobic exercise | Home or Community | Educator / Coach | Group | NR | 2.00 | 120.00 | 1920 | 16 | No | No |
| 47 | Ming et al.(2015)^61^ | China | IDD | 10.74 | 55.30 | 47 | 41 | Neuromuscular exercise | Clinic | Clinician | Individual | NR | 3.00 | 60.00 | 2880 | 48 | No | Yes |
| 48* | Nora et al.(2015)^62^ | Australia | IDD | 21.40 | 50.00 | 8 | 8 | Aerobic exercise | Home or Community | Educator / Coach | Individual | MPA | 2.00 | 45.00 | 1200 | 27 | Yes | NO |
| 49 | Lee et al.(2015)^63^ | Korea | ADHD | 8.83 | 100.00 | 9 | 9 | Combinations | Other | Other | Group | MVPA | 3.00 | 60.00 | 2160 | 36 | No | Yes |
| 50 | Yazd et al.(2015)^64^ | Iran | ADHD | 8.10 | 83.33 | 12 | 12 | Neuromuscular exercise | Clinic | Clinician | Unclear | NR | 3.00 | NR | NR | 18 | No | No |
| 51 | Ziereis et al.(2015)^65^ | Germany | ADHD | 9.45 | 74.42 | 13 | 16 | Neuromuscular exercise | Clinic | Educator / Coach | Group | NR | 1.00 | 60.00 | 720 | 12 | No | Yes |
| 51-2* | Ziereis et al.(2015)^65^ | Germany | ADHD | 9.45 | 74.42 | 14 | 16 | Neuromuscular exercise | Clinic | Educator / Coach | Group | NR | 1.00 | 60.00 | 720 | 12 | No | Yes |
| 52 | Kim et al.(2015)^66^ | Korea | IDD | 14.92 | 100.00 | 6 | 6 | Combinations | Other | Clinician | Unclear | MVPA | 4.00 | 40.00 | 1920 | 48 | No | No |
| 53 | Chien et al.(2016)^67^ | China | ADHD | 8.90 | 100.00 | 16 | 16 | Neuromuscular exercise | Clinic | Clinician | Individual | NR | 2.00 | 70.00 | 1680 | 24 | No | Yes |
| 54 | Fatimah et al.(2016)^68^ | Iran | ASD | 9.13 | 86.67 | 15 | 15 | Neuromuscular exercise | Clinic | Clinician | Combination | NR | 4.00 | 60.00 | 3360 | 56 | No | Yes |
| 55 | Hasan et al.(2016)^69^ | Iran | DCD | 7.86 | 73.33 | 15 | 15 | Resistance / Strengthening exercise | Classroom | Educator / Coach | Group | MPA | 2.00 | 60.00 | 1440 | 24 | No | Yes |
| 56 | Amini et al.(2016)^70^ | Iran | IDD | 9.00 | 100.00 | 8 | 8 | Neuromuscular exercise | Classroom | Other | Unclear | NR | 2.00 | 25.00 | 400 | 16 | No | No |
| 57 | Geladé et al.(2016)^71^ | Netherlands | ADHD | 9.63 | 75.89 | 37 | 39 | Aerobic exercise | Clinic | Other | Individual | MVPA | 3.00 | 45.00 | 1248.3 | 27.74 | No | Yes |
| 57-2 | Geladé et al.(2016)^71^ | Netherlands | ADHD | 9.63 | 75.89 | 37 | 36 | Aerobic exercise | Clinic | Other | Individual | MVPA | 3.00 | 45.00 | 1248.3 | 27.74 | NO | Yes |
| 58 | Lee et al.(2016)^72^ | Korea | IDD | 16.88 | 54.84 | 16 | 16 | Neuromuscular exercise | Classroom | Educator / Coach | Unclear | NR | 2.00 | 40.00 | 640 | 16 | No | Yes |
| 59 | Marta et al.(2016)^73^ | Italy | ASD | 8.60 | 100.00 | 15 | 13 | Neuromuscular exercise | Clinic | Educator / Coach | Group | NR | 1.00 | 65.00 | 1625 | 25 | No | Yes |
| 60 | Mema et al.(2016)^74^ | Iran | ADHD | 8.30 | 100.00 | 20 | 20 | Combinations | Clinic | Educator / Coach | Group | MVPA | 3.00 | 90.00 | 2160 | 24 | No | Yes |
| 61* | Boer et al.(2016)^75^ | South Africa | IDD | 33.30 | 62.10 | 15 | 16 | Aerobic exercise | Clinic | Clinician | Group | MVPA | 3.00 | 32.50 | 1170 | 36 | Yes | Yes |
| 61-2* | Boer et al.(2016)^75^ | South Africa | IDD | 35.40 | 58.62 | 15 | 16 | Aerobic exercise | Clinic | Clinician | Group | MVPA | 3.00 | 32.50 | 1170 | 36 | Yes | Yes |
| 62 | Sayed et al.(2016)^76^ | United States | ASD | 5.34 | 82.14 | 14 | 14 | Neuromuscular exercise | Clinic | Clinician | Individual | NR | 3.00 | 40.00 | 1200 | 30 | No | Yes |
| 63* | Sonill et al.(2016)^77^ | South Africa | DCD | 10.02 | 71.67 | 32 | 32 | Combinations | Classroom | Clinician | Group | NR | 1.00 | 30.00 | 240 | 8 | Yes | Yes |
| 64 | Sima et al.(2026)^78^ | China | ADHD | 14.38 | 96.90 | 46 | 46 | Combinations | Clinic | Clinician | Group | MVPA | 2.00 | 90.00 | 540 | 6 | NO | Yes |
| 65 | Hsu et al.(2016)^79^ | China | IDD | 17.65 | NR | 8 | 8 | Neuromuscular exercise | Other | Other | Unclear | NR | 2.00 | 40.00 | 640 | 16 | No | No |
| 65-2 | Hsu et al.(2016)^79^ | China | IDD | 17.60 | NR | 8 | 8 | Neuromuscular exercise | Other | Other | Unclear | NR | 2.00 | 40.00 | 640 | 16 | No | No |
| 66 | Atike et al.(2017)^80^ | Turkey | LD | 10.45 | NR | 10 | 10 | Combinations | Clinic | Other | Unclear | NR | 2.00 | 45.00 | 1800 | 40 | No | No |
| 67 | Pan et al.(2016)^81^ | China | ASD | 9.08 | 100.00 | 11 | 11 | Neuromuscular exercise | Clinic | Clinician | Combination | NR | 2.00 | 70.00 | 1680 | 24 | No | No |
| 68 | Pan et al.(2014)^82^ | China | ADHD | 9.51 | 100.00 | 12 | 12 | Combinations | Clinic | Clinician | Group | MVPA | 1.00 | 90.00 | 1080 | 12 | No | No |
| 69 | Ditzad et al.(2016)^83^ | Israel | ASD | 5.29 | 78.43 | 30 | 21 | Neuromuscular exercise | Home or Community | Educator / Coach | Group | NR | 1.00 | 30.00 | 390 | 13 | No | No |
| 70 | Edyta et al.(2017)^84^ | Poland | IDD | 15.06 | NR | 17 | 17 | Neuromuscular exercise | Classroom | Clinician | Individual | NR | 3.00 | 45.00 | 1620 | 36 | No | Yes |
| 71* | Speet et al.(2016)^85^ | Netherlands | IDD | 58.05 | 45.04 | 81 | 70 | Combinations | Clinic | Clinician | Group | MPA | 3.00 | 45.00 | 4644 | 103 | Yes | Yes |
| 72 | Saber et al.(2017)^86^ | Iran | ASD | 11.22 | 73.42 | 15 | 14 | Neuromuscular exercise | Classroom | Educator / Coach | Individual | NR | 3.00 | 30.00 | 720 | 24 | No | No |
| 73 | Parisa et al.(2017)^87^ | Iran | ADHD | 9.43 | 100.00 | 17 | 15 | Neuromuscular exercise | Classroom | Other | Group | NR | NR | NR | NR | 8 | No | Yes |
| 74 | Samah et al.(2017)^88^ | Egypt | DCD | 9.17 | 53.33 | 15 | 15 | Neuromuscular exercise | Other | Clinician | Individual | NR | 3.00 | 120.00 | 4644 | 38.7 | No | NO |
| 75 | Seung et al.(2017)^89^ | Korea | IDD | 19.47 | 100.00 | 8 | 8 | Aerobic exercise | Clinic | Other | Individual | MPA | 5.00 | 30.00 | 1800 | 60 | No | No |
| 76 | Lee et al.(2017)^90^ | Korea | ADHD | 8.83 | 100.00 | 9 | 9 | Combinations | Other | Other | Group | MVPA | 3.00 | 60.00 | 2160 | 36 | No | Yes |
| 77 | Silva et al.(2017)^91^ | Portugal | IDD | 39.00 | NR | 14 | 13 | Combinations | Clinic | Clinician | Combination | MVPA | 3.00 | 60.00 | 1560 | 26 | No | Yes |
| 78 | AdaW et al.(2018)^92^ | China | DCD | 7.46 | 83.45 | 51 | 94 | Neuromuscular exercise | Combination | Educator / Coach | Unclear | NR | 7.00 | 60.00 | 5040 | 84 | Yes | Yes |
| 79 | Anna et al.(2018)^93^ | Germany | ASD | 22.50 | 77.19 | 35 | 22 | Neuromuscular exercise | Clinic | Clinician | Group | NR | 1.00 | 60.00 | 600 | 10 | No | Yes |
| 80 | Toscano et al.(2018)^94^ | Brazil | ASD | 8.40 | NR | 67 | 23 | Combinations | Clinic | Educator / Coach | Group | NR | 2.00 | 40.00 | 3840 | 96 | No | Yes |
| 81 | Dilek et al.(2018)^95^ | Turkey | IDD | 21.09 | NR | 20 | 20 | Combinations | Classroom | Combination | Unclear | NR | 3.00 | 45.00 | 3483 | 77.4 | No | Yes |
| 82 | Hadi et al.(2018)^96^ | Iran | ASD | 7.82 | 100.00 | 12 | 12 | Neuromuscular exercise | Clinic | Clinician | Unclear | NR | 3.00 | 60.00 | 1440 | 24 | No | No |
| 83 | Hadi et al.(2018)-2^97^ | Iran | ASD | 7.62 | 100.00 | 25 | 25 | Neuromuscular exercise | Clinic | Clinician | Unclear | NR | 3.00 | 60.00 | 2160 | 36 | No | No |
| 84 | Meltem et al.(2018)^98^ | Turkey | IDD | 13.85 | NR | 25 | 25 | Neuromuscular exercise | Classroom | Educator / Coach | Group | NR | 3.00 | 60.00 | 2160 | 36 | No | No |
| 85 | Rezaei et al.(2018)^99^ | Iran | ADHD | 9.00 | NR | 7 | 7 | Neuromuscular exercise | Other | Other | Unclear | NR | 3.00 | 45.00 | 1080 | 24 | No | No |
| 86 | Samantha et al.(2018)^100^ | United States | ASD | 4.09 | 65.22 | 12 | 11 | Neuromuscular exercise | Combination | Educator / Coach | Combination | NR | 3.50 | 30.00 | 750 | 25 | NO | Yes |
| 87 | Valentin et al.(2018)^101^ | Switzerland | ADHD | 10.48 | 82.61 | 24 | 22 | Combinations | Home or Community | Other | Individual | MVPA | 1.00 | 15.00 | 15 | 1 | No | Yes |
| 88 | Pise et al.(2018)^102^ | India | IDD | 12.50 | 64.29 | 35 | 35 | Neuromuscular exercise | Classroom | Educator / Coach | Group | NR | 6.00 | 60.00 | 4320 | 72 | No | Yes |
| 89 | Skiba et al.(2019)^103^ | Poland | IDD | 31.18 | 50.00 | 11 | 11 | Aerobic exercise | Clinic | Clinician | Unclear | NR | 3.00 | 60.00 | 1800 | 30 | No | No |
| 90 | Amir et al.(2019)^104^ | Iran | IDD | 15.00 | 0.00 | 8 | 8 | Combinations | Classroom | Educator / Coach | Unclear | NR | 3.00 | 50.00 | 1200 | 24 | No | No |
| 90-2 | Amir et al.(2019)^104^ | Iran | IDD | 15.00 | 0.00 | 8 | 8 | Combinations | Classroom | Educator / Coach | Unclear | NR | 3.00 | 50.00 | 1200 | 24 | No | No |
| 91 | Hession et al.(2019)^105^ | Ireland | DCD | 9.43 | 77.78 | 38 | 23 | Neuromuscular exercise | Clinic | Clinician | Group | NR | 1.00 | 30.00 | 240 | 8 | No | No |
| 92 | Andy et al.(2019)^106^ | China | ASD | 9.95 | 80.00 | 25 | 25 | Neuromuscular exercise | Classroom | Clinician | Group | NR | 2.00 | 45.00 | 1080 | 24 | No | Yes |
| 93 | Cindy et al.(2019)^107^ | China | DCD | 8.46 | 60.31 | 35 | 34 | Neuromuscular exercise | Classroom | Other | Unclear | NR | 1.00 | 40.00 | 320 | 8 | No | Yes |
| 94 | HM et al.(2019)^108^ | India | ASD | 11.13 | 82.14 | 25 | 25 | Neuromuscular exercise | Clinic | Clinician | Unclear | NR | 7.00 | 40.00 | 3612 | 90.3 | No | Yes |
| 95 | Phung et al.(2019)^109^ | United States | ASD | 9.34 | 82.35 | 14 | 20 | Neuromuscular exercise | Home or Community | Educator / Coach | Group | LMPA | 2.00 | 45.00 | 1170 | 26 | No | No |
| 96 | Altaye et al.(2019)^110^ | Ethiopia | IDD | 14.41 | NR | 18 | 18 | Aerobic exercise | Other | Educator / Coach | Unclear | MPA | 3.00 | 37.50 | 1800 | 48 | Yes | NO |
| 97 | Nissim et al.(2019)^111^ | Israel | IDD | 58.60 | 43.90 | 32 | 32 | Neuromuscular exercise | Clinic | Clinician | Group | LMPA | 2.00 | 20.00 | 560 | 28 | NO | Yes |
| 98 | Arabi et al.(2019)^112^ | Iran | ASD | 8.51 | 75.00 | 15 | 15 | Neuromuscular exercise | Clinic | Clinician | Group | NR | 3.00 | 50.00 | 1500 | 30 | No | No |
| 98-2 | Arabi et al.(2019)^112^ | Iran | ASD | 8.51 | 75.00 | 15 | 15 | Neuromuscular exercise | Clinic | Clinician | Group | NR | 3.00 | 50.00 | 1500 | 30 | No | No |
| 99 | Melinna et al.(2019)^113^ | Mexico | IDD | 12 | 54.55 | 13 | 9 | Resistance / Strengthening exercise | Classroom | Other | Unclear | NR | 5.00 | 55.00 | 4400 | 80 | No | No |
| 100 | Mostafa et al.(2019)^114^ | Iran | ASD | 8.55 | 77.88 | 9 | 9 | Neuromuscular exercise | Clinic | Educator / Coach | Unclear | NR | 3.00 | 60.00 | 1080 | 18 | No | Yes |
| 101 | Joshi et al.(2019)^115^ | India | ASD | 7.00 | NR | 15 | 15 | Neuromuscular exercise | Classroom | Clinician | Unclear | NR | 5.00 | 60.00 | 3600 | 60 | No | No |
| 102 | Sanja et al.(2019)^116^ | Serbia | ASD | 10.3 | 100.00 | 5 | 5 | Neuromuscular exercise | Classroom | Educator / Coach | Individual | NR | 1.00 | 15.00 | 66 | 4.4 | No | No |
| 103 | Sara et al.(2018)^117^ | Spain | IDD | 50.19 | 51.06 | 24 | 24 | Neuromuscular exercise | Clinic | Clinician | Group | LMPA | 3.00 | 45.00 | 1620 | 36 | NO | Yes |
| 104 | Hattabi et al.(2019)^118^ | Tunisia | ADHD | 9.85 | 87.50 | 20 | 20 | Aerobic exercise | Clinic | Educator / Coach | Unclear | MPA | 3.00 | 90.00 | 3240 | 36 | No | No |
| 105 | Toktam et al.(2019)^119^ | Iran | LD | 8.6 | 100.00 | 15 | 15 | Neuromuscular exercise | Clinic | Educator / Coach | Group | NR | 3.00 | 55.00 | 1320 | 24 | No | No |
| 106 | Valentin et al.(2019)^120^ | Switzerland | ADHD | 10.48 | 82.61 | 28 | 23 | Aerobic exercise | Home or Community | Computer instruction | Individual | MVPA | 1.00 | 15.00 | 15 | 1 | No | Yes |
| 107* | Andy et al.(2020)^121^ | China | ASD | 9.78 | 85.19 | 15 | 15 | Aerobic exercise | Classroom | Educator / Coach | Unclear | NR | 4.00 | 30.00 | 1440 | 48 | NO | Yes |
| 108 | Borja et al.(2020)^122^ | Spain | IDD | 13.78 | 55.56 | 15 | 30 | Aerobic exercise | Clinic | Clinician | Group | MVPA | 3.00 | 50.00 | 5400 | 108 | Yes | Yes |
| 109 | Chung et al.(2020)^123^ | China | LD | 12.02 | 56.86 | 25 | 26 | Aerobic exercise | Clinic | Clinician | Individual | MPA | 1.00 | 30.00 | 20 | 1 | No | No |
| 110 | Elif et al.(2020)^124^ | Turkey | ADHD | 8.13 | 80.00 | 15 | 15 | Aerobic exercise | Other | Clinician | Unclear | MPA | 3.00 | 45.00 | 1080 | 24 | Yes | Yes |
| 111 | Regaieg et al.(2020)^125^ | Tunisia | IDD | 8.85 | 71.43 | 18 | 17 | Neuromuscular exercise | Classroom | Educator / Coach | Group | NR | 2.00 | 60.00 | 1200 | 20 | NO | Yes |
| 112 | Grecog et al.(2020)^126^ | ITALY | ASD | 9.25 | 85.71 | 14 | 14 | Neuromuscular exercise | Home or Community | Educator / Coach | Group | NR | 2.00 | 45.00 | 996.75 | 22.15 | No | Yes |
| 113 | Hamideh et al.(2020)^127^ | Iran | DCD | 8.50 | 100.00 | 13 | 13 | Neuromuscular exercise | Classroom | Educator / Coach | Unclear | NR | 3.00 | 45.00 | 1080 | 24 | No | No |
| 113-2 | Hamideh et al.(2020)^127^ | Iran | DCD | 8.60 | 100.00 | 13 | 13 | Neuromuscular exercise | Classroom | Educator / Coach | Unclear | NR | 3.00 | 45.00 | 1080 | 24 | No | No |
| 114 | Jalil et al.(2020)^128^ | Iran | ADHD | 8.10 | 100.00 | 15 | 15 | Neuromuscular exercise | Classroom | Educator / Coach | Unclear | NR | 3.00 | 60.00 | 1260 | 21 | No | Yes |
| 115 | Ramer et al.(2020)^129^ | United States | ADHD | 9.02 | 68.57 | 19 | 16 | Aerobic exercise | Classroom | Clinician | Group | MVPA | 5.00 | 60.00 | 3000 | 50 | No | No |
| 116 | Neto et al.(2020)^130^ | Brazil | DCD | 8.28 | 75.00 | 19 | 19 | Neuromuscular exercise | Clinic | Clinician | Individual | NR | 2.00 | 60.00 | 960 | 16 | No | Yes |
| 117 | Silva et al.(2020)^131^ | Brazil | ADHD | 12.20 | 70.00 | 18 | 15 | Aerobic exercise | Clinic | Clinician | Unclear | NR | 2.00 | 45.00 | 720 | 16 | No | Yes |
| 118* | Boer et al.(2020)^132^ | South Africa | IDD | 32.25 | 50.00 | 13 | 13 | Combinations | Clinic | Educator / Coach | Group | NR | 3.00 | 35.00 | 840 | 24 | Yes | No |
| 119* | Soori et al.(2020)^133^ | Iran | ADHD | 12.53 | 46.51 | 28 | 28 | Aerobic exercise | Other | Clinician | Group | MVPA | 3.00 | NR | NR | 18 | Yes | Yes |
| 120 | Soghra et al.(2019)^134^ | Iran | DCD | 8.90 | 0.00 | 20 | 20 | Neuromuscular exercise | Classroom | Clinician | Individual | NR | 2.00 | 30.00 | 480 | 16 | NO | Yes |
| 121 | Ansari et al.(2020)^135^ | Iran | ASD | 9.80 | 100.00 | 20 | 10 | Aerobic exercise | Home or Community | Educator / Coach | Group | NR | 2.00 | 60.00 | 1200 | 20 | NO | Yes |
| 122 | Kovačič et al.(2020)^136^ | Slovenia | IDD | NR | 50.00 | 100 | 50 | Combinations | Home or Community | Combination | Combination | NR | 3.00 | 60.00 | 2880 | 48 | NO | NO |
| 123 | Vincenzo et al.(2020)^137^ | Italy | IDD | 30.00 | NR | 8 | 8 | Neuromuscular exercise | Other | Other | Unclear | NR | 3.00 | NR | NR | 39 | NO | NO |
| 124* | Mills et al.(2020)^138^ | Australia | ASD | 8.72 | 75.00 | 4 | 4 | Combinations | Home or Community | Clinician | Group | NR | 1.00 | 45.00 | 180 | 4 | Yes | Yes |
| 125* | Sansi et al.(2020)^139^ | Turkey | ASD | 8.51 | 90.91 | 14 | 11 | Neuromuscular exercise | Classroom | Educator / Coach | Group | NR | 2.00 | 60.00 | 1440 | 24 | NO | Yes |
| 126 | Sabzi et al.(2021)^140^ | Iran | ADHD | 9.55 | NR | 23 | 23 | Aerobic exercise | Other | Other | Individual | MPA | 3.00 | 30.00 | 720 | 24 | NO | Yes |
| 127 | Tse et al.(2021)^141^ | China | ASD | 9.89 | 80.65 | 22 | 22 | Aerobic exercise | Classroom | Educator / Coach | Individual | MPA | 5.00 | 60.00 | 600 | 10 | NO | Yes |
| 128* | Diaz et al.(2021)^142^ | Spain | IDD | 28.10 | 100.00 | 18 | 18 | Resistance / Strengthening exercise | Other | Clinician | Group | NR | 3.00 | NR | NR | 36 | Yes | Yes |
| 129 | Ji et al.(2022)^143^ | China | ASD | 12.80 | 55.00 | 49 | 50 | Aerobic exercise | Classroom | Educator / Coach | Group | NR | 3.00 | 60.00 | 1080 | 18 | NO | Yes |
| 130 | Homa et al.(2022)^144^ | Iran | ASD | 8.18 | 95.00 | 40 | 20 | Combinations | Clinic | Clinician | Individual | LMPA | 3.00 | 35.00 | 840 | 24 | NO | NO |
| 131 | Iva et al.(2021)^145^ | USA | IDD | 24.60 | 66.67 | 12 | 12 | Resistance / Strengthening exercise | Home or Community | Educator / Coach | Individual | NR | 2.00 | NR | NR | 26 | Yes | Yes |
| 132 | Zhang et al.(2021)^146^ | China | IDD | 9.57 | 100.00 | 24 | 24 | Neuromuscular exercise | Home or Community | Educator / Coach | Group | NR | 5.00 | 60.00 | 14400 | 240 | NO | Yes |
| 133 | Marouli et al.(2021)^147^ | Greece | ASD | NR | 75.00 | 4 | 4 | Aerobic exercise | Classroom | Educator / Coach | Group | NR | 2.00 | 40.00 | 640 | 16 | NO | NO |
| 134 | Marta et al.(2021)^148^ | Poland | IDD | 20.74 | 69.12 | 37 | 36 | Neuromuscular exercise | Home or Community | Clinician | Group | NR | 2.00 | 60.00 | 1800 | 30 | NO | Yes |
| 135 | Maryam et al.(2023)^149^ | Pakistan | DCD | 8.80 | 42.31 | 13 | 13 | Neuromuscular exercise | Combination | Combination | Unclear | NR | 3.00 | 75.00 | 1800 | 24 | NO | Yes |
| 136 | Narges et al.(2022)^150^ | Iran | IDD | 13.00 | 55.56 | 15 | 12 | Neuromuscular exercise | Classroom | Educator / Coach | Group | NR | 3.00 | 45.00 | 405 | 9 | NO | NO |
| 137 | Hsu et al.(2019)^151^ | China | IDD | 16.62 | 85.19 | 27 | 27 | Combinations | Classroom | Educator / Coach | Group | MVPA | 3.00 | 90.00 | 3240 | 36 | NO | NO |
| 138 | Sakineh et al.(2021)^152^ | Iran | ADHD | 8.10 | 64.00 | 25 | 25 | Neuromuscular exercise | Clinic | Educator / Coach | Group | NR | 3.00 | 45.00 | 720 | 16 | NO | Yes |
| 139 | Yang et al.(2021)^153^ | China | ASD | 4.85 | 83.33 | 30 | 29 | Aerobic exercise | Clinic | Educator / Coach | Group | MPA | 5.00 | 40.00 | 2400 | 60 | NO | Yes |
| 140 | Ansari et al.(2021)^154^ | Iran | ASD | 11.10 | 100.00 | 20 | 20 | Aerobic exercise | Home or Community | Educator / Coach | Group | NR | 2.00 | 60.00 | 1200 | 20 | NO | Yes |
| 141 | Aithal et al.(2021)^155^ | UK | ASD | 10.65 | 80.77 | 10 | 16 | Neuromuscular exercise | Classroom | Clinician | Group | NR | 2.00 | 40.00 | 400 | 10 | NO | Yes |
| 142 | Singh et al.(2021)^156^ | India | ASD | 9.12 | NR | 53 | 26 | Neuromuscular exercise | Clinic | Educator / Coach | Unclear | NR | NR | 60.00 | 1440 | 24 | NO | Yes |
| 143 | Nejati et al.(2021)^157^ | Iran | ADHD | 9.43 | 86.67 | 15 | 15 | Neuromuscular exercise | Clinic | Clinician | Unclear | NR | 3.00 | 45.00 | 675 | 15 | NO | Yes |
| 144 | Jia et al.(2021)^158^ | China | ASD | NR | 75.00 | 12 | 12 | Neuromuscular exercise | Classroom | Educator / Coach | Group | NR | 3.00 | 60.00 | 2160 | 36 | NO | NO |
| 145 | Perrot et al.(2021)^159^ | France | IDD | 50.35 | 50.00 | 6 | 6 | Neuromuscular exercise | Clinic | Clinician | Individual | LMPA | 1.93 | 60.00 | 1390.2 | 23.17 | NO | Yes |
| 146 | Phung et al.(2021)^160^ | USA | ASD | 9.35 | 82.35 | 14 | 20 | Neuromuscular exercise | Home or Community | Educator / Coach | Group | NR | 1.27 | 60.00 | 1371.6 | 22.86 | NO | Yes |
| 147 | Ansari et al.(2020)^161^ | Iran | ASD | 10.73 | 100.00 | 10 | 10 | Neuromuscular exercise | Home or Community | Educator / Coach | Individual | NR | 2.00 | 51.00 | 1020 | 20 | NO | NO |
| 147-2 | Ansari et al.(2020)^161^ | Iran | ASD | 10.73 | 100.00 | 10 | 10 | Aerobic exercise | Home or Community | Educator / Coach | Group | NR | 2.00 | 60.00 | 1200 | 20 | NO | NO |
| 148 | Nejati et al.(2021)^162^ | Iran | ADHD | 9.48 | 55.17 | 15 | 15 | Neuromuscular exercise | Other | Clinician | Individual | NR | 3.00 | 45.00 | 607.5 | 13.5 | NO | Yes |
| 149 | Azab et al.(2022)^163^ | Saudi Arabia | IDD | 8.90 | 64.52 | 16 | 16 | Resistance / Strengthening exercise | Clinic | Clinician | Individual | NR | 2.00 | 15.00 | 360 | 24 | NO | Yes |
| 150* | Wang et al.(2022)^164^ | China | IDD | 14.17 | 73.33 | 15 | 15 | Combinations | Classroom | Educator / Coach | Group | MVPA | 2.00 | 60.00 | 1440 | 24 | Yes | Yes |
| 151 | Tse et al.(2022)^165^ | China | ASD | 8.50 | NR | 25 | 25 | Aerobic exercise | Classroom | Clinician | Unclear | MPA | 5.00 | 30.00 | 1500 | 50 | NO | NO |
| 152 | Andy et al.(2022)^166^ | China | ASD | 11.04 | 85.45 | 36 | 36 | Aerobic exercise | Classroom | Other | Unclear | MPA | 2.00 | 30.00 | 720 | 24 | NO | Yes |
| 153 | Ayoub et al.(2022)^167^ | Iran | DCD | 9.55 | 100.00 | 25 | 25 | Neuromuscular exercise | Classroom | Clinician | Group | NR | 3.00 | 30.00 | 720 | 24 | NO | Yes |
| 154 | Barkın et al.(2022)^168^ | Turkey | ADHD | 8.84 | 70.69 | 29 | 29 | Neuromuscular exercise | Clinic | Clinician | Unclear | NR | 2.00 | 45.00 | 720 | 16 | NO | Yes |
| 155 | Ji et al.(2022)^169^ | China | ASD | 12.80 | 55.00 | 49 | 50 | Aerobic exercise | Classroom | Educator / Coach | Unclear | MPA | 3.00 | 60.00 | 1080 | 18 | NO | Yes |
| 156* | Perić et al.(2021)^170^ | Serbia | IDD | 15.70 | 100.00 | 12 | 13 | Neuromuscular exercise | Home or Community | Educator / Coach | Group | NR | 2.00 | 60.00 | 1920 | 32 | Yes | Yes |
| 157* | Nekar et al.(2022)^171^ | Korea | ASD | 14.30 | 91.67 | 12 | 12 | Neuromuscular exercise | Clinic | Clinician | Unclear | NR | 2.00 | 30.00 | 240 | 8 | Yes | Yes |
| 158 | Erkan et al.(2022)^172^ | Turkey | ASD | 5.71 | 66.67 | 21 | 21 | Combinations | Home or Community | Caregiver | Individual | NR | 7.00 | 30.00 | 1260 | 42 | NO | Yes |
| 159 | Arslan et al.(2020)^173^ | Turkey | ASD | 10.07 | 100.00 | 7 | 7 | Combinations | Other | Clinician | Individual | LMPA | 3.00 | 60.00 | 2160 | 36 | NO | NO |
| 160 | Balayi et al.(2022)^174^ | Iran | comorbid | 9.63 | 100.00 | 15 | 15 | Neuromuscular exercise | Classroom | Educator / Coach | Group | NR | 3.00 | 60.00 | 1440 | 24 | NO | Yes |
| 161* | Hamza et al.(2022)^175^ | Tunisia | ASD | 6.34 | 90.91 | 20 | 8 | Aerobic exercise | Home or Community | Educator / Coach | Group | NR | 2.00 | 50.00 | 800 | 16 | Yes | Yes |
| 162 | Ren et al.(2022)^176^ | China | ASD | 6.50 | 80.00 | 15 | 15 | Neuromuscular exercise | Home or Community | Caregiver | Individual | NR | 1.00 | 40.00 | 480 | 12 | NO | NO |
| 163 | Jean-G et al.(2022)^177^ | USA | ASD | 9.49 | 83.78 | 76 | 72 | Combinations | Clinic | Clinician | Group | MVPA | 3.00 | 46.00 | 1104 | 24 | NO | Yes |
| 164* | Downs et al.(2023)^178^ | Australia | ASD | 20.00 | 2.63 | 19 | 19 | Aerobic exercise | Combination | Caregiver | Individual | LMPA | NR | NR | NR | NR | Yes | Yes |
| 165* | Kathryn et al.(2022)^179^ | USA | ADHD | 20.16 | 0.00 | 16 | 16 | Neuromuscular exercise | Home or Community | Educator / Coach | Unclear | MVPA | 1.50 | 90.00 | 810 | 9 | Yes | Yes |
| 166 | Lavinia et al.(2022)^180^ | Brazil | ASD | 10.19 | 80.56 | 17 | 19 | Neuromuscular exercise | Home or Community | Educator / Coach | Group | NR | 1.00 | 40.00 | 960 | 24 | NO | Yes |
| 167 | Bibro et al.(2023)^181^ | Poland | IDD | 20.86 | 69.12 | 37 | 36 | Neuromuscular exercise | Home or Community | Educator / Coach | Group | NR | 2.00 | 60.00 | 1800 | 30 | NO | Yes |
| 168 | Savage et al.(2022)^182^ | USA | Comorbid | 26.56 | 70.59 | 20 | 20 | Aerobic exercise | Home or Community | Caregiver | Individual | NR | 2.00 | 30.00 | 720 | 24 | NO | Yes |
| 169* | Zhao et al.(2022)^183^ | China | ASD | 5.90 | 62.26 | 34 | 34 | Neuromuscular exercise | Clinic | Clinician | Group | NR | 2.00 | 60.00 | 1440 | 24 | NO | Yes |
| 170 | Elshafey et al.(2022)^184^ | Egypt | IDD | 7.81 | NR | 20 | 20 | Combinations | Clinic | Other | Individual | MPA | 3.00 | 60.00 | 2160 | 36 | NO | NO |
| 171 | Orifjon et al.(2022)^185^ | Uzbekistan | DCD | 5.25 | 58.33 | 17 | 7 | Neuromuscular exercise | Classroom | Educator / Coach | Group | NR | 2.00 | 45.00 | 450 | 10 | NO | Yes |
| 172 | Orifjon et al.(2022)-2^186^ | Uzbekistan | DCD | 5.17 | 60.00 | 15 | 5 | Neuromuscular exercise | Classroom | Educator / Coach | Group | NR | 2.00 | 45.00 | 900 | 20 | NO | NO |
| 173 | Ruud et al.(2022)^187^ | Germany | IDD | 33.00 | 71.35 | 69 | 84 | Neuromuscular exercise | Home or Community | Other | Unclear | NR | 2.00 | 150.00 | 12900 | 86 | NO | Yes |
| 174 | Bahiraei et al.(2022)^188^ | Iran | IDD | 19.84 | 100.00 | 24 | 12 | Neuromuscular exercise | Classroom | Educator / Coach | Unclear | NR | 3.00 | 60.00 | 1440 | 24 | NO | Yes |
| 175 | Sakineh et al.(2022)^189^ | Iran | ADHD | 35.32 | 0.00 | 30 | 30 | Neuromuscular exercise | Clinic | Educator / Coach | Unclear | NR | 3.00 | 45.00 | 3142.35 | 69.83 | NO | Yes |
| 176 | Ludyga et al.(2022)^190^ | Germany | ADHD | 10.39 | 71.93 | 31 | 32 | Neuromuscular exercise | Other | Educator / Coach | Group | MPA | 2.00 | 60.00 | 1440 | 24 | NO | Yes |
| 177 | Seung et al.(2022)^191^ | Korea | IDD | 10.70 | 78.79 | 17 | 18 | Combinations | Clinic | Clinician | Individual | MPA | 1.00 | 60.00 | 480 | 8 | NO | Yes |
| 178 | Chang et al.(2022)^192^ | China | ADHD | 8.36 | 81.25 | 32 | 16 | Neuromuscular exercise | Classroom | Educator / Coach | Unclear | NR | 3.00 | 60.00 | 2160 | 36 | NO | Yes |
| 179 | Wang et al.(2022)^193^ | China | IDD | 13.50 | 36.67 | 15 | 15 | Combinations | Classroom | Clinician | Individual | NR | 3.00 | 40.00 | 960 | 24 | Yes | Yes |
| 180 | Shanker et al.(2022)^194^ | India | ASD | 9.70 | 81.40 | 23 | 20 | Neuromuscular exercise | Classroom | Educator / Coach | Group | NR | 1.00 | 45.00 | 540 | 12 | NO | Yes |
| 181 | Siyue et al.(2022)^195^ | China | IDD | 14.98 | 73.77 | 41 | 40 | Combinations | Classroom | Clinician | Group | MPA | 2.00 | 45.00 | 2340 | 52 | NO | Yes |
| 182 | Hattabi et al.(2022)^196^ | Tunisia | ADHD | 9.85 | 87.50 | 20 | 20 | Aerobic exercise | Other | Educator / Coach | Group | MPA | 3.00 | 90.00 | 3240 | 36 | NO | NO |
| 183 | Suheyla et al.(2022)^197^ | Turkey | IDD | 11.35 | NR | 10 | 10 | Neuromuscular exercise | Other | Other | Unclear | NR | 2.00 | 60.00 | 1440 | 24 | NO | NO |
| 184* | Cai et al.(2022)^198^ | China | IDD | 24.70 | 81.82 | 11 | 11 | Combinations | Home or Community | Educator / Coach | Group | NR | 3.00 | 60.00 | 4320 | 72 | Yes | Yes |
| 185 | Liang et al.(2022)^199^ | China | ADHD | 8.33 | 77.50 | 40 | 40 | Combinations | Home or Community | Educator / Coach | Group | MVPA | 3.00 | 60.00 | 2160 | 36 | NO | Yes |
| 186 | Sun et al.(2022)^200^ | China | IDD | 15.00 | 75.40 | 41 | 40 | Combinations | Classroom | Clinician | Unclear | MPA | 2.00 | 45.00 | 3483 | 77.4 | NO | Yes |
| 187 | Ahmet et al.(2023)^201^ | Turkey | ASD | 6.80 | 50.00 | 10 | 10 | Combinations | Other | Other | Unclear | NR | 2.00 | NR | NR | 16 | NO | NO |
| 188 | Broupi et al.(2023)^202^ | Greece | ASD | 13.50 | 50.00 | 10 | 8 | Neuromuscular exercise | Home or Community | Combination | Group | NR | 3.00 | 60.00 | 2160 | 36 | NO | NO |
| 189 | Kashi et al.(2023)^203^ | Iran | IDD | 12.89 | 61.11 | 18 | 18 | Combinations | Classroom | Educator / Coach | Unclear | NR | 1.75 | 60.00 | 1260 | 21 | NO | Yes |
| 190 | Amir et al.(2022)^204^ | Iran | ASD | 8.57 | 56.25 | 8 | 8 | Combinations | Classroom | Other | Group | MVPA | 3.00 | 70.00 | 1680 | 24 | NO | Yes |
| 191* | Cruz et al.(2023)^205^ | Spain | IDD | 34.21 | 55.77 | 30 | 30 | Resistance / Strengthening exercise | Home or Community | Educator / Coach | Group | MVPA | 3.00 | 60.00 | 2520 | 42 | NO | Yes |
| 192 | Fariqf et al.(2023)^206^ | Malaysia | IDD | 22.35 | NR | 15 | 15 | Combinations | Clinic | Educator / Coach | Unclear | LMPA | 2.00 | 25.00 | 300 | 12 | NO | NO |
| 193 | Shafaei et al.(2023)^207^ | Iran | ADHD | 9.98 | 100.00 | 15 | 15 | Neuromuscular exercise | Other | Other | Unclear | NR | 2.50 | 60.00 | 1200 | 20 | NO | NO |
| 194 | Allam et al.(2023)^208^ | Saudi Arabia | IDD | 10.21 | 100.00 | 17 | 17 | Aerobic exercise | Clinic | Clinician | Individual | MPA | 3.00 | 30.50 | 1098 | 36 | NO | NO |
| 195 | Hok et al.(2023)^209^ | China | ADHD | 10.12 | 78.79 | 17 | 16 | Aerobic exercise | Classroom | Other | Group | NR | 1.00 | 45.00 | 540 | 12 | NO | NO |
| 196 | Ji et al.(2023)^210^ | Korea | ADHD | 8.92 | 86.67 | 21 | 21 | Aerobic exercise | Clinic | Other | Unclear | MVPA | 3.00 | 50.00 | 600 | 12 | NO | Yes |
| 197 | Hassen et al.(2023)^211^ | Tunisia | ASD | 8.05 | NR | 16 | 14 | Neuromuscular exercise | Clinic | Other | Unclear | NR | 2.00 | 45.00 | 810 | 18 | NO | Yes |
| 198 | Kashi et al.(2023)^212^ | Iran | IDD | 13.89 | 100.00 | 18 | 18 | Combinations | Classroom | Educator / Coach | Unclear | MPA | 3.00 | 52.50 | 1890 | 36 | NO | NO |
| 199* | Svedell et al.(2023)^213^ | Sweden | ADHD | 37.00 | 35.71 | 9 | 5 | Combinations | Combination | Clinician | Group | MVPA | 3.00 | 50.00 | 1800 | 36 | Yes | Yes |
| 200 | Dinu et al.(2023)^214^ | UK | ADHD | 26.62 | 17.07 | 40 | 42 | Combinations | Other | Computer instruction | Individual | MPA | 1.00 | 10.00 | 10 | 1 | NO | NO |
| 201 | Prieto et al.(2023)^215^ | USA | ASD | 7.50 | 67.74 | 20 | 11 | Neuromuscular exercise | Home or Community | Caregiver | Individual | NR | 7.33 | 65.45 | 5760 | 88 | NO | Yes |
| 202 | Dehghani et al.(2023)^216^ | Iran | ASD | 9.30 | 100.00 | 12 | 12 | Aerobic exercise | Home or Community | Clinician | Unclear | NR | 3.00 | 45.00 | 1080 | 24 | Yes | Yes |
| 203* | Neslinur et al.(2023)^217^ | Turkey | IDD | 11.97 | 66.67 | 13 | 14 | Neuromuscular exercise | Clinic | Clinician | Unclear | MPA | 2.00 | 30.00 | 480 | 16 | Yes | NO |
| 204 | Seyyed et al.(2023)^218^ | Iran | IDD | 10.58 | 100.00 | 17 | 17 | Combinations | Classroom | Other | Unclear | MPA | 3.00 | 70.00 | 1680 | 24 | NO | NO |
| 205 | Sindhu et al.(2023)^219^ | India | ASD | 9.61 | 81.40 | 23 | 20 | Neuromuscular exercise | Classroom | Educator / Coach | Group | NR | 1.00 | 45.00 | 540 | 12 | NO | NO |
| 206* | Lin et al.(2023)^220^ | China | IDD | 15.63 | 85.71 | 17 | 17 | Aerobic exercise | Classroom | Other | Unclear | MVPA | 3.00 | 60.00 | 1200 | 24 | Yes | Yes |
| 207 | Wang et al.(2023)^221^ | China | IDD | 35.80 | 66.67 | 15 | 15 | Combinations | Home or Community | Educator / Coach | Group | MPA | 3.00 | 60.00 | 2160 | 36 | NO | NO |
| 208 | Jin et al.(2023)^222^ | China | ASD | 4.91 | 100.00 | 7 | 6 | Neuromuscular exercise | Clinic | Clinician | Individual | NR | 1.00 | 90.00 | 720 | 8 | NO | Yes |
| 209 | Veljkovic et al.(2024)^223^ | Serbia | IDD | 17.81 | 68.75 | 16 | 16 | Neuromuscular exercise | Classroom | Clinician | Group | NR | 2.00 | 45.00 | 1080 | 24 | Yes | NO |
| 210* | Cherni et al.(2025)^224^ | Tunisia | IDD | 14.44 | 100.00 | 15 | 15 | Neuromuscular exercise | Classroom | Educator / Coach | Group | NR | 3.00 | 45.00 | 810 | 18 | NO | Yes |
| 211 | Alaa et al.(2024)^225^ | Egypt | IDD | 9.01 | 35.00 | 20 | 20 | Combinations | Clinic | Clinician | Individual | NR | 3.00 | 90.00 | 3240 | 36 | NO | NO |
| 212 | Liseth et al.(2024)^226^ | Ecuador | IDD | 12.67 | 53.33 | 15 | 15 | Neuromuscular exercise | Classroom | Educator / Coach | Group | NR | 2.00 | 60.00 | 720 | 12 | NO | Yes |
| 213 | Kilany et al.(2024)^227^ | Egypt | IDD | 8.50 | 56.00 | 50 | 50 | Combinations | Clinic | Clinician | Individual | MVPA | 3.00 | 57.00 | 2052 | 36 | NO | NO |
| 214 | Borja et al.(2024)^228^ | Spain | IDD | 18.33 | 62.00 | 25 | 25 | Aerobic exercise | Classroom | Educator / Coach | Unclear | MVPA | 3.00 | 60.00 | 2880 | 48 | Yes | Yes |
| 215 | Borja et al.(2023)^229^ | Spain | IDD | 14.19 | 61.22 | 25 | 25 | Aerobic exercise | Classroom | Educator / Coach | Individual | NR | 3.00 | 60.00 | 3600 | 60 | Yes | Yes |
| 216 | Carla et al.(2024)^230^ | Portugal | ASD | 6.92 | 80.00 | 14 | 11 | Neuromuscular exercise | Other | Other | Unclear | NR | 1.00 | 45.00 | 1440 | 32 | NO | NO |
| 217* | Choi et al.(2024)^231^ | China | ASD | 10.02 | 77.42 | 20 | 20 | Aerobic exercise | Classroom | Educator / Coach | Unclear | MPA | 5.00 | 60.00 | 600 | 10 | Yes | Yes |
| 218 | Zhou et al.(2023)^232^ | China | ASD | 6.19 | 86.21 | 32 | 26 | Neuromuscular exercise | Clinic | Educator / Coach | Group | NR | 5.00 | 40.00 | 2400 | 60 | NO | Yes |
| 219* | Boer et al.(2024)^233^ | South Africa | IDD | 44.50 | 61.90 | 22 | 23 | Combinations | Home or Community | Clinician | Group | MPA | 3.00 | 37.50 | 675 | 18 | Yes | Yes |
| 220 | Elif et al.(2024)^234^ | Turkey | ADHD | 9.89 | 94.12 | 25 | 25 | Resistance / Strengthening exercise | Other | Clinician | Unclear | NR | 2.00 | 45.00 | 720 | 16 | Yes | Yes |
| 221 | Top et al.(2024)^235^ | Turkey | IDD | 16.88 | NR | 8 | 8 | Resistance / Strengthening exercise | Classroom | Educator / Coach | Unclear | NR | 2.00 | 60.00 | 1200 | 20 | NO | NO |
| 222 | ElifPunar et al.(2024)^236^ | Turkey | LD | 10.53 | 52.63 | 20 | 20 | Neuromuscular exercise | Clinic | Clinician | Individual | NR | 2.00 | 40.00 | 640 | 16 | NO | Yes |
| 223 | Erkan et al.(2023)^237^ | Turkey | ASD | 10.00 | 68.18 | 11 | 11 | Combinations | Home or Community | Clinician | Group | NR | 4.00 | 30.00 | 1200 | 40 | NO | Yes |
| 224 | Fahimeh et al.(2024)^238^ | Iran | ASD | 10.90 | 100.00 | 10 | 10 | Aerobic exercise | Home or Community | Educator / Coach | Group | NR | 2.00 | 60.00 | 1200 | 20 | NO | Yes |
| 225 | Sun et al.(2024)^239^ | China | ADHD | 10.10 | 75.50 | 43 | 20 | Aerobic exercise | Classroom | Educator / Coach | Group | MVPA | 2.00 | 30.00 | 480 | 16 | NO | Yes |
| 226 | Jouira et al.(2024)^240^ | Tunisia | IDD | 25.10 | 100.00 | 13 | 14 | Combinations | Home or Community | Educator / Coach | Unclear | NR | 2.00 | 42.50 | 1020 | 24 | NO | NO |
| 227 | Zourmand et al.(2025)^241^ | China | ASD | 11.50 | NR | 10 | 10 | Neuromuscular exercise | Classroom | Combination | Group | NR | 2.00 | 30.00 | 240 | 8 | NO | NO |
| 228 | Walters et al.(2024)^242^ | UK | Comorbid | 9.90 | 88.24 | 19 | 15 | Aerobic exercise | Classroom | Clinician | Group | MPA | 1.00 | 30.00 | 30 | 1 | NO | Yes |
| 229 | Duan et al.(2024)^243^ | China | IDD | 7.03 | NR | 18 | 18 | Neuromuscular exercise | Classroom | Other | Group | NR | 4.00 | 50.00 | 3600 | 72 | NO | Yes |
| 230 | Gulcan et al.(2024)^244^ | Turkey | IDD | 26.00 | 0.00 | 5 | 5 | Neuromuscular exercise | Clinic | Educator / Coach | Group | MPA | 3.00 | 70.00 | 1680 | 24 | NO | NO |
| 231 | Hamid et al.(2024)^245^ | Iran | Comorbid | 11.53 | 100.00 | 15 | 15 | Neuromuscular exercise | Classroom | Educator / Coach | Group | NR | 3.00 | 45.00 | 1080 | 24 | NO | Yes |
| 232 | Chen et al.(2024)^246^ | China | ASD | 5.17 | 83.33 | 15 | 15 | Aerobic exercise | Clinic | Other | Group | MPA | 6.00 | 30.00 | 1440 | 48 | NO | Yes |
| 233 | Lee et al.(2024)^247^ | Korea | DCD | 8.59 | 63.64 | 27 | 28 | Neuromuscular exercise | Classroom | Educator / Coach | Group | NR | 3.00 | 60.00 | 2160 | 36 | NO | NO |
| 234 | Jovicevic et al.(2024)^248^ | Slovenia | ADHD | 10.00 | 100.00 | 20 | 19 | Neuromuscular exercise | Other | Clinician | Unclear | NR | 2.00 | 60.00 | 1440 | 24 | NO | NO |
| 235 | Shuoqi et al.(2023)^249^ | China | IDD | 14.43 | 54.17 | 12 | 12 | Aerobic exercise | Classroom | Clinician | Group | MPA | 3.00 | 60.00 | 2160 | 36 | NO | Yes |
| 236* | Zhao et al.(2024)^250^ | China | ADHD | 8.40 | 78.75 | 44 | 46 | Neuromuscular exercise | Clinic | Clinician | Unclear | NR | 3.00 | 30.00 | 360 | 12 | Yes | Yes |
| 237 | Ge et al.(2024)^251^ | China | ASD | 7.10 | 90.00 | 23 | 26 | Combinations | Clinic | Educator / Coach | Group | MPA | 5.00 | 40.00 | 2400 | 60 | NO | Yes |
| 238 | Aliabad et al.(2024)^252^ | Iran | ADHD | NR | 100.00 | 9 | 9 | Aerobic exercise | Classroom | Educator / Coach | Group | MPA | 1.00 | 50.00 | 600 | 12 | NO | NO |
| 239 | Maha et al.(2024)^253^ | Pakistan | IDD | 7.98 | 58.93 | 30 | 30 | Neuromuscular exercise | Clinic | Clinician | Unclear | NR | 3.00 | 37.50 | 1350 | 36 | Yes | Yes |
| 240 | Malek et al.(2024)^254^ | Tunisia | ASD | 9.85 | 80.00 | 12 | 12 | Combinations | Other | Educator / Coach | Group | NR | 3.00 | 60.00 | 1440 | 24 | NO | Yes |
| 241 | Mariano et al.(2024)^255^ | Colombia | ADHD | 10.50 | 88.89 | 22 | 22 | Combinations | Other | Educator / Coach | Unclear | MVPA | 3.00 | 45.00 | 1620 | 36 | NO | Yes |
| 242* | Marko et al.(2024)^256^ | Croatia | IDD | 43.90 | 65.56 | 45 | 45 | Combinations | Clinic | Clinician | Group | MPA | 5.00 | 48.00 | 2880 | 60 | NO | Yes |
| 243 | Hakime et al.(2024)^257^ | Iran | ADHD | 11.16 | 0.00 | 15 | 15 | Neuromuscular exercise | Other | Other | Group | NR | 3.00 | 60.00 | 1080 | 18 | NO | Yes |
| 244 | Nazia et al.(2024)^258^ | Pakistan | IDD | 10.32 | 46.81 | 24 | 24 | Resistance / Strengthening exercise | Clinic | Combination | Individual | NR | 3.00 | 40.00 | 2880 | 72 | NO | Yes |
| 245 | Beyaz et al.(2024)^259^ | Turkey | IDD | 15.55 | 50.00 | 10 | 10 | Neuromuscular exercise | Classroom | Educator / Coach | Group | NR | 2.00 | 120.00 | 2880 | 24 | NO | NO |
| 246 | Peiting et al.(2024)^260^ | China | ASD | 10.74 | 100.00 | 16 | 15 | Aerobic exercise | Clinic | Educator / Coach | Group | MPA | 4.00 | 63.00 | 3024 | 48 | Yes | Yes |
| 247 | Lerma et al.(2024)^261^ | Colombia | ASD | 5.80 | 85.00 | 10 | 10 | Neuromuscular exercise | Clinic | Clinician | Group | NR | 3.00 | 60.00 | 1440 | 24 | NO | Yes |
| 248 | Cheong et al.(2025)^262^ | China | ASD | 4.83 | 66.67 | 15 | 15 | Neuromuscular exercise | Home or Community | Caregiver | Individual | NR | 6.00 | 35.00 | 1680 | 48 | NO | NO |
| 249 | Rao et al.(2024)^263^ | India | IDD | 13.50 | 58.67 | 24 | 26 | Neuromuscular exercise | Classroom | Clinician | Individual | LMPA | 3.00 | 25.00 | 300 | 12 | Yes | Yes |
| 250 | Rana et al.(2024)^264^ | Egypt | ASD | 9.14 | 80.00 | 15 | 15 | Aerobic exercise | Other | Clinician | Group | MVPA | 3.00 | 60.00 | 2340 | 39 | NO | NO |
| 251 | Saeed et al.(2024)^265^ | Iran | ASD | 13.38 | 100.00 | 40 | 10 | Aerobic exercise | Clinic | Clinician | Group | MVPA | 2.00 | 50.00 | 600 | 12 | NO | NO |
| 252 | Saima et al.(2024)^266^ | Pakistan | IDD | 9.00 | 69.61 | 34 | 34 | Combinations | Classroom | Educator / Coach | Group | MVPA | 3.00 | 45.00 | 1620 | 36 | NO | Yes |
| 252-2 | Saima et al.(2024)^266^ | Pakistan | IDD | 9.00 | 69.61 | 34 | 34 | Combinations | Classroom | Educator / Coach | Group | MPA | 3.00 | 45.00 | 1620 | 36 | NO | Yes |
| 253 | Ludyga et al.(2024)^267^ | Switzerland | ASD | 10.65 | 96.67 | 16 | 14 | Aerobic exercise | Clinic | Clinician | Individual | MPA | 1.00 | 20.00 | 20 | 1 | NO | Yes |
| 254 | Liu et al.(2026)^268^ | China | ADHD | 14.71 | 88.89 | 36 | 36 | Aerobic exercise | Combination | Educator / Coach | Group | MVPA | 1.00 | 60.00 | 720 | 12 | NO | Yes |
| 255 | Liang et al.(2024)^269^ | China | ADHD | 8.62 | 83.33 | 15 | 15 | Aerobic exercise | Other | Clinician | Group | MVPA | 3.00 | 60.00 | 2160 | 36 | NO | Yes |
| 256 | Cui et al.(2024)^270^ | China | ASD | 8.17 | 100.00 | 12 | 12 | Neuromuscular exercise | Clinic | Clinician | Group | MVPA | 3.00 | 80.00 | 3096 | 39 | NO | NO |
| 257 | Shen et al.(2024)^271^ | China | ASD | 15.70 | 55.88 | 17 | 17 | Aerobic exercise | Home or Community | Caregiver | Individual | MVPA | 3.00 | 30.00 | 2322 | 77.4 | NO | NO |
| 258 | Shen et al.(2024)-2^272^ | China | IDD | 16.53 | 50.00 | 19 | 19 | Combinations | Classroom | Educator / Coach | Group | MVPA | 3.00 | 45.00 | 3483 | 77 | NO | Yes |
| 259 | Ju et al.(2024)^273^ | China | ASD | 11.88 | 70.59 | 9 | 8 | Neuromuscular exercise | Classroom | Clinician | Group | NR | 3.00 | 47.50 | 1140 | 24 | NO | NO |
| 260 | Zhang et al.(2023)^274^ | China | ADHD | 8.79 | 79.07 | 22 | 21 | Combinations | Clinic | Educator / Coach | Group | MVPA | 3.00 | 60.00 | 2160 | 36 | NO | Yes |
| 261 | Huang et al.(2024)^275^ | China | ADHD | 9.84 | 71.05 | 22 | 16 | Aerobic exercise | Classroom | Educator / Coach | Group | MVPA | 2.00 | 60.00 | 960 | 16 | NO | Yes |
| 262 | Zohre et al.(2024)^276^ | Iran | IDD | 9.80 | 70.00 | 10 | 10 | Neuromuscular exercise | Classroom | Clinician | Group | NR | 3.00 | 30.00 | 630 | 21 | NO | NO |
| 263 | Anju et al.(2025)^277^ | India | ASD | 9.75 | 94.74 | 19 | 18 | Aerobic exercise | Other | Clinician | Group | MVPA | 3.00 | 30.00 | 720 | 24 | Yes | Yes |
| 264* | Anna et al.(2025)^278^ | Italy | ASD | 9.95 | 85.00 | 10 | 12 | Neuromuscular exercise | Clinic | Other | Individual | NR | 2.00 | 45.00 | 450 | 10 | NO | Yes |
| 265 | Ayoob et al.(2025)^279^ | Iran | ADHD | 8.50 | 100.00 | 20 | 20 | Aerobic exercise | Other | Other | Unclear | MPA | 2.00 | 40.00 | 960 | 24 | NO | Yes |
| 265-2 | Ayoob et al.(2025)^279^ | Iran | ADHD | 8.50 | 100.00 | 20 | 20 | Aerobic exercise | Other | Other | Unclear | MVPA | 2.00 | 40.00 | 960 | 24 | NO | Yes |
| 266 | Barkın et al.(2025)^280^ | Turkey | LD | 9.64 | 53.13 | 48 | 48 | Neuromuscular exercise | Clinic | Clinician | Unclear | NR | 2.00 | 45.00 | 720 | 16 | NO | NO |
| 267 | Betul et al.(2025)^281^ | Turkey | IDD | 5.30 | 45.00 | 10 | 10 | Combinations | Other | Educator / Coach | Group | NR | 3.00 | 40.00 | 960 | 24 | NO | NO |
| 268 | Borja et al.(2025)^282^ | Spain | IDD | 17.01 | 64.86 | 21 | 16 | Aerobic exercise | Classroom | Clinician | Group | NR | 3.00 | 90.00 | 5670 | 63 | NO | NO |
| 269 | Kaplan et al.(2025)^283^ | Turkey | ADHD | 7.25 | 52.86 | 35 | 35 | Neuromuscular exercise | Home or Community | Clinician | Individual | LMPA | 3.00 | 40.00 | 960 | 24 | NO | Yes |
| 270* | Liu et al.(2025)^284^ | China | ADHD | 14.74 | 90.00 | 40 | 40 | Aerobic exercise | Combination | Educator / Coach | Group | MVPA | 1.00 | 60.00 | 720 | 12 | Yes | Yes |
| 271 | He et al.(2025)^285^ | China | ADHD | 7.30 | 83.33 | 33 | 33 | Combinations | Clinic | Educator / Coach | Group | MVPA | 6.00 | 90.00 | 1800 | 20 | NO | Yes |
| 272 | Chayma et al.(2025)^286^ | Tunisia | ASD | 8.11 | 52.38 | 12 | 13 | Combinations | Clinic | Other | Group | NR | 3.00 | 45.00 | 1080 | 24 | NO | Yes |
| 273 | Pan et al.(2025)^287^ | China | ASD | 6.13 | 100.00 | 10 | 10 | Neuromuscular exercise | Clinic | Clinician | Group | NR | 2.00 | 60.00 | 1440 | 24 | NO | Yes |
| 274 | Huang et al.(2025)^288^ | China | ADHD | 10.21 | NR | 12 | 12 | Aerobic exercise | Clinic | Other | Group | NR | 2.00 | 80.00 | 1920 | 24 | NO | Yes |
| 275* | Zhu et al.(2025)^289^ | China | ADHD | 8.53 | 87.50 | 53 | 46 | Neuromuscular exercise | Home or Community | Caregiver | Individual | MPA | 3.00 | 52.50 | 1890 | 36 | Yes | Yes |
| 275-2* | Zhu et al.(2025)^289^ | China | ADHD | 8.53 | 87.50 | 45 | 46 | Combinations | Classroom | Educator / Coach | Group | MPA | 3.00 | 52.50 | 1890 | 36 | Yes | Yes |
| 276 | Gizem et al.(2025)^290^ | Turkey | IDD | 9.19 | 57.14 | 10 | 11 | Combinations | Classroom | Clinician | Group | NR | 2.00 | 60.00 | 2880 | 48 | NO | Yes |
| 277 | Ozcan et al.(2025)^291^ | Turkey | ASD | 4.76 | 94.12 | 17 | 17 | Neuromuscular exercise | Classroom | Combination | Group | NR | 2.00 | 60.00 | 1440 | 24 | NO | Yes |
| 278* | Mao et al.(2025)^292^ | China | ASD | 7.75 | 86.21 | 19 | 21 | Aerobic exercise | Clinic | Educator / Coach | Group | MPA | 5.00 | 45.00 | 2700 | 60 | NO | Yes |
| 279 | Samakoush et al.(2025)^293^ | Iran | IDD | 41.19 | NR | 17 | 16 | Neuromuscular exercise | Clinic | Clinician | Group | NR | 3.00 | 27.50 | 660 | 24 | NO | Yes |
| 280 | Samakoush et al.(2025)-2^294^ | Iran | IDD | 41.19 | 100.00 | 17 | 16 | Neuromuscular exercise | Clinic | Clinician | Unclear | NR | 3.00 | NR | NR | 24 | NO | Yes |
| 281 | Hamid et al.(2025)^295^ | Iran | Comorbid | 11.33 | 100.00 | 15 | 15 | Neuromuscular exercise | Clinic | Clinician | Group | NR | 3.00 | 45.00 | 1080 | 24 | NO | Yes |
| 282 | Hasan et al.(2025)^296^ | Iran | ASD | 8.92 | 100.00 | 20 | 20 | Neuromuscular exercise | Clinic | Clinician | Individual | NR | 2.00 | 30.00 | 480 | 16 | NO | Yes |
| 283 | Hossein et al.(2024)^297^ | Iran | IDD | NR | 100.00 | 12 | 12 | Combinations | Classroom | Educator / Coach | Group | NR | 3.00 | 45.00 | 540 | 12 | NO | Yes |
| 284 | Juliana et al.(2025)^298^ | Brazil | ASD | 8.60 | 100.00 | 7 | 4 | Aerobic exercise | Classroom | Clinician | Group | MPA | 1.00 | 20.00 | 20 | 1 | NO | Yes |
| 285 | Rallis et al.(2025)^299^ | Greece | IDD | 36.98 | 55.56 | 16 | 16 | Neuromuscular exercise | Clinic | Other | Unclear | NR | 3.00 | 30.00 | 900 | 30 | NO | Yes |
| 286 | Wen et al.(2025)^300^ | China | ASD | 8.30 | 75.00 | 20 | 20 | Combinations | Clinic | Combination | Group | NR | 3.00 | 60.00 | 2160 | 36 | Yes | NO |
| 287 | Lin et al.(2025)^301^ | China | ADHD | 9.44 | NR | 13 | 16 | Neuromuscular exercise | Other | Other | Group | NR | 2.00 | 20.00 | 480 | 24 | NO | NO |
| 288 | Alooche et al.(2025)^302^ | Iran | ASD | 8.86 | 68.20 | 15 | 15 | Neuromuscular exercise | Clinic | Clinician | Unclear | NR | 3.00 | 45.00 | 1080 | 24 | NO | Yes |
| 289 | Ahmed et al.(2025)^303^ | Saudi Arabia | ASD | 6.23 | 70.00 | 20 | 20 | Neuromuscular exercise | Clinic | Clinician | Individual | NR | 7.00 | 60.00 | 5418 | 90 | NO | NO |
| 290 | Morteza et al.(2025)^304^ | Iran | ASD | 10.00 | 100.00 | 15 | 15 | Neuromuscular exercise | Clinic | Educator / Coach | Unclear | NR | 3.00 | 60.00 | 1440 | 24 | NO | NO |
| 291 | Fegh et al.(2025)^305^ | Iran | IDD | 10.88 | 0.00 | 13 | 13 | Neuromuscular exercise | Home or Community | Clinician | Group | NR | 3.00 | 45.00 | 1080 | 24 | NO | Yes |
| 292 | Nihat et al.(2025)^306^ | Turkey | IDD | 12.22 | 77.78 | 9 | 9 | Neuromuscular exercise | Classroom | Clinician | Group | NR | 3.00 | 40.00 | 1200 | 30 | NO | NO |
| 293 | Wang et al.(2025)^307^ | China | ASD | 7.69 | 88.00 | 13 | 12 | Neuromuscular exercise | Classroom | Educator / Coach | Group | MVPA | 4.00 | 45.00 | 3240 | 72 | Yes | Yes |
| 294 | Qi et al.(2025)^308^ | China | ASD | 9.00 | 54.17 | 12 | 12 | Neuromuscular exercise | Clinic | Educator / Coach | Group | MPA | 4.00 | 60.00 | 2880 | 48 | NO | NO |
| 295 | Zade et al.(2025)^309^ | India | DCD | 8.77 | 30.00 | 15 | 15 | Neuromuscular exercise | Clinic | Clinician | Individual | NR | 5.00 | 30.00 | 600 | 20 | NO | Yes |
| 296 | Samira et al.(2023)^310^ | Iran | ASD | 10.14 | 100.00 | 12 | 13 | Neuromuscular exercise | Classroom | Combination | Group | NR | 3.00 | 52.50 | 1260 | 24 | NO | NO |
| 297 | Leung et al.(2025)^311^ | China | IDD | 17.00 | NR | 20 | 20 | Neuromuscular exercise | Classroom | Clinician | Group | NR | 1.00 | 75.00 | 600 | 8 | NO | NO |
| 298 | Su et al.(2025)^312^ | USA | ASD | 8.60 | 85.00 | 21 | 19 | Combinations | Combination | Clinician | Individual | NR | 2.00 | 75.00 | 1200 | 16 | NO | NO |
| 299 | Su et al.(2025)^313^ | USA | ASD | 8.70 | 84.44 | 16 | 15 | Neuromuscular exercise | Home or Community | Clinician | Individual | NR | 2.00 | 73.10 | 1169.6 | 16 | NO | Yes |
| 299-2 | Su et al.(2025)^313^ | USA | ASD | 8.70 | 84.44 | 15 | 15 | Neuromuscular exercise | Home or Community | Clinician | Individual | NR | 2.00 | 78.60 | 1257.6 | 16 | NO | Yes |
| 300 | Zhang et al.(2025)^314^ | China | ASD | 4.84 | 100.00 | 30 | 27 | Combinations | Classroom | Other | Group | MPA | 5.00 | 40.00 | 2400 | 60 | NO | Yes |
| 301 | Feng et al.(2025)^315^ | China | ASD | 4.83 | 70.00 | 15 | 15 | Neuromuscular exercise | Classroom | Educator / Coach | Group | NR | 3.00 | 35.00 | 840 | 24 | NO | Yes |
| 302 | Wu et al.(2025)^316^ | China | ASD | 8.23 | 75.00 | 20 | 20 | Neuromuscular exercise | Classroom | Computer instruction | Group | NR | 3.00 | 45.00 | 1620 | 36 | Yes | Yes |
| 303 | Fu et al.(2025)^317^ | China | ASD | 6.06 | 55.36 | 28 | 28 | Neuromuscular exercise | Classroom | Educator / Coach | Group | NR | 3.00 | 60.00 | 4320 | 72 | NO | Yes |
| 304 | Li et al.(2025)^318^ | China | ADHD | 8.40 | 84.17 | 60 | 60 | Neuromuscular exercise | Home or Community | Computer instruction | Individual | MPA | 5.00 | 30.00 | 1935 | 65 | Yes | Yes |
| 305 | Zhang et al.(2025)^319^ | China | ASD | 3.19 | 75.00 | 8 | 8 | Neuromuscular exercise | Clinic | Clinician | Unclear | NR | 4.00 | 35.00 | 1120 | 32 | NO | Yes |
| 306 | Wang et al.(2025)^320^ | China | IDD | 14.50 | NR | 8 | 8 | Combinations | Classroom | Educator / Coach | Group | NR | 3.00 | 40.00 | 1440 | 36 | NO | NO |
| 307 | Deng et al.(2025)^321^ | China | ASD | 7.84 | 50.00 | 12 | 12 | Neuromuscular exercise | Classroom | Educator / Coach | Group | MPA | 3.00 | 40.00 | 1440 | 36 | Yes | Yes |
| 308 | Shen et al.(2024)^322^ | China | IDD | 16.44 | 58.33 | 18 | 18 | Aerobic exercise | Home or Community | Combination | Individual | NR | 3.00 | 30.00 | 2322 | 77.4 | NO | Yes |
| 309 | Michael et al.(2025)^323^ | Australia | IDD | 9.50 | 73.36 | 101 | 137 | Combinations | Classroom | Educator / Coach | Group | MVPA | NR | NR | NR | NR | Yes | Yes |

**Note.** Descriptive characteristics of included studies (stratified by group); * Studies included in meta-analysis; Abbreviations: “NR” represents “Not Reported” ; NDD = Neurodevelopmental Disorder; ASD = Autism Spectrum Disorder; IDD = Intellectual Developmental Disorder; ADHD = Attention-Deficit/Hyperactivity Disorder; LD = Learning Disability; DCD = Developmental Coordination Disorder; LMPA = Light-to-moderate physical activity; MPA = Moderate physical activity; MVPA = Moderate-to-vigorous physical activity.

**Supplementary Table 6:** Summary of findings using GRADE quality assessment

| Subgroup | Studies | PAi(n) | CG(n) | Quality assessment | | | | | RR (95%CI) | Evidence Quality* |
| --- | --- | --- | --- | --- | --- | --- | --- | --- | --- | --- |
|  |  |  |  | Risk of bias | Inconsistency | Indirectness | Imprecision | Other considerations |  |  |
| **NDD Type** |  |  |  |  |  |  |  |  |  |  |
| ADHD | 8 | 272 | 263 | Serious^a)^ | Not Serious | Not Serious | Serious^b)^ | None | 1.04 [0.29, 3.71] | ⨁⨁◯◯ low |
| ASD | 10 | 167 | 156 | Serious^a)^ | Not Serious | Not Serious | Serious^b)^ | None | 0.79 [0.31, 2.03] | ⨁⨁◯◯ low |
| IDD | 26 | 671 | 689 | Serious^a)^ | Not Serious | Not Serious | Serious^b)^ | None | 1.59 [0.66, 3.84] | ⨁⨁◯◯ low |
| **PAi Type** |  |  |  |  |  |  |  |  |  |  |
| Aerobic exercise | 15 | 336 | 319 | Serious^a)^ | Not Serious | Not Serious | Serious^b)^ | None | 0.73 [0.27, 1.96] | ⨁⨁◯◯ low |
| Combinations | 13 | 442 | 465 | Serious^a)^ | Not Serious | Not Serious | Serious^b)^ | None | 1.60 [0.60, 4.25] | ⨁⨁◯◯ low |
| Neuromuscular exercise | 15 | 326 | 325 | Serious^a)^ | Not Serious | Not Serious | Serious^b)^ | None | 0.80 [0.25, 2.52] | ⨁⨁◯◯ low |
| **Intervention Setting** |  |  |  |  |  |  |  |  |  |  |
| Classroom | 11 | 310 | 344 | Serious^a)^ | Not Serious | Not Serious | Serious^b)^ | None | 0.72 [0.16, 3.20] | ⨁⨁◯◯ low |
| Clinic | 17 | 444 | 430 | Serious^a)^ | Not Serious | Not Serious | Serious^b)^ | None | 0.85 [0.34, 2.12] | ⨁⨁◯◯ low |
| Home or Community | 13 | 286 | 267 | Serious^a)^ | Not Serious | Not Serious | Serious^b)^ | None | 1.93 [0.79, 4.76] | ⨁⨁◯◯ low |
| **Interventionist** |  |  |  |  |  |  |  |  |  |  |
| Clinician | 20 | 541 | 516 | Serious^a)^ | Not Serious | Not Serious | Serious^b)^ | None | 1.44 [0.66, 3.16] | ⨁⨁◯◯ low |
| Educator / Coach | 19 | 460 | 489 | Serious^a)^ | Not Serious | Not Serious | Serious^b)^ | None | 1.10 [0.46, 2.62] | ⨁⨁◯◯ low |
| **Intervention Format** |  |  |  |  |  |  |  |  |  |  |
| Group | 26 | 684 | 691 | Serious^a)^ | Not Serious | Not Serious | Serious^b)^ | None | 1.18 [0.65, 2.15] | ⨁⨁◯◯ low |
| Individual | 9 | 274 | 260 | Serious^a)^ | Not Serious | Not Serious | Serious^b)^ | None | 0.53 [0.13, 2.14] | ⨁⨁◯◯ low |
| **Intensity** |  |  |  |  |  |  |  |  |  |  |
| MPA | 12 | 374 | 361 | Serious^a)^ | Not Serious | Not Serious | Serious^b)^ | None | 1.49 [0.47, 4.74] | ⨁⨁◯◯ low |
| MVPA | 13 | 359 | 392 | Serious^a)^ | Not Serious | Not Serious | Serious^b)^ | None | 0.97 [0.29, 3.25] | ⨁⨁◯◯ low |
| **Monitoring Procedure** |  |  |  |  |  |  |  |  |  |  |
| Prespecified | 8 | 182 | 178 | Serious^a)^ | Not Serious | Not Serious | Serious^c)^ | None | 4.32 [1.36, 13.68] | ⨁⨁◯◯ low |
| Non-prespecified | 31 | 775 | 778 | Serious^a)^ | Not Serious | Not Serious | Serious^b)^ | None | 1.42 [0.50, 2.44] | ⨁⨁◯◯ low |
|  |  |  |  |  |  |  |  |  |  |  |
| **Overall** | 45 | 1196 | 1197 | Serious^a)^ | Not Serious | Not Serious | Serious^b)^ | None | 1.14 [0.67, 1.94] | ⨁⨁◯◯ low |
| *GRADE: Quality of Clinical Evidence and Recommendation Levels:  High: The research team has strong confidence in the estimated effect size;  Moderate: The research team has moderate confidence in the estimated effect size;  Low: The research team has limited confidence in the estimated effect size;  Very low: The research team has very limited confidence in the estimated effect size. | | | | | | | | | | |

Note:

a) Risk of bias was evident. Among the studies exerting the greatest influence on the pooled effect, more than one-third of risk-of-bias items were rated as high risk, primarily due to inadequate randomization procedures and substantial performance and detection bias.

b) Although the confidence intervals were narrow, they still crossed the null value.

c) Although the confidence intervals crossed the null value, they were relatively wide.

**Supplementary figure 5.** Risk of bias assessment. a; Risk of bias traffic light plot of RoB2 assessments. b;Risk of bias summary of the RoB2 assessments.

**Supplementary figure 6.** Funnel plot of included studies

**References**

1. Van De Schoot R, De Bruin J, Schram R, et al. An open source machine learning framework for efficient and transparent systematic reviews. *Nature machine intelligence* 2021;3(2):125–33.

2. Quan Y, Tytko T, Hui B. Utilizing ASReview in screening primary studies for meta-research in SLA: A step-by-step tutorial. *Research Methods in Applied Linguistics* 2024;3(1):100101.

3. Van de Schoot R, de Bruin J. Researcher-in-the-loop for systematic reviewing of text databases. *SciNLP: Natural Language Processing and Data Mining for Scientific Text* 2020

4. Yin R, Martinengo L, Smith HE, et al. The views and experiences of older adults regarding digital mental health interventions: a systematic review ofqualitative studies. *The Lancet Healthy Longevity* 2024

5. Bloodgood M, Vijay-Shanker K. A method for stopping active learning based on stabilizing predictions and the need for user-adjustable stopping. *arXiv preprint arXiv:14095165* 2014

6. Boetje J, van de Schoot R. The SAFE procedure: a practical stopping heuristic for active learning-based screening in systematic reviews and meta-analyses. *Systematic Reviews* 2024;13(1):81.

7. Bourke M, Haddara A, Loh A, et al. Adherence to the World Health Organization’s physical activity recommendation in preschool-aged children: a systematic review and meta-analysis of accelerometer studies. *International Journal of Behavioral Nutrition and Physical Activity* 2023;20(1):52.

8. de Gans CJ, Burger P, Van den Ende E, et al. Sleep assessment using EEG-based wearables–A systematic review. *Sleep Medicine Reviews* 2024;76:101951.

9. Feil K, Fritsch J, Rhodes RE. The intention-behaviour gap in physical activity: a systematic review and meta-analysis of the action control framework. *British Journal of Sports Medicine* 2023;57(19):1265–71.

10. Ferdinands G, Schram R, de Bruin J, et al. Active learning for screening prioritization in systematic reviews-A simulation study. 2020

11. König L, Zitzmann S, Fütterer T, et al. An evaluation of the performance of stopping rules in AI‐aided screening for psychological meta‐analytical research. *Research Synthesis Methods* 2024;15(6):1120–46.

12. Teijema JJ, Hofstee L, Brouwer M, et al. Active learning-based systematic reviewing using switching classification models: the case of the onset, maintenance, and relapse of depressive disorders. *Frontiers in research metrics and analytics* 2023;8:1178181.

13. Piercy KL, Troiano RP, Ballard RM, et al. The physical activity guidelines for Americans. *Jama* 2018;320(19):2020–28.

14. Riebe D, Ehrman JK, Liguori G, et al. ACSM’s guidelines for exercise testing and prescription: American College of Sports Medicine 2018.

15. Varela AM, Sardinha LB, Pitetti KH. Effects of an aerobic rowing training regimen in young adults with Down syndrome. *Am J Ment Retard* 2001;106(2):135–44. doi: 10.1352/0895-8017(2001)106<0135:Eoaart>2.0.Co;2

16. Carmeli E, Kessel S, Coleman R, et al. Effects of a treadmill walking program on muscle strength and balance in elderly people with Down syndrome. *J Gerontol A Biol Sci Med Sci* 2002;57(2):M106–10. doi: 10.1093/gerona/57.2.m106

17. Jensen PS, Kenny DT. The effects of yoga on the attention and behavior of boys with Attention-Deficit/ hyperactivity Disorder (ADHD). *J Atten Disord* 2004;7(4):205–16. doi: 10.1177/108705470400700403

18. Pienaar AE, Lennox A. DIE EFFEK VAN 'N MOTORIESE INTERVENSIEPROGRAM GEBASEER OP 'N GEÏNTEGREERDE BENADERING VIR 5- TOT 8-JARIGE PLAASWERKERKINDERS MET DCD: FLAGH-STUDIE. *South African Journal for Research in Sport, Physical Education &amp; Recreation* 2006;28(1):69–83. doi: 10.4314/sajrs.v28i1.25932

19. Ozmen T, Ryildirim NU, Yuktasir B, et al. Effects of school-based cardiovascular-fitness training in children with mental retardation. *Pediatr Exerc Sci* 2007;19(2):171–8. doi: 10.1123/pes.19.2.171

20. Shields N, Taylor NF, Dodd KJ. Effects of a community-based progressive resistance training program on muscle performance and physical function in adults with Down syndrome: a randomized controlled trial. *Arch Phys Med Rehabil* 2008;89(7):1215–20. doi: 10.1016/j.apmr.2007.11.056

21. Khalili MA, Elkins MR. Aerobic exercise improves lung function in children with intellectual disability: a randomised trial. *Aust J Physiother* 2009;55(3):171–5. doi: 10.1016/s0004-9514(09)70077-7

22. Elmahgoub SM, Lambers S, Stegen S, et al. The influence of combined exercise training on indices of obesity, physical fitness and lipid profile in overweight and obese adolescents with mental retardation. *Eur J Pediatr* 2009;168(11):1327–33. doi: 10.1007/s00431-009-0930-3 [published Online First: 20090130]

23. Rosety-Rodriguez M, Rosety I, Fornieles-Gonzalez G, et al. A 12-week aerobic training programme reduced plasmatic allantoin in adolescents with Down syndrome. *Br J Sports Med* 2010;44(9):685–7. doi: 10.1136/bjsm.2008.052530 [published Online First: 20081121]

24. Yildirim NU, Erbahçeci F, Ergun N, et al. The effect of physical fitness training on reaction time in youth with intellectual disabilities. *Percept Mot Skills* 2010;111(1):178–86. doi: 10.2466/06.10.11.13.15.25.Pms.111.4.178-186

25. Shields N, Taylor NF. A student-led progressive resistance training program increases lower limb muscle strength in adolescents with Down syndrome: a randomised controlled trial. *J Physiother* 2010;56(3):187–93. doi: 10.1016/s1836-9553(10)70024-2

26. Matsouka O, Trigonis J, Simakis S, et al. VARIABILITY OF PRACTICE AND ENHANCEMENT OF ACQUISITION, RETENTION AND TRANSFER OF LEARNING USING AN OUTDOOR THROWING MOTOR SKILL BY CHILDREN WITH INTELLECTUAL DISABILITIES. *Studies in Physical Culture &amp; Tourism* 2010;17(2):157–64.

27. Hillier S, McIntyre A, Plummer L. Aquatic physical therapy for children with developmental coordination disorder: a pilot randomized controlled trial. *Phys Occup Ther Pediatr* 2010;30(2):111–24. doi: 10.3109/01942630903543575

28. Hung WW, Pang MY. Effects of group-based versus individual-based exercise training on motor performance in children with developmental coordination disorder: a randomized controlled study. *J Rehabil Med* 2010;42(2):122–8. doi: 10.2340/16501977-0496

29. González-Agüero A, Vicente-Rodríguez G, Gómez-Cabello A, et al. A combined training intervention programme increases lean mass in youths with Down syndrome. *Res Dev Disabil* 2011;32(6):2383–8. doi: 10.1016/j.ridd.2011.07.024 [published Online First: 20110805]

30. Ulrich DA, Burghardt AR, Lloyd M, et al. Physical activity benefits of learning to ride a two-wheel bicycle for children with Down syndrome: a randomized trial. *Phys Ther* 2011;91(10):1463–77. doi: 10.2522/ptj.20110061 [published Online First: 20110818]

31. Ahmed GM, Mohamed S. Effect of Regular Aerobic Exercises on Behavioral, Cognitive and Psychological Response in Patients with Attention Deficit-Hyperactivity Disorder. *Life Science Journal-Acta Zhengzhou University Overseas Edition* 2011;8(2):366–71.

32. Calders P, Elmahgoub S, Roman de Mettelinge T, et al. Effect of combined exercise training on physical and metabolic fitness in adults with intellectual disability: a controlled trial. *Clin Rehabil* 2011;25(12):1097–108. doi: 10.1177/0269215511407221 [published Online First: 20110817]

33. Carraro A, Gobbi E. Effects of an exercise programme on anxiety in adults with intellectual disabilities. *Res Dev Disabil* 2012;33(4):1221–6. doi: 10.1016/j.ridd.2012.02.014 [published Online First: 20120322]

34. Bahrami F, Movahedi A, Marandi SM, et al. Kata techniques training consistently decreases stereotypy in children with autism spectrum disorder. *Res Dev Disabil* 2012;33(4):1183–93. doi: 10.1016/j.ridd.2012.01.018 [published Online First: 20120322]

35. Fong SS, Tsang WW, Ng GY. Taekwondo training improves sensory organization and balance control in children with developmental coordination disorder: a randomized controlled trial. *Res Dev Disabil* 2012;33(1):85–95. doi: 10.1016/j.ridd.2011.08.023 [published Online First: 20111004]

36. Ordoñez FJ, Fornieles-Gonzalez G, Rosety MA, et al. Anti-Inflammatory Effect of Exercise, Via Reduced Leptin Levels, in Obese Women with Down Syndrome. *Int J Sports Physiol Perform* 2012 [published Online First: 20121119]

37. Lin HC, Wuang YP. Strength and agility training in adolescents with Down syndrome: a randomized controlled trial. *Res Dev Disabil* 2012;33(6):2236–44. doi: 10.1016/j.ridd.2012.06.017 [published Online First: 20120721]

38. Malekpour M, Isfahani AS, Amiri S, et al. The effect of adapted play training on motor development of students with intellectual disabilities. *International Journal of Developmental Disabilities* 2012;58(2):120–27. doi: 10.1179/2047387711y.0000000002

39. Chang YK, Liu S, Yu HH, et al. Effect of acute exercise on executive function in children with attention deficit hyperactivity disorder. *Arch Clin Neuropsychol* 2012;27(2):225–37. doi: 10.1093/arclin/acr094 [published Online First: 20120203]

40. Rahmat A, Hasan D. The effect of core stabilization exercises on factors physical fitness to mental retardation. *Sports Medicine Journal / Medicina Sportiva* 2013;9(1):2058–62.

41. Movahedi A, Bahrami F, Marandi SM, et al. Improvement in social dysfunction of children with autism spectrum disorder following long term Kata techniques training. *Research in Autism Spectrum Disorders* 2013;7(9):1054–61. doi: 10.1016/j.rasd.2013.04.012

42. Rosety-Rodriguez M, Camacho A, Rosety I, et al. Resistance circuit training reduced inflammatory cytokines in a cohort of male adults with Down syndrome. *Med Sci Monit* 2013;19:949–53. doi: 10.12659/msm.889362 [published Online First: 20131107]

43. Shields N, Taylor NF, Wee E, et al. A community-based strength training programme increases muscle strength and physical activity in young people with Down syndrome: a randomised controlled trial. *Res Dev Disabil* 2013;34(12):4385–94. doi: 10.1016/j.ridd.2013.09.022 [published Online First: 20131011]

44. Giagazoglou P, Kokaridas D, Sidiropoulou M, et al. Effects of a trampoline exercise intervention on motor performance and balance ability of children with intellectual disabilities. *Res Dev Disabil* 2013;34(9):2701–7. doi: 10.1016/j.ridd.2013.05.034 [published Online First: 20130614]

45. Giagazoglou P, Arabatzi F, Kellis E, et al. Muscle reaction function of individuals with intellectual disabilities may be improved through therapeutic use of a horse. *Res Dev Disabil* 2013;34(9):2442–8. doi: 10.1016/j.ridd.2013.04.015 [published Online First: 20130607]

46. Reza SM, Rasool H, Mansour S, et al. Effects of calcium and training on the development of bone density in children with Down syndrome. *Res Dev Disabil* 2013;34(12):4304–9. doi: 10.1016/j.ridd.2013.08.037 [published Online First: 20131021]

47. Fong SS, Chung JW, Chow LP, et al. Differential effect of Taekwondo training on knee muscle strength and reactive and static balance control in children with developmental coordination disorder: a randomized controlled trial. *Res Dev Disabil* 2013;34(5):1446–55. doi: 10.1016/j.ridd.2013.01.025 [published Online First: 20130305]

48. Carraro A, Gobbi E. Exercise intervention to reduce depressive symptoms in adults with intellectual disabilities. *Percept Mot Skills* 2014;119(1):1–5. doi: 10.2466/06.15.PMS.119c17z4

49. Chen CC, Ringenbach DR, Snow M. Treadmill walking effects on grip strength in young men with Down syndrome. *Res Dev Disabil* 2014;35(2):288–93. doi: 10.1016/j.ridd.2013.10.032 [published Online First: 20131207]

50. El Kafy EM, Helal OF. Effect of rowing on pulmonary functions in children with Down syndrome. *Pediatr Phys Ther* 2014;26(4):437–45. doi: 10.1097/pep.0000000000000072

51. Ordonez FJ, Rosety MA, Camacho A, et al. Aerobic training improved low-grade inflammation in obese women with intellectual disability. *J Intellect Disabil Res* 2014;58(6):583–90. doi: 10.1111/jir.12056 [published Online First: 20130607]

52. Lee KJ, Lee MM, Shin DC, et al. The effects of a balance exercise program for enhancement of gait function on temporal and spatial gait parameters in young people with intellectual disabilities. *J Phys Ther Sci* 2014;26(4):513–6. doi: 10.1589/jpts.26.513 [published Online First: 20140423]

53. Rosety-Rodriguez M, Diaz AJ, Rosety I, et al. Exercise reduced inflammation: but for how long after training? *J Intellect Disabil Res* 2014;58(9):874–9. doi: 10.1111/jir.12096 [published Online First: 20131113]

54. Boer PH, Meeus M, Terblanche E, et al. The influence of sprint interval training on body composition, physical and metabolic fitness in adolescents and young adults with intellectual disability: a randomized controlled trial. *Clin Rehabil* 2014;28(3):221–31. doi: 10.1177/0269215513498609 [published Online First: 20130820]

55. Zhao P, Zhu G, Chen S, et al. Effects of Aquatic Exercise and Floor Curling on Balance Ability and Lower Limb Muscle Strength in Children with Intellectual Disabilities: A Pilot Study in China. *Children (Basel)* 2024;11(1) doi: 10.3390/children11010085 [published Online First: 20240111]

56. Chan AS, Han YM, Sze SL, et al. Neuroenhancement of Memory for Children with Autism by a Mind-Body Exercise. *Front Psychol* 2015;6:1893. doi: 10.3389/fpsyg.2015.01893 [published Online First: 20151211]

57. Hoza B, Smith AL, Shoulberg EK, et al. A randomized trial examining the effects of aerobic physical activity on attention-deficit/hyperactivity disorder symptoms in young children. *J Abnorm Child Psychol* 2015;43(4):655–67. doi: 10.1007/s10802-014-9929-y

58. Melville CA, Mitchell F, Stalker K, et al. Effectiveness of a walking programme to support adults with intellectual disabilities to increase physical activity: walk well cluster-randomised controlled trial. *Int J Behav Nutr Phys Act* 2015;12:125. doi: 10.1186/s12966-015-0290-5 [published Online First: 20150929]

59. Bremer E, Balogh R, Lloyd M. Effectiveness of a fundamental motor skill intervention for 4-year-old children with autism spectrum disorder: A pilot study. *Autism* 2015;19(8):980–91. doi: 10.1177/1362361314557548 [published Online First: 20141128]

60. Ertuzun E. Effects of Leisure Education Programme Including Sportive Activities on Perceived Freedom in Leisure of Adolescents with Intellectual Disabilities. *Educational Research and Reviews* 2015;10(16):2362–69.

61. Chen MD, Tsai HY, Wang CC, et al. The effectiveness of racket-sport intervention on visual perception and executive functions in children with mild intellectual disabilities and borderline intellectual functioning. *Neuropsychiatr Dis Treat* 2015;11:2287–97. doi: 10.2147/ndt.S89083 [published Online First: 20150902]

62. Shields N, Taylor NF. The feasibility of a physical activity program for young adults with Down syndrome: A phase II randomised controlled trial. *Journal of Intellectual & Developmental Disability* 2015;40(2):115–25. doi: 10.3109/13668250.2015.1014027

63. Lee SK, Lee CM, Park JH. Effects of combined exercise on physical fitness and neurotransmitters in children with ADHD: a pilot randomized controlled study. *J Phys Ther Sci* 2015;27(9):2915–9. doi: 10.1589/jpts.27.2915 [published Online First: 20150930]

64. Taft Yazd SN, Ayatizadeh F, Dehghan F, et al. Comparing the Effects of Drug Therapy, Perceptual Motor Training, and Both Combined on the Motor Skills of School-Aged Attention Deficit Hyperactivity Disorder Children. *CNS Neurol Disord Drug Targets* 2015;14(10):1283–91. doi: 10.2174/1871527315666151111123501

65. Ziereis S, Jansen P. Effects of physical activity on executive function and motor performance in children with ADHD. *Res Dev Disabil* 2015;38:181–91. doi: 10.1016/j.ridd.2014.12.005 [published Online First: 20150103]

66. Kim CG, Lee JS. Effect of startup circuit exercise on derivatives reactive oxygen metabolites, biological antioxidant potential levels and physical fitness of adolescents boys with intellectual disabilities. *J Exerc Rehabil* 2016;12(5):483–88. doi: 10.12965/jer.1632660.330 [published Online First: 20161031]

67. Pan CY, Chu CH, Tsai CL, et al. A racket-sport intervention improves behavioral and cognitive performance in children with attention-deficit/hyperactivity disorder. *Res Dev Disabil* 2016;57:1–10. doi: 10.1016/j.ridd.2016.06.009 [published Online First: 20160623]

68. Bahrami F, Movahedi A, Marandi SM, et al. The Effect of Karate Techniques Training on Communication Deficit of Children with Autism Spectrum Disorders. *J Autism Dev Disord* 2016;46(3):978–86. doi: 10.1007/s10803-015-2643-y

69. Kordi H, Sohrabi M, Saberi Kakhki A, et al. The effect of strength training based on process approach intervention on balance of children with developmental coordination disorder. *Arch Argent Pediatr* 2016;114(6):526–33. doi: 10.5546/aap.2016.eng.526

70. Amini HA, Kalkhoran JF, Salehi M, et al. Effect of Backward Walking Training on Improves Postural Stability in Children with Down syndrome. *International Journal of Pediatrics-Mashhad* 2016;4(7):2171–81.

71. Geladé K, Janssen TW, Bink M, et al. Behavioral Effects of Neurofeedback Compared to Stimulants and Physical Activity in Attention-Deficit/Hyperactivity Disorder: A Randomized Controlled Trial. *J Clin Psychiatry* 2016;77(10):e1270–e77. doi: 10.4088/JCP.15m10149

72. Lee K, Lee M, Song C. Balance training improves postural balance, gait, and functional strength in adolescents with intellectual disabilities: Single-blinded, randomized clinical trial. *Disabil Health J* 2016;9(3):416–22. doi: 10.1016/j.dhjo.2016.01.010 [published Online First: 20160216]

73. Borgi M, Loliva D, Cerino S, et al. Effectiveness of a Standardized Equine-Assisted Therapy Program for Children with Autism Spectrum Disorder. *J Autism Dev Disord* 2016;46(1):1–9. doi: 10.1007/s10803-015-2530-6

74. Memarmoghaddam M, Torbati HT, Sohrabi M, et al. Effects of a selected exercise programon executive function of children with attention deficit hyperactivity disorder. *J Med Life* 2016;9(4):373–79.

75. Boer PH, Moss SJ. Effect of continuous aerobic vs. interval training on selected anthropometrical, physiological and functional parameters of adults with Down syndrome. *J Intellect Disabil Res* 2016;60(4):322–34. doi: 10.1111/jir.12251 [published Online First: 20160125]

76. ElGarhy S, Liu T. Effects of psychomotor intervention program on students with autism spectrum disorder. *Sch Psychol Q* 2016;31(4):491–506. doi: 10.1037/spq0000164

77. Maharaj SS, Lallie R. Does a physiotherapy programme of gross motor training influence motor function and activities of daily living in children presenting with developmental coordination disorder? *S Afr J Physiother* 2016;72(1):304. doi: 10.4102/sajp.v72i1.304 [published Online First: 20160630]

78. Dastamooz S, Wong SHS, Yang Y, et al. Efficacy of a short-term physical exercise intervention on stress biomarkers and mental health in adolescents with ADHD: A randomized controlled trial. *J Affect Disord* 2026;393(Pt A):120285. doi: 10.1016/j.jad.2025.120285 [published Online First: 20250911]

79. Hsu TY. Effects of Wii Fit(®) balance game training on the balance ability of students with intellectual disabilities. *J Phys Ther Sci* 2016;28(5):1422–6. doi: 10.1589/jpts.28.1422 [published Online First: 20160531]

80. Yilmaz A, Karakas G, Kaya HB, et al. THE EFFECTS OF SPORTIVE EXERCISES ON PHYSICAL FITNESS OF CHILDREN WITH SPECIFIC LEARNING DISABILITY. *Acta Kinesiologica* 2017;11(1):88–93.

81. Pan CY, Chu CH, Tsai CL, et al. The impacts of physical activity intervention on physical and cognitive outcomes in children with autism spectrum disorder. *Autism* 2017;21(2):190–202. doi: 10.1177/1362361316633562 [published Online First: 20160709]

82. Pan CY, Chang YK, Tsai CL, et al. Effects of Physical Activity Intervention on Motor Proficiency and Physical Fitness in Children With ADHD: An Exploratory Study. *J Atten Disord* 2017;21(9):783–95. doi: 10.1177/1087054714533192 [published Online First: 20140514]

83. Zachor DA, Vardi S, Baron-Eitan S, et al. The effectiveness of an outdoor adventure programme for young children with autism spectrum disorder: a controlled study. *Dev Med Child Neurol* 2017;59(5):550–56. doi: 10.1111/dmcn.13337 [published Online First: 20161202]

84. Mikolajczyk E, Jankowicz-Szymanska A. Dual-task functional exercises as an effective way to improve dynamic balance in persons with intellectual disability - continuation of the project. *Medical Studies-Studia Medyczne* 2017;33(2):102–09. doi: 10.5114/ms.2017.68703

85. van Schijndel-Speet M, Evenhuis HM, van Wijck R, et al. A structured physical activity and fitness programme for older adults with intellectual disabilities: results of a cluster-randomised clinical trial. *J Intellect Disabil Res* 2017;61(1):16–29. doi: 10.1111/jir.12267 [published Online First: 20160413]

86. Sotoodeh MS, Arabameri E, Panahibakhsh M, et al. Effectiveness of yoga training program on the severity of autism. *Complement Ther Clin Pract* 2017;28:47–53. doi: 10.1016/j.ctcp.2017.05.001 [published Online First: 20170509]

87. Dehkordian P, Hamid N, Beshlideh K, et al. The Effectiveness of Mindful Parenting, Social Thinking and Exercise on Quality of Life in ADHD Children. *International Journal of Pediatrics-Mashhad* 2017;5(2):4295–302. doi: 10.22038/ijp.2016.7900

88. El Shemy SA, Mohamed NE. Effect of Sensory Integration on Motor Performance and Balance in Children with Developmental Coordination Disorder: A Randomized Controlled Trial. *International Journal of Therapies &amp; Rehabilitation Research* 2017;6(1):1–9. doi: 10.5455/ijtrr.000000213

89. Kim SS. Effects of endurance exercise and half-bath on body composition, cardiorespiratory system, and arterial pulse wave velocity in men with intellectual disabilities. *J Phys Ther Sci* 2017;29(7):1216–18. doi: 10.1589/jpts.29.1216 [published Online First: 20170715]

90. Lee S-K, Song J, Park J-H. Effects of combination exercises on electroencephalography and frontal lobe executive function measures in children with ADHD: A pilot study. *Biomedical Research-India* 2017;28

91. Silva V, Campos C, Sá A, et al. Wii-based exercise program to improve physical fitness, motor proficiency and functional mobility in adults with Down syndrome. *J Intellect Disabil Res* 2017;61(8):755–65. doi: 10.1111/jir.12384 [published Online First: 20170606]

92. Ma AWW, Fong SSM, Guo X, et al. Adapted Taekwondo Training for Prepubertal Children with Developmental Coordination Disorder: A Randomized, Controlled Trial. *Sci Rep* 2018;8(1):10330. doi: 10.1038/s41598-018-28738-7 [published Online First: 20180709]

93. Mastrominico A, Fuchs T, Manders E, et al. Effects of Dance Movement Therapy on Adult Patients with Autism Spectrum Disorder: A Randomized Controlled Trial. *Behav Sci (Basel)* 2018;8(7) doi: 10.3390/bs8070061 [published Online First: 20180629]

94. Toscano CVA, Carvalho HM, Ferreira JP. Exercise Effects for Children With Autism Spectrum Disorder: Metabolic Health, Autistic Traits, and Quality of Life. *Percept Mot Skills* 2018;125(1):126–46. doi: 10.1177/0031512517743823 [published Online First: 20171209]

95. Dilek G, Ozturk C, Hepguler S, et al. The Effect of Exercise on Bone Mineral Density in Patients with Down Syndrome. *Journal of Pediatric Research* 2018;5(3):118–23. doi: 10.4274/jpr.66588

96. Moradi H, Sohrabi M, Taheri H, et al. The effects of different combinations of perceptual-motor exercises, music, and vitamin D supplementation on the nerve growth factor in children with high-functioning autism. *Complement Ther Clin Pract* 2018;31:139–45. doi: 10.1016/j.ctcp.2018.02.005 [published Online First: 20180203]

97. Moradi H, Sohrabi M, Taheri H, et al. Comparison of the effects of perceptual-motor exercises, vitamin D supplementation and the combination of these interventions on decreasing stereotypical behavior in children with autism disorder. *Int J Dev Disabil* 2018;66(2):122–32. doi: 10.1080/20473869.2018.1502068 [published Online First: 20181118]

98. Işık M, Zorba E. The effects of hemsball on the motor proficiency of students with intellectual disabilities. *Int J Dev Disabil* 2018;66(2):104–12. doi: 10.1080/20473869.2018.1488534 [published Online First: 20181013]

99. Rezaei M, Kamarzard TS, Razavi MN. The Effects of Neurofeedback, Yoga Interventions on Memory and Cognitive Activity in Children with Attention Deficit/Hyperactivity Disorder: A Randomized Controlled Trial. *Annals of Applied Sport Science* 2018;6(4):17–27.

100. Cohen SCL, Harvey DJ, Shields RH, et al. Effects of Yoga on Attention, Impulsivity, and Hyperactivity in Preschool-Aged Children with Attention-Deficit Hyperactivity Disorder Symptoms. *J Dev Behav Pediatr* 2018;39(3):200–09. doi: 10.1097/dbp.0000000000000552

101. Benzing V, Chang YK, Schmidt M. Acute Physical Activity Enhances Executive Functions in Children with ADHD. *Sci Rep* 2018;8(1):12382. doi: 10.1038/s41598-018-30067-8 [published Online First: 20180817]

102. Pise V, Pradhan B, Gharote M. Effect of yoga practices on psycho-motor abilities among intellectually disabled children. *J Exerc Rehabil* 2018;14(4):581–85. doi: 10.12965/jer.1836290.145 [published Online First: 20180824]

103. Skiba A, Marchewka J, Skiba A, et al. Evaluation of the Effectiveness of Nordic Walking Training in Improving the Gait of Persons with Down Syndrome. *Biomed Res Int* 2019;2019:6353292. doi: 10.1155/2019/6353292 [published Online First: 20190415]

104. Haghighi AH, Mohammadtaghipoor F, Hamedinia M, et al. Effect of a combined exercise program (aerobic and rebound therapy) with two different ratios on some physical and motor fitness indices in intellectually disabled girls. *Baltic Journal of Health and Physical Activity* 2019;11(1):24–33. doi: 10.29359/bjhpa.11.1.03

105. Hession CE, Law Smith MJ, Watterson D, et al. The Impact of Equine Therapy and an Audio-Visual Approach Emphasizing Rhythm and Beat Perception in Children with Developmental Coordination Disorder. *J Altern Complement Med* 2019;25(5):535–41. doi: 10.1089/acm.2017.0242 [published Online First: 20190221]

106. Tse CYA, Lee HP, Chan KSK, et al. Examining the impact of physical activity on sleep quality and executive functions in children with autism spectrum disorder: A randomized controlled trial. *Autism* 2019;23(7):1699–710. doi: 10.1177/1362361318823910 [published Online First: 20190120]

107. Sit CH, Yu JJ, Wong SH, et al. A school-based physical activity intervention for children with developmental coordination disorder: A randomized controlled trial. *Res Dev Disabil* 2019;89:1–9. doi: 10.1016/j.ridd.2019.03.004 [published Online First: 20190312]

108. Vidyashree HM, Maheshkumar K, Sundareswaran L, et al. Effect of Yoga Intervention on Short-Term Heart Rate Variability in Children with Autism Spectrum Disorder. *Int J Yoga* 2019;12(1):73–77. doi: 10.4103/ijoy.IJOY_66_17

109. Phung JN, Goldberg WA. Promoting Executive Functioning in Children with Autism Spectrum Disorder Through Mixed Martial Arts Training. *J Autism Dev Disord* 2019;49(9):3669–84. doi: 10.1007/s10803-019-04072-3

110. Altaye KZ, Mondal S, Legesse K, et al. Effects of aerobic exercise on thyroid hormonal change responses among adolescents with intellectual disabilities. *BMJ Open Sport Exerc Med* 2019;5(1):e000524. doi: 10.1136/bmjsem-2019-000524 [published Online First: 20190723]

111. Nissim M, Hutzler Y, Goldstein A. A walk on water: comparing the influence of Ai Chi and Tai Chi on fall risk and verbal working memory in ageing people with intellectual disabilities - a randomised controlled trial. *J Intellect Disabil Res* 2019;63(6):603–13. doi: 10.1111/jir.12602 [published Online First: 20190218]

112. Arabi M, Saberi Kakhki A, Sohrabi M, et al. Is visuomotor training an effective intervention for children with autism spectrum disorders? *Neuropsychiatr Dis Treat* 2019;15:3089–102. doi: 10.2147/ndt.S214991 [published Online First: 20191108]

113. Ortiz-Ortiz M, Terrazas-Ordorica KI, Cano-RodrÍGuez LE, et al. Effect of an intensive physical conditioning program on body composition and isometric strength in children with Down syndrome. *Journal of Physical Education &amp; Sport* 2019;19:897–902. doi: 10.7752/jpes.2019.s3129

114. Sarabzadeh M, Azari BB, Helalizadeh M. The effect of six weeks of Tai Chi Chuan training on the motor skills of children with Autism Spectrum Disorder. *J Bodyw Mov Ther* 2019;23(2):284–90. doi: 10.1016/j.jbmt.2019.01.007 [published Online First: 20190128]

115. Joshi RR, Rathi SS. Effect of IAYT on Physical, Behavioral and Social Communicative Functions in Autism. *International Journal of Medical Research & Health Sciences* 2019;8(3):168–72.

116. Vukićević S, Đorđević M, Glumbić N, et al. A Demonstration Project for the Utility of Kinect-Based Educational Games to Benefit Motor Skills of Children with ASD. *Percept Mot Skills* 2019;126(6):1117–44. doi: 10.1177/0031512519867521 [published Online First: 20190807]

117. Cortés‐Amador S, Carrasco JJ, Sempere‐Rubio N, et al. Effects of a vestibular physiotherapy protocol on adults with intellectual disability in the prevention of falls: A multi‐centre clinical trial. *Journal of Applied Research in Intellectual Disabilities* 2019;32(2):359–67. doi: 10.1111/jar.12531

118. Hattabi S, Bouallegue M, Ben Yahya H, et al. Rehabilitation of ADHD children by sport intervention: a Tunisian experience. *Tunis Med* 2019;97(7):874–81.

119. Emami Kashfi T, Sohrabi M, Saberi Kakhki A, et al. Effects of a Motor Intervention Program on Motor Skills and Executive Functions in Children With Learning Disabilities. *Percept Mot Skills* 2019;126(3):477–98. doi: 10.1177/0031512519836811 [published Online First: 20190316]

120. Benzing V, Schmidt M. The effect of exergaming on executive functions in children with ADHD: A randomized clinical trial. *Scand J Med Sci Sports* 2019;29(8):1243–53. doi: 10.1111/sms.13446 [published Online First: 20190523]

121. Tse ACY. Brief Report: Impact of a Physical Exercise Intervention on Emotion Regulation and Behavioral Functioning in Children with Autism Spectrum Disorder. *J Autism Dev Disord* 2020;50(11):4191–98. doi: 10.1007/s10803-020-04418-2

122. Suarez-Villadat B, Luna-Oliva L, Acebes C, et al. The effect of swimming program on body composition levels in adolescents with Down syndrome. *Res Dev Disabil* 2020;102:103643. doi: 10.1016/j.ridd.2020.103643 [published Online First: 20200508]

123. Huang CJ, Tu HY, Hsueh MC, et al. Effects of Acute Aerobic Exercise on Executive Function in Children With and Without Learning Disability: A Randomized Controlled Trial. *Adapt Phys Activ Q* 2020;37(4):404–22. doi: 10.1123/apaq.2019-0108 [published Online First: 20200930]

124. Durgut E, Orengul AC, Algun ZC. Comparison of the effects of treadmill and vibration training in children with attention deficit hyperactivity disorder: A randomized controlled trial. *NeuroRehabilitation* 2020;47(2):121–31. doi: 10.3233/nre-203040

125. Regaieg G, Kermarrec G, Sahli S. Designed game situations enhance fundamental movement skills in children with Down syndrome. *J Intellect Disabil Res* 2020;64(4):271–79. doi: 10.1111/jir.12717 [published Online First: 20200128]

126. Greco G, De Ronzi R. Effect of Karate training on social, emotional, and executive functioning in children with autism spectrum disorder. *Journal of Physical Education &amp; Sport* 2020;20(4):1637–45. doi: 10.7752/jpes.2020.04223

127. Jahanbakhsh H, Sohrabi M, Kakhki AS, et al. The effect of task-specific balance training program in dual-task and singletask conditions on balance performance in children with developmental coordination disorder. *Acta Gymnica* 2020;50(1):28–37. doi: 10.5507/ag.2020.003

128. Moradi J, Jalali S, Bucci MP. Effects of Balance Training on Postural Control of Children with Attention Deficit/Hyperactivity Disorder. *Iranian Journal of Pediatrics* 2020;30(4) doi: 10.5812/ijp.95542

129. Ramer JD, Santiago-Rodríguez ME, Davis CL, et al. Exercise and Academic Performance Among Children With Attention-Deficit Hyperactivity Disorder and Disruptive Behavior Disorders: A Randomized Controlled Trial. *Pediatr Exerc Sci* 2020;32(3):140–49. doi: 10.1123/pes.2019-0224 [published Online First: 20200525]

130. Cavalcante Neto JL, Steenbergen B, Wilson P, et al. Is Wii-based motor training better than task-specific matched training for children with developmental coordination disorder? A randomized controlled trial. *Disabil Rehabil* 2020;42(18):2611–20. doi: 10.1080/09638288.2019.1572794 [published Online First: 20190222]

131. Silva LAD, Doyenart R, Henrique Salvan P, et al. Swimming training improves mental health parameters, cognition and motor coordination in children with Attention Deficit Hyperactivity Disorder. *Int J Environ Health Res* 2020;30(5):584–92. doi: 10.1080/09603123.2019.1612041 [published Online First: 20190513]

132. Boer PH. The effect of 8 weeks of freestyle swim training on the functional fitness of adults with Down syndrome. *J Intellect Disabil Res* 2020;64(10):770–81. doi: 10.1111/jir.12768 [published Online First: 20200817]

133. Soori R, Goodarzvand F, Akbarnejad A, et al. Effect of high-intensity interval training on clinical and laboratory parameters of adolescents with attention deficit hyperactivity disorder. *Science & Sports* 2020;35(4):207–15. doi: 10.1016/j.scispo.2019.08.002

134. EbrahimiSani S, Sohrabi M, Taheri H, et al. Effects of virtual reality training intervention on predictive motor control of children with DCD - A randomized controlled trial. *Res Dev Disabil* 2020;107:103768. doi: 10.1016/j.ridd.2020.103768 [published Online First: 20201108]

135. Kolachahi SA, AdibSaber F, Zidashti ZH, et al. Water-based training in combined with vitamin D supplementation improves lipid profile in children with ASD. *Research in Autism Spectrum Disorders* 2020;76 doi: 10.1016/j.rasd.2020.101603

136. Kovačič T, Kovačič M, Ovsenik R, et al. The impact of multicomponent programmes on balance and fall reduction in adults with intellectual disabilities: a randomised trial. *J Intellect Disabil Res* 2020;64(5):381–94. doi: 10.1111/jir.12727 [published Online First: 20200320]

137. Latorella V, Di Domenico F, Altavilla G. Neuromuscular adaptations to a motor skills training program for adults with intellectual disabilities. *Journal of Human Sport and Exercise* 2020;15:S676–S87. doi: 10.14198/jhse.2020.15.Proc3.20

138. Mills W, Kondakis N, Orr R, et al. Does Hydrotherapy Impact Behaviours Related to Mental Health and Well-Being for Children with Autism Spectrum Disorder? A Randomised Crossover-Controlled Pilot Trial. *Int J Environ Res Public Health* 2020;17(2) doi: 10.3390/ijerph17020558 [published Online First: 20200115]

139. Sansi A, Nalbant S, Ozer D. Effects of an Inclusive Physical Activity Program on the Motor Skills, Social Skills and Attitudes of Students with and without Autism Spectrum Disorder. *J Autism Dev Disord* 2021;51(7):2254–70. doi: 10.1007/s10803-020-04693-z

140. Sabzi AH, Dana A, Salehian MH, et al. The Effect of Water Treadmill Exercise on Children with Attention Deficit Hyperactivity Disorder. *International Journal of Pediatrics-Mashhad* 2021;9(6):13671–81. doi: 10.22038/ijp.2021.57015.4466

141. Tse ACY, Anderson DI, Liu VHL, et al. Improving Executive Function of Children with Autism Spectrum Disorder through Cycling Skill Acquisition. *Med Sci Sports Exerc* 2021;53(7):1417–24. doi: 10.1249/mss.0000000000002609

142. Diaz AJ, Rosety I, Ordonez FJ, et al. Effects of Resistance Training in Muscle Mass and Markers of Muscle Damage in Adults with Down Syndrome. *Int J Environ Res Public Health* 2021;18(17) doi: 10.3390/ijerph18178996 [published Online First: 20210826]

143. Ji C, Yang J. Effects of Physical Exercise and Virtual Training on Visual Attention Levels in Children with Autism Spectrum Disorders. *Brain Sci* 2021;12(1) doi: 10.3390/brainsci12010041 [published Online First: 20211229]

144. Rafiei Milajerdi H, Sheikh M, Najafabadi MG, et al. The Effects of Physical Activity and Exergaming on Motor Skills and Executive Functions in Children with Autism Spectrum Disorder. *Games Health J* 2021;10(1):33–42. doi: 10.1089/g4h.2019.0180 [published Online First: 20201223]

145. Obrusnikova I, Cavalier AR, Suminski RR, et al. A Resistance Training Intervention for Adults With Intellectual Disability in the Community: A Pilot Randomized Clinical Trial. *Adapt Phys Activ Q* 2021;38(4):546–68. doi: 10.1123/apaq.2020-0218 [published Online First: 20210520]

146. Zhang L, Zhu X, Haegele JA, et al. Effects of a one-year physical activity intervention on fundamental movement skills of boys with severe intellectual disabilities. *Res Dev Disabil* 2021;114:103980. doi: 10.1016/j.ridd.2021.103980 [published Online First: 20210510]

147. Marouli EA, Venetsanou F, Kambas A, et al. The effect of a Greek traditional dance programme on the social skills of children with autism spectrum disorder. *European Psychomotricity Journal* 2021;13:35–44.

148. Bibro MA, Zarow R. The Influence of Climbing Activities on Physical Fitness of People with Intellectual Disabilities. *International Journal of Disability Development and Education* 2023;70(4):530–39. doi: 10.1080/1034912x.2021.1895085

149. Sulaiman M, Baig MO, Memon SI, et al. Effects of Trampoline Exercises in Children with Developmental Coordination Disorder: A Randomized Controlled Trial. *Physikalische Medizin Rehabilitationsmedizin Kurortmedizin* 2022;32(05):279–84. doi: 10.1055/a-1755-8655

150. Noghondar NV, Sohrabi M, Taheri HR, et al. The effect of training on variability and accuracy of overhand throwing in children with Down syndrome. *Int J Dev Disabil* 2021;67(4):237–44. doi: 10.1080/20473869.2019.1574388 [published Online First: 20190726]

151. Hsu PJ, Yeh HL, Tsai CL, et al. Effects of a Floor Hockey Intervention on Motor Proficiency, Physical Fitness, and Adaptive Development in Youths with Mild Intellectual Disabilities. *Int J Environ Res Public Health* 2021;18(13) doi: 10.3390/ijerph18137059 [published Online First: 20210701]

152. Soltani Kouhbanani S, Rothenberger A. Perceptual-Motor Skills Reconstruction Program Improves Executive Functions in Children with Attention-Deficit/Hyperactivity Disorder. *Sustainability* 2021;13(11) doi: 10.3390/su13116210

153. Yang S, Liu Z, Xiong X, et al. Effects of Mini-Basketball Training Program on Social Communication Impairment and Executive Control Network in Preschool Children with Autism Spectrum Disorder. *Int J Environ Res Public Health* 2021;18(10) doi: 10.3390/ijerph18105132 [published Online First: 20210512]

154. Ansari S, AdibSaber F, Elmieh A, et al. The effect of water-based intervention on sleep habits and two sleep-related cytokines in children with autism. *Sleep Med* 2021;82:78–83. doi: 10.1016/j.sleep.2021.03.045 [published Online First: 20210420]

155. Aithal S, Karkou V, Makris S, et al. A Dance Movement Psychotherapy Intervention for the Wellbeing of Children With an Autism Spectrum Disorder: A Pilot Intervention Study. *Front Psychol* 2021;12:588418. doi: 10.3389/fpsyg.2021.588418 [published Online First: 20210719]

156. Sankhala SS, Nagendra HR, Deepeshwar S. Changes in Bioenergy Field of Children with Autism following Non-pharmacological Interventions: A Randomized Controlled Study. *International Journal of Medicine &amp; Public Health* 2021;11(1):57–62. doi: 10.5530/ijmedph.2021.1.11

157. Nejati V, Derakhshan Z. The effect of physical activity with and without cognitive demand on the improvement of executive functions and behavioral symptoms in children with ADHD. *Expert Rev Neurother* 2021;21(5):607–14. doi: 10.1080/14737175.2021.1912600 [published Online First: 20210414]

158. Jia W, Xie J. IMPROVEMENT OF THE HEALTH OF PEOPLE WITH AUTISM SPECTRUM DISORDER BY EXERCISE. *Revista Brasileira De Medicina Do Esporte* 2021;27(3):282–85. doi: 10.1590/1517-8692202127032021_0081

159. Perrot A, Maillot P, Le Foulon A, et al. Effect of Exergaming on Physical Fitness, Functional Mobility, and Cognitive Functioning in Adults With Down Syndrome. *Am J Intellect Dev Disabil* 2021;126(1):34–44. doi: 10.1352/1944-7558-126.1.34

160. Phung JN, Goldberg WA. Mixed martial arts training improves social skills and lessens problem behaviors in boys with Autism Spectrum Disorder. *Research in Autism Spectrum Disorders* 2021;83 doi: 10.1016/j.rasd.2021.101758

161. Ansari S, Hosseinkhanzadeh AA, AdibSaber F, et al. The Effects of Aquatic Versus Kata Techniques Training on Static and Dynamic Balance in Children with Autism Spectrum Disorder. *J Autism Dev Disord* 2021;51(9):3180–86. doi: 10.1007/s10803-020-04785-w [published Online First: 20201118]

162. Nejati V. Balance-based Attentive Rehabilitation of Attention Networks (BARAN) improves executive functions and ameliorates behavioral symptoms in children with ADHD. *Complement Ther Med* 2021;60:102759. doi: 10.1016/j.ctim.2021.102759 [published Online First: 20210709]

163. Azab AR, Mahmoud WS, Basha MA, et al. Distinct effects of trampoline-based stretch-shortening cycle exercises on muscle strength and postural control in children with Down syndrome: a randomized controlled study. *Eur Rev Med Pharmacol Sci* 2022;26(6):1952–62. doi: 10.26355/eurrev_202203_28343

164. Wang A, Bu D, Yu S, et al. Effects of a School-Based Physical Activity Intervention for Obesity, Health-Related Physical Fitness, and Blood Pressure in Children with Intellectual Disability: A Randomized Controlled Trial. *Int J Environ Res Public Health* 2022;19(19) doi: 10.3390/ijerph191912015 [published Online First: 20220922]

165. Tse ACY, Lee PH, Lau EYY, et al. Study protocol for a randomized controlled trial comparing the effectiveness of physical exercise and melatonin supplement on treating sleep disturbance in children with autism spectrum disorders. *PLoS One* 2022;17(7):e0270428. doi: 10.1371/journal.pone.0270428 [published Online First: 20220706]

166. Tse AC, Lee PH, Zhang J, et al. Effects of exercise on sleep, melatonin level, and behavioral functioning in children with autism. *Autism* 2022;26(7):1712–22. doi: 10.1177/13623613211062952 [published Online First: 20220127]

167. Hashemi A, Khodaverdi Z, Zamani MH. Effect of Wii Fit training on visual perception and executive function in boys with developmental coordination disorders: A randomized controlled trial. *Res Dev Disabil* 2022;124:104196. doi: 10.1016/j.ridd.2022.104196 [published Online First: 20220214]

168. KÖSe B, TemİZkan E, Kara Kaya Ö, et al. How does visual praxis based occupational therapy program effect motor skills in children with hyperactivity and attention disorder? Single blind randomized study design. *Journal of Experimental &amp; Clinical Medicine / Deneysel ve Klinik Tip Dergisi* 2022;39(3):803–08. doi: 10.52142/omujecm.39.3.40

169. Ji C, Yang J, Lin L, et al. Executive Function Improvement for Children with Autism Spectrum Disorder: A Comparative Study between Virtual Training and Physical Exercise Methods. *Children (Basel)* 2022;9(4) doi: 10.3390/children9040507 [published Online First: 20220403]

170. Perić DB, Milićević-Marinković B, Djurović D. The effect of the adapted soccer programme on motor learning and psychosocial behaviour in adolescents with Down syndrome. *J Intellect Disabil Res* 2022;66(6):533–44. doi: 10.1111/jir.12881 [published Online First: 20210909]

171. Nekar DM, Lee DY, Hong JH, et al. Effects of Augmented Reality Game-Based Cognitive-Motor Training on Restricted and Repetitive Behaviors and Executive Function in Patients with Autism Spectrum Disorder. *Healthcare (Basel)* 2022;10(10) doi: 10.3390/healthcare10101981 [published Online First: 20221009]

172. Yarımkaya E, Esentürk OK, İlhan EL, et al. A WhatsApp-delivered intervention to promote physical activity in young children with autism spectrum disorder. *Int J Dev Disabil* 2022;68(5):732–43. doi: 10.1080/20473869.2021.1887436 [published Online First: 20210218]

173. Arslan E, Ince G, Akyüz M. Effects of a 12-week structured circuit exercise program on physical fitness levels of children with autism spectrum condition and typically developing children. *Int J Dev Disabil* 2022;68(4):500–10. doi: 10.1080/20473869.2020.1819943 [published Online First: 20200917]

174. Balayi E, Sedaghati P, Ahmadabadi S. Effects of neuromuscular training on postural control of children with intellectual disability and developmental coordination disorders : Neuromuscular training and postural control. *BMC Musculoskelet Disord* 2022;23(1):631. doi: 10.1186/s12891-022-05569-2 [published Online First: 20220702]

175. Marzouki H, Soussi B, Selmi O, et al. Effects of Aquatic Training in Children with Autism Spectrum Disorder. *Biology (Basel)* 2022;11(5) doi: 10.3390/biology11050657 [published Online First: 20220425]

176. Ren H, Ren G, Zhan Y, et al. Examining the efficacy of dance movement and music mixed treatment on social communication impairment in children with autism - Based on family parent-child situation. *Front Psychol* 2022;13:937564. doi: 10.3389/fpsyg.2022.937564 [published Online First: 20220808]

177. Gehricke JG, Lowery LA, Alejo SD, et al. The effects of a physical exercise program, LEGO<SUP>R</SUP> and Minecraft activities on anxiety in underserved children with autism spectrum disorder. *Research in Autism Spectrum Disorders* 2022;97 doi: 10.1016/j.rasd.2022.102005

178. Downs J, Blackmore AM, Wong K, et al. Can telehealth increase physical activity in individuals with Rett syndrome? A multicentre randomized controlled trial. *Dev Med Child Neurol* 2023;65(4):489–97. doi: 10.1111/dmcn.15436 [published Online First: 20221025]

179. Fritz K, O'Connor PJ. Effects of a 6 Week Yoga Intervention on Executive Functioning in Women Screening Positive for Adult ADHD: A Pilot Study. *Front Sports Act Living* 2022;4:746409. doi: 10.3389/fspor.2022.746409 [published Online First: 20220224]

180. Teixeira-Machado L, Arida RM, Ziebold C, et al. A pilot randomized controlled clinical trial of dance practice for functionality in autistic children and adolescent with all levels of need support. *Complement Ther Clin Pract* 2022;49:101650. doi: 10.1016/j.ctcp.2022.101650 [published Online First: 20220828]

181. Bibro MA, Wódka K, Smoła E, et al. The influence of 15‐weeks climbing program on the static and dynamic balance of young adults with mild and moderate intellectual disabilities. *Journal of Applied Research in Intellectual Disabilities* 2023;36(3):529–37. doi: 10.1111/jar.13075

182. Savage MN, Tomaszewski BT, Hume KA. Step It Up: Increasing Physical Activity for Adults With Autism Spectrum Disorder and Intellectual Disability Using Supported Self-Management and Fitbit Technology. *Focus on Autism and Other Developmental Disabilities* 2022;37(3):146–57. doi: 10.1177/10883576211073700

183. Zhao M, You Y, Li J, et al. The Effects of Therapeutic Horseback Riding Program on Motor Skills in Children with Autism Spectrum Disorder. *International Journal of Mental Health Promotion* 2022;24(4):475–89. doi: 10.32604/ijmhp.2022.021361

184. Elshafey MA, Alsakhawi RS. Inspiratory Muscle Training and Physical Fitness in Children With Down Syndrome: A Randomized Controlled Trial. *Arch Phys Med Rehabil* 2022;103(12):2279–87. doi: 10.1016/j.apmr.2022.04.005 [published Online First: 20220506]

185. Saidmamatov O, Sanjarbek K, Vasconcelos O, et al. Sustainability of a Given Ten-Week Motor Skills Training Program for Children with Developmental Coordination Disorder. *Sports (Basel)* 2022;10(11) doi: 10.3390/sports10110164 [published Online First: 20221024]

186. Saidmamatov O, Rodrigues P, Vasconcelos O. Motor Skills Training Program Reinforces Crossing the Body's Midline in Children with Developmental Coordination Disorder. *Symmetry-Basel* 2022;14(6) doi: 10.3390/sym14061259

187. Vreuls RJA, Mockenhaupt J, Tillmann V, et al. Effect of Indoor Climbing on Occupational Self-Efficacy and Employability: Results of a 10-Month Randomised Controlled Study of Persons with Intellectual Disability. *Int J Environ Res Public Health* 2022;19(20) doi: 10.3390/ijerph192013399 [published Online First: 20221017]

188. Bahiraei S, Daneshmandi H. The effect and durability of functional exercises on Balance Evaluation Systems Test (BESTest) scores in people with intellectual disabilities: a preliminary report. *J Intellect Disabil Res* 2022;66(11):880–92. doi: 10.1111/jir.12978 [published Online First: 20220906]

189. Kouhbanani SS, Zarenezhad S, Arabi SM. Mind-body exercise affects attention switching and sustained attention in female adults with Attention Deficit/Hyperactivity Disorder: A randomized, controlled trial with 6-month follow-up. *Current Psychology* 2023;42(24):20983–94. doi: 10.1007/s12144-022-03216-6

190. Ludyga S, Mücke M, Leuenberger R, et al. Behavioral and neurocognitive effects of judo training on working memory capacity in children with ADHD: A randomized controlled trial. *Neuroimage Clin* 2022;36:103156. doi: 10.1016/j.nicl.2022.103156 [published Online First: 20220817]

191. Park SB, Ju Y, Kwon H, et al. Effect of a Cognitive Function and Social Skills-Based Digital Exercise Therapy Using IoT on Motor Coordination in Children with Intellectual and Developmental Disability. *Int J Environ Res Public Health* 2022;19(24) doi: 10.3390/ijerph192416499 [published Online First: 20221208]

192. Chang SH, Shie JJ, Yu NY. Enhancing Executive Functions and Handwriting with a Concentrative Coordination Exercise in Children with ADHD: A Randomized Clinical Trial. *Percept Mot Skills* 2022;129(4):1014–35. doi: 10.1177/00315125221098324 [published Online First: 20220504]

193. Wang S, Yu H, Lu Z, et al. Eight-week virtual reality training improves lower extremity muscle strength but not balance in adolescents with intellectual disability: A randomized controlled trial. *Front Physiol* 2022;13:1053065. doi: 10.3389/fphys.2022.1053065 [published Online First: 20221122]

194. Shanker S, Pradhan B. Effect of Yoga on the Motor Proficiency of Children with Autism Spectrum Disorder and the Feasibility of its Inclusion in Special School Environments. *Adapt Phys Activ Q* 2022;39(2):247–67. doi: 10.1123/apaq.2021-0108 [published Online First: 20220119]

195. Yu S, Gao Y, Wang A, et al. Effectiveness of an adapted physical activity intervention for weight management in adolescents with intellectual disability: A randomized controlled trial. *Pediatr Obes* 2022;17(5):e12882. doi: 10.1111/ijpo.12882 [published Online First: 20220110]

196. Hattabi S, Forte P, Kukic F, et al. A Randomized Trial of a Swimming-Based Alternative Treatment for Children with Attention Deficit Hyperactivity Disorder. *Int J Environ Res Public Health* 2022;19(23) doi: 10.3390/ijerph192316238 [published Online First: 20221204]

197. Adiyaman S, Ozkan Z. Investigating the Effects of 12-Week Fun Athletics Program on Motor Skill Development of 10-14 Year old Children with Mild Intellectual Disability. *Journal for Educators Teachers and Trainers* 2022;13(6):708–19. doi: 10.47750/jett.2022.13.06.070

198. Cai W, Baek SS. Effects of 24‐week basketball programme on body composition and functional fitness on adults with Down syndrome. *Journal of Intellectual Disability Research* 2022;66(12):939–51. doi: 10.1111/jir.12951

199. Liang X, Qiu H, Wang P, et al. The impacts of a combined exercise on executive function in children with ADHD: A randomized controlled trial. *Scand J Med Sci Sports* 2022;32(8):1297–312. doi: 10.1111/sms.14192 [published Online First: 20220605]

200. Sun Y, Yu S, Wang A, et al. Effectiveness of an adapted physical activity intervention on health-related physical fitness in adolescents with intellectual disability: a randomized controlled trial. *Sci Rep* 2022;12(1):22583. doi: 10.1038/s41598-022-26024-1 [published Online First: 20221230]

201. KurtoĞLu A, ÇAr B, Konar N, et al. Investigation of the Effect of Eight-Week Aqua Fitness Exercises on Some Strength Parameters and Body Composition with Autism Spectrum Disorder. *International Journal of Disabilities Sports &amp; Health Sciences* 2023;6(3):279–86. doi: 10.33438/ijdshs.1254879

202. Broupi AE, Kokaridas D, Tsimaras V, et al. The effect of a visual arts and exercise program on communication and social skills of students with autism spectrum disorders. *Advances in Autism* 2023;9(4):388–401. doi: 10.1108/aia-05-2022-0031

203. Kashi A, Dawes H, Mansoubi M, et al. The Effect of a Physical Exercise Package on the Motor Proficiency of Students with Down Syndrome. *Iranian Journal of Pediatrics* 2023;33(1)

204. Haghighi AH, Broughani S, Askari R, et al. Combined Physical Training Strategies Improve Physical Fitness, Behavior, and Social Skills of Autistic Children. *J Autism Dev Disord* 2023;53(11):4271–79. doi: 10.1007/s10803-022-05731-8 [published Online First: 20220909]

205. Gutiérrez-Cruz C, Roman-Espinaco A, Muñoz-López S, et al. Effect of a resistance training programme implemented with high levels of effort on physical fitness in people with intellectual disabilities living in group homes: a randomised controlled trial. *J Intellect Disabil Res* 2023;67(8):770–81. doi: 10.1111/jir.13044 [published Online First: 20230522]

206. Farah Izzazaya Mohd F, Nurul Azuar H, Nur Syamsina A, et al. Effects of 6-week Circuit Training on Anaerobic Performance and Simple Reaction Time in Individuals with Intellectual Disability (ID) in Kelantan State. *Malaysian Journal of Health Sciences / Jurnal Sains Kesihatan Malaysia* 2023;21(1):95–104. doi: 10.17576/JSKM-2023-2101-09

207. Shafaei H, Sarsahra HA, Rezaei N. Effectiveness of Yoga Exercises, Drug Therapy, and Combined Therapy in Reducing Symptoms of Selective Attention Deficit and Impulsivity among Children with Attention Deficit/Hyperactivity Disorder. *Journal of Sport Psychology (2676-3729)* 2023;15(1):226–37. doi: 10.48308/mbsp.2021.104777.2317

208. Allam HH, Muhsen A, Alshehri FH, et al. The effect of different exercise intensities on knee and ankle joints' torque and functional mobility in children with Down Syndrome. *Sport Tk-Revista Euroamericana De Ciencias Del Deporte* 2023;12

209. Liu HLV, Sun F, Tse CYA. Examining the Impact of Physical Activity on Sleep Quality in Children With ADHD. *J Atten Disord* 2023;27(10):1099–106. doi: 10.1177/10870547231171723 [published Online First: 20230529]

210. Ji H, Wu S, Won J, et al. The Effects of Exergaming on Attention in Children With Attention Deficit/Hyperactivity Disorder: Randomized Controlled Trial. *JMIR Serious Games* 2023;11:e40438. doi: 10.2196/40438 [published Online First: 20230509]

211. Ben Hassen I, Abid R, Ben Waer F, et al. Intervention Based on Psychomotor Rehabilitation in Children with Autism Spectrum Disorder ASD: Effect on Postural Control and Sensory Integration. *Children (Basel)* 2023;10(9) doi: 10.3390/children10091480 [published Online First: 20230830]

212. Kashi A, Dawes H, Mansoubi M, et al. The Effect of an Exercise Package for Students with Intellectual Disability on Motor and Social Development. *Iran J Child Neurol* 2023;17(2):93–110. doi: 10.22037/ijcn.v17i1.36644 [published Online First: 20230315]

213. Svedell LA, Holmqvist KL, Lindvall MA, et al. Feasibility and tolerability of moderate intensity regular physical exercise as treatment for core symptoms of attention deficit hyperactivity disorder: a randomized pilot study. *Front Sports Act Living* 2023;5:1133256. doi: 10.3389/fspor.2023.1133256 [published Online First: 20230515]

214. Dinu LM, Singh SN, Baker NS, et al. The Effects of Different Exercise Approaches on Attention Deficit Hyperactivity Disorder in Adults: A Randomised Controlled Trial. *Behav Sci (Basel)* 2023;13(2) doi: 10.3390/bs13020129 [published Online First: 20230202]

215. Prieto LA, Meera B, Barry A, et al. A randomized parent-mediated physical activity intervention for autistic children. *Autism Res* 2023;16(7):1450–61. doi: 10.1002/aur.2969 [published Online First: 20230626]

216. Dehghani M, Jafarnezhadgero AA, Darvishani MA, et al. Effects of an 8-week multimodal exercise program on ground reaction forces and plantar pressure during walking in boys with autism spectrum disorder. *Trials* 2023;24(1):170. doi: 10.1186/s13063-023-07158-7 [published Online First: 20230308]

217. Büyükçelik NM, Yiğit S, Turhan B. An investigation of the effects of dual-task balance exercises on balance, functional status and dual-task performance in children with Down syndrome. *Dev Neurorehabil* 2023;26(5):320–27. doi: 10.1080/17518423.2023.2233031 [published Online First: 20230704]

218. Hosseini SH, Azimizadeh MJ. The Effect of an Exercise Protocol Focused on the Combination of Strength and Proprioceptive Exercises on the Balance and Motor Performance in Boys with Intellectual Disability. *Journal of Advanced Sport Technology* 2023;7(3):1–11.

219. Shanker S, Pradhan B. Effect of yoga on the social responsiveness and problem behaviors of children with ASD in special schools: A randomized controlled trial. *Explore (NY)* 2023;19(4):594–99. doi: 10.1016/j.explore.2022.12.004 [published Online First: 20221225]

220. Lin YY, Su CT, Liao YH, et al. Effects of rope skipping exercise on physical, cardiovascular fitness and exercise tolerance in adolescent students with moderate intellectual disability. *J Intellect Disabil Res* 2023;67(11):1136–49. doi: 10.1111/jir.13071 [published Online First: 20230814]

221. Wang Y, Wu X, Chen H. Badminton Improves Executive Function in Adults Living with Mild Intellectual Disability. *Int J Environ Res Public Health* 2023;20(4) doi: 10.3390/ijerph20043673 [published Online First: 20230219]

222. Jin YR, Sung YS, Koh CL, et al. Efficacy of Motor Interventions on Functional Performance Among Preschool Children With Autism Spectrum Disorder: A Pilot Randomized Controlled Trial. *Am J Occup Ther* 2023;77(6) doi: 10.5014/ajot.2023.050283

223. Veljkovic AA, Stojanovic M, Stojiljkovic N. The effects of a manipulative skills intervention programme on the motor proficiency of adolescents with intellectual disabilities. *South African Journal of Education* 2024;44(1) doi: 10.15700/saje.v44n1a2273

224. Cherni A, Ouerghi N, Jebabli N, et al. A Tai Chi Program Improves Physical Performance Measures in Adolescents With Down Syndrome and Enhances Their Parents' Psychological Well-Being: A Randomized Controlled Trial. *Adapt Phys Activ Q* 2025;42(2):228–46. doi: 10.1123/apaq.2024-0035 [published Online First: 20250212]

225. Al-Nemr A, Reffat S. Effect of Pilates exercises on balance and gross motor coordination in children with Down syndrome. *Acta Neurol Belg* 2024;124(5):1499–505. doi: 10.1007/s13760-024-02517-w [published Online First: 20240401]

226. Mero Piedra AL, Pesthy O, Marton K. Effects of a physical education intervention on attention and inhibitory control in Ecuadorian children with intellectual disabilities. *J Intellect Disabil* 2024;28(1):261–74. doi: 10.1177/17446295231189018 [published Online First: 20230717]

227. Kilany A, Elhadidy ME, Ganem MMF, et al. Diet and physical activity modification: impact on adaptive behavior and biochemical measures of children with Down syndrome. *Int J Dev Disabil* 2024;70(6):1082–91. doi: 10.1080/20473869.2024.2383807 [published Online First: 20240724]

228. Suarez-Villadat B, Corredeira RM, Vega ML, et al. Strength versus aerobic program: effects on body composition and health-related physical fitness levels of youths with Down syndrome. *Int J Dev Disabil* 2024;70(5):943–56. doi: 10.1080/20473869.2022.2162627 [published Online First: 20230103]

229. Suarez-Villadat B, Sadarangani KP, Villagra A. Effectiveness of exergames programme to modify body composition and health-related physical fitness in adolescents with down syndrome after COVID-19 quarantine. *Eur J Sport Sci* 2023;23(11):2210–20. doi: 10.1080/17461391.2023.2214805 [published Online First: 20230528]

230. Vieira LourenÇO CC, Burhaein E, Phytanza DTP, et al. Is There an Effect of the Trampoline Program for Autism Spectrum Disorder Children in Portugal? *International Journal of Disabilities Sports &amp; Health Sciences* 2024;7(2):458–68. doi: 10.33438/ijdshs.1342102

231. Tse ACY, Lee PH, Sit CHP, et al. Comparing the Effectiveness of Physical Exercise Intervention and Melatonin Supplement in Improving Sleep Quality in Children with ASD. *J Autism Dev Disord* 2024;54(12):4456–64. doi: 10.1007/s10803-023-06172-7 [published Online First: 20231111]

232. Zhou D, Liu Z, Gong G, et al. Decreased Functional and Structural Connectivity is Associated with Core Symptom Improvement in Children with Autism Spectrum Disorder After Mini-basketball Training Program. *J Autism Dev Disord* 2024;54(12):4515–28. doi: 10.1007/s10803-023-06160-x [published Online First: 20231026]

233. Steyn E, Boer PH, Joubert G. The effect of a walking, dancing, and strength training program on the functional fitness of adults with intellectual disability: A randomised controlled trial. *J Intellect Dev Disabil* 2024;49(2):186–98. doi: 10.3109/13668250.2023.2260582 [published Online First: 20231222]

234. Tascioglu EN, Karademir S, Kara K, et al. Effectiveness of Power Exercises Compared to Traditional Strength Exercises on Motor Skills, Muscle Performance and Functional Muscle Strength of Children with Attention Deficit Hyperactivity Disorder: A Single-Blind Randomized Controlled Trial. *Dev Neurorehabil* 2024;27(1-2):17–26. doi: 10.1080/17518423.2024.2345335 [published Online First: 20240422]

235. Top E, Akil M. Effect of theraband exercises on muscle strength and motor development of individuals with moderate intellectual disability. *Somatosens Mot Res* 2024;41(4):197–204. doi: 10.1080/08990220.2023.2194386 [published Online First: 20230331]

236. Punar E, Şevgin Ö. Effect of goal-directed perceptual-motor exercise on children with specific learning difficulties: a randomized controlled trial. *BMC Pediatr* 2024;24(1):820. doi: 10.1186/s12887-024-05309-6 [published Online First: 20241219]

237. Yarımkaya E, Esentürk OK, İlhan EL, et al. Zoom-delivered Physical Activities Can Increase Perceived Physical Activity Level in Children with Autism Spectrum Disorder: a Pilot Study. *J Dev Phys Disabil* 2023;35(2):189–207. doi: 10.1007/s10882-022-09854-9 [published Online First: 20220603]

238. Adibsaber F, Ansari S, Elmieh A, et al. Vitamin D3 Supplementation and Aquatic Exercise Combination as a Safe- Efficient Therapeutic Strategy to Ameliorate Interleukin-6 and 10, and Social Interaction in Children with Autism. *Iran J Child Neurol* 2024;18(3):91–102. doi: 10.22037/ijcn.v18i3.43021 [published Online First: 20240622]

239. Sun F, Fang Y, Ho YF, et al. Effectiveness of a game-based high-intensity interval training on executive function and other health indicators of children with ADHD: A three-arm partially-blinded randomized controlled trial. *J Exerc Sci Fit* 2024;22(4):408–16. doi: 10.1016/j.jesf.2024.09.001 [published Online First: 20240905]

240. Jouira G, Rebai H, Alexe DI, et al. Effect of Combined Training With Balance, Strength, and Plyometrics on Physical Performance in Male Sprint Athletes With Intellectual Disabilities. *Adapt Phys Activ Q* 2024;41(3):382–401. doi: 10.1123/apaq.2023-0105 [published Online First: 20240228]

241. Zourmand G, Pavlovic R, Taheri M. The Effect of School Games on Motor Skills Development in Children with Autism. *Annals of Applied Sport Science* 2024;12 doi: 10.61186/aassjournal.1294

242. Walters GW, Taylor S, Sweeney EL, et al. Effect of an acute bout of exercise on executive function and sleep in children with attention deficit hyperactivity disorder and autism spectrum disorder. *Mental Health and Physical Activity* 2024;27 doi: 10.1016/j.mhpa.2024.100624

243. Duan G, Wang Y, Hou H, et al. Effect of cognitive-behavioral therapy-based physical play intervention on empathic ability of children with intellectual disabilities. *Sci Rep* 2024;14(1):30171. doi: 10.1038/s41598-024-80066-1 [published Online First: 20241204]

244. Kalyoncu G, Odek U. Effects of 8-week hemsball training on resting heart rate, body mass index, sleep time and sleep efficiency in women with mild intellectual development disorder. *International Journal of Developmental Disabilities* 2024 doi: 10.1080/20473869.2024.2340179

245. Zolghadr H, Sedaghati P, Daneshmandi H, et al. Effects of mini-basketball training on improving the motor coordination of children with intellectual disabilities and developmental coordination disorders: a randomizedcontrolled trial. *BMC Sports Sci Med Rehabil* 2024;16(1):250. doi: 10.1186/s13102-024-01044-8 [published Online First: 20241223]

246. Chen H, Liang Q, Wang B, et al. Sports game intervention aids executive function enhancement in children with autism - An fNIRS study. *Neurosci Lett* 2024;822:137647. doi: 10.1016/j.neulet.2024.137647 [published Online First: 20240117]

247. Lee K. Enhancing Motor Performance and Physical Fitness in Children with Developmental Coordination Disorder Through Fundamental Motor Skills Exercise. *Healthcare (Basel)* 2024;12(21) doi: 10.3390/healthcare12212142 [published Online First: 20241028]

248. Jovicevic L, Tusak M, Majeric M. EFFECTS OF AN EXERCISE AND MINDFULNESS PROGRAMME ON SYMPTOMS OF ATTENTION DEFICIT HYPERACTIVITY DISORDER IN BOYS AGED 9-11 YEARS. *Kinesiologia Slovenica* 2024;30(2):39–55. doi: 10.52165/kinsi.30.2.39-55

249. Shuoqi LI, Rong G, Chunjing L, et al. Effects of moderate-intensity aerobic exercise combined with acupuncture on attention function of mentally-retarded adolescents: a randomised controlled trial. *J Tradit Chin Med* 2023;43(1):154–59. doi: 10.19852/j.cnki.jtcm.20220425.003

250. Zhao L, Agazzi H, Du Y, et al. A Digital Cognitive-Physical Intervention for Attention-Deficit/Hyperactivity Disorder: Randomized Controlled Trial. *J Med Internet Res* 2024;26:e55569. doi: 10.2196/55569 [published Online First: 20240510]

251. Ge LK, Man X, Cai K, et al. Sharing Our World: Impact of Group Motor Skill Learning on Joint Attention in Children with Autism Spectrum Disorder. *J Autism Dev Disord* 2024 doi: 10.1007/s10803-024-06528-7 [published Online First: 20240904]

252. Aliabad MK, Razavi MN, Sadeghi M, et al. The Effects of Physical Activity on Key Variables That Influence the Characteristics of Children with Attention Deficit Hyperactivity Disorder. *International Journal of Sport Studies for Health* 2024;7(4):95–105. doi: 10.61838/kman.intjssh.7.4.10

253. Siddiqui M, Farooqui S, Rizvi J, et al. Effects of Simulated Equestrian Therapy in Improving Motor Proficiency among Down Syndrome Children - A Randomized Controlled Trial. *Int J Exerc Sci* 2024;17(1):1193–207. doi: 10.70252/lral2790 [published Online First: 20240801]

254. Belaiba M, Laatar R, Borji R, et al. Time Limited Benefits of Physical and Proprioceptive Training on Physical Fitness Components in Children With Autism Spectrum Disorders. *Percept Mot Skills* 2024;131(3):785–804. doi: 10.1177/00315125241244484 [published Online First: 20240402]

255. Salleg-Cabarcas MJ, Robledo-Castro C, Monsalve-Vertel CP. Effect of Hit-Sport-Game Exercise Training on Self-Control and Regulation in Children With Attention Deficit Hyperactivity Disorder (ADHD). *Glob Pediatr Health* 2024;11:2333794x241287095. doi: 10.1177/2333794x241287095 [published Online First: 20240928]

256. Strucelj M, Horvat I, Bobic TT. COMBINED EXERCISE TRAINING PROGRAMME LEADS TO DIFFERENT EFFECTS ON POWER, MUSCULAR ENDURANCE AND BALANCE OF INSTITUTIONALIZED PERSONS WITH INTELLECTUAL DISABILITIES. *Kinesiology* 2024;56(2) doi: 10.26582/k.56.2.7

257. Montazeri H, Ayatizadeh Tafti F, Samadi H, et al. Effects of Selected Cognitive-Motor Intervention on the Level of Physical Literacy and Executive Functions in Attention deficit/hyperactivity disorder Girls: A One-month Follow-up Study. *Cuadernos de Psicología del Deporte* 2024;24(1):80–94. doi: 10.6018/cpd.574091

258. Adeeb N, Farooqui SI, Meher Hasan Z, et al. Foot Muscle Exercise: A Novel Approach to Improve Motor Functions in Children with Down Syndrome Having Pes Planus - A Randomized Controlled Trial. *Dev Neurorehabil* 2024;27(3-4):145–53. doi: 10.1080/17518423.2024.2365798 [published Online First: 20240618]

259. Beyaz O, Akpinar S, Demirhan G. Effect of 12-week traditional archery on motor performances and quality of life of adolescents with mild intellectual disability. *International Journal of Developmental Disabilities* 2024 doi: 10.1080/20473869.2024.2436476

260. Zhao P, Chen K, Zhu G, et al. Effects of aquatic exercise intervention on executive function and brain-derived neurotrophic factor of children with autism spectrum disorder. *Res Dev Disabil* 2024;150:104759. doi: 10.1016/j.ridd.2024.104759 [published Online First: 20240524]

261. Castaño PRL, Suárez DPM, González ER, et al. Effects of Physical Exercise on Gross Motor Skills in Children with Autism Spectrum Disorder. *J Autism Dev Disord* 2024;54(8):2816–25. doi: 10.1007/s10803-023-06031-5

262. Cheong PL, Wu YT, Xie LN, et al. Effects of Rhythmic Motor and Behavioral Intervention on Motor and Social Performance in Preschool Children with Autism Spectrum Disorder: A Pilot Randomized Controlled Study. *J Autism Dev Disord* 2025 doi: 10.1007/s10803-025-06922-9 [published Online First: 20250611]

263. Rao PT, Solomon JM. Training Response Abilities of Children With Intellectual Disabilities: A Randomized Controlled Trial. *J Mot Behav* 2024;56(4):439–52. doi: 10.1080/00222895.2024.2322555 [published Online First: 20240307]

264. Mohamed RI, El-Hadidy EI, Dawoud M, et al. Effect of Group Exercise Program on Social Skills and Bone Mineral Density in Children with Autism. *Egyptian Journal of Hospital Medicine* 2024;96:2665–71. doi: 10.21608/ejhm.2024.369498

265. Arsham S, Mirzaei M, Domingos C. Bike-Riding Training may Improve Communication Skills and Stereotyped Behavior in Adolescents With Autism. *J Autism Dev Disord* 2025 doi: 10.1007/s10803-024-06694-8 [published Online First: 20250108]

266. Sabri S, Zhang MY, Guo L, et al. Effectiveness of school-based physical activity programs in enhancing attention, academic performance, and social relationships among children with intellectual disabilities: evidence from Pakistani schools. *Front Psychol* 2024;15:1431890. doi: 10.3389/fpsyg.2024.1431890 [published Online First: 20240910]

267. Ludyga S, Bruggisser F, Leuenberger R, et al. Acute effects of exercise on gaze fixation and affective response inhibition in children with autism spectrum disorder: A randomized cross-over study. *Autism Res* 2024;17(9):1934–43. doi: 10.1002/aur.3224 [published Online First: 20240830]

268. Liu C, Wong SH, Arbour-Nicitopoulos K, et al. Effects of physical activity on suicidal ideation and sleep disturbances in adolescents with attention-deficit hyperactivity disorder: A randomized controlled trial. *J Affect Disord* 2026;393(Pt B):120369. doi: 10.1016/j.jad.2025.120369 [published Online First: 20251009]

269. Liang X, Xu RH, Zhao M, et al. Impact of a physical exercise intervention on psychological resilience and mental ill-being in medical-naïve children with attention-deficit/hyperactivity disorder: a pilot randomised controlled trial. *Psychol Health Med* 2024;29(10):1764–75. doi: 10.1080/13548506.2024.2407449 [published Online First: 20241020]

270. Cui X, Wang S. Research on the Effect of Dance Therapy on Improving Social Communication Ability of Children with Autism. *International Journal of Mental Health Promotion* 2024;26(5):407–16. doi: 10.32604/ijmhp.2023.030135

271. Shen X, Huang P, Liu Q, et al. Telehealth based parental support over 6 months improves physical activity and sleep quality in children with autism: a randomized controlled trial. *Front Pediatr* 2024;12:1496827. doi: 10.3389/fped.2024.1496827 [published Online First: 20241025]

272. Shen X, Huang P, Nie B, et al. An Adapted Physical Activity Program for Adolescents with an Intellectual Disability: An International Classification of Functioning, Disability, and Health Perspective. *Life (Basel)* 2024;14(10) doi: 10.3390/life14101314 [published Online First: 20241016]

273. Ju X, Liu H, Xu J, et al. Effect of Yoga Intervention on Problem Behavior and Motor Coordination in Children with Autism. *Behav Sci (Basel)* 2024;14(2) doi: 10.3390/bs14020116 [published Online First: 20240204]

274. Zhang Z, Li R, Zhou Z, et al. The effect of physical activity on quality of life and parenting stress in children with attention-deficit/hyperactivity disorder: A randomized controlled trial. *Disabil Health J* 2023;16(1):101377. doi: 10.1016/j.dhjo.2022.101377 [published Online First: 20220915]

275. Huang Z, Li L, Lu Y, et al. Effects of rope skipping exercise on working memory and cardiorespiratory fitness in children with attention deficit hyperactivity disorder. *Front Psychiatry* 2024;15:1381403. doi: 10.3389/fpsyt.2024.1381403 [published Online First: 20240523]

276. Janbozorgi Z, Khalaji H, Moradi J. The effect of individual and paired Brailletonik exercises on balance and reaction time in children with intellectual disability. *BMC Sports Sci Med Rehabil* 2024;16(1):103. doi: 10.1186/s13102-024-00891-9 [published Online First: 20240503]

277. Dhingra A, Gupta A, Aditi. Effect of Aerobic Training on Anxiety, Activities of Daily Living and Visual Motor Skills Performance in Children with Autism: A Randomised Controlled Trial. *Journal of Clinical and Diagnostic Research* 2025;19(2):SC6–SC11. doi: 10.7860/jcdr/2025/73346.20624

278. Falivene A, Scaccabarozzi G, Busti Ceccarelli S, et al. Virtual Reality-Based Postural Balance Training in Autistic Children: A Pilot Randomized Controlled Trial. *J Clin Med* 2025;14(16) doi: 10.3390/jcm14165616 [published Online First: 20250808]

279. Sabaghi A, Ebrahimi B, Yousofvand N, et al. Comparative effects of moderate-intensity continuous training and high-intensity interval training on ADHD symptoms and behavioral inhibition in children. *Eur J Pediatr* 2025;184(2):183. doi: 10.1007/s00431-025-06022-x [published Online First: 20250207]

280. Köse B, Temizkan E, Kara Ö K, et al. Investigating the Maintained Motor Skill Achievements in a Visual Praxis Based Occupational Therapy Program: Single Blind Randomized Follow up Study. *J Autism Dev Disord* 2025;55(12):4226–33. doi: 10.1007/s10803-024-06513-0 [published Online First: 20240828]

281. İDarecİ B, Yarimkaya E. THE EFFECT OF INCLUSIVE PHYSICAL ACTIVITIES APPLIED TO CHILDREN WITH INTELLECTUAL DISABILITIES ON THEIR PHYSICAL ACTIVITY LEVEL AND SOCIAL SKILLS. *Nigde University Journal of Physical Education &amp; Sport Sciences / Nigde Üniversitesi Beden Egitimi ve Spor Bilimleri Dergisi* 2025;19(1):1–16. doi: 10.61962/bsd.1536275

282. Suarez-Villadat B, Corredeira R, Veiga M, et al. Psychosocial behavior, motor learning, and health-related physical fitness in young people with Down syndrome: development of an adapted soccer program. *International Journal of Developmental Disabilities* 2025 doi: 10.1080/20473869.2025.2462913

283. Kaplan Kılıç B, Bumin G, Öğütlü H. Effect of Telerehabilitation on Handwriting Performance in Children With Attention Deficit Hyperactivity Disorder: Randomized Controlled Trial. *Child Care Health Dev* 2025;51(2):e70055. doi: 10.1111/cch.70055

284. Liu C, Yang Y, Wong SH, et al. The effects of physical activity on mental health in adolescents with attention-deficit hyperactivity disorder: a randomized controlled trial. *Int J Behav Nutr Phys Act* 2025;22(1):47. doi: 10.1186/s12966-025-01745-4 [published Online First: 20250417]

285. He C, Huang H, Guo S, et al. The effects of combined exercise on working memory in children with attention-deficit/hyperactivity disorder: a randomized controlled trial. *J Sci Med Sport* 2025 doi: 10.1016/j.jsams.2025.09.010 [published Online First: 20250922]

286. Kanzari C, Hawani A, Ayed KB, et al. The Impact of a Music- and Movement-Based Intervention on Motor Competence, Social Engagement, and Behavior in Children with Autism Spectrum Disorder. *Children (Basel)* 2025;12(1) doi: 10.3390/children12010087 [published Online First: 20250114]

287. Pan C-Y, Tsai C-L, Chen F-C, et al. Effectiveness of a Fundamental Movement Skill Intervention in Children With Autism Spectrum Disorder: A Randomized Controlled Trial. *Focus on Autism and Other Developmental Disabilities* 2025;40(2):59–68. doi: 10.1177/10883576241311171

288. Huang CY, Chen WF, Tsai CL, et al. Effects of Inline Skating Exercise on Symptoms, Executive Functions, and Motor Proficiency in Children With ADHD: A Randomized Controlled Trial. *Occup Ther Int* 2025;2025:4254970. doi: 10.1155/oti/4254970 [published Online First: 20250508]

289. Zhu F, Kuang D, Zhu X, et al. Efficacy of a mobile health with tailored personalized exercise rehabilitation program for ADHD children: randomized controlled trial. *J Affect Disord* 2025;387:119495. doi: 10.1016/j.jad.2025.119495 [published Online First: 20250528]

290. Karakaş G, Eroğlu Kolayiş I, Bayazıt B. Effects of adapted physical activity on the motor development of children with mild intellectual disability. *Int J Dev Disabil* 2025;71(5):777–87. doi: 10.1080/20473869.2024.2332751 [published Online First: 20240411]

291. Hatipoğlu Özcan G, Özer DF, Pınar S. Effects of Motor Intervention Program on Academic Skills, Motor Skills and Social Skills in Children with Autism Spectrum Disorder. *J Autism Dev Disord* 2025;55(8):2628–42. doi: 10.1007/s10803-024-06384-5 [published Online First: 20240516]

292. Mao HY, Qi K, Meng SQ, et al. The impact of a ball sports combination training program on physical fitness and body mass Index in children with autism spectrum disorder. *Front Pediatr* 2025;13:1590666. doi: 10.3389/fped.2025.1590666 [published Online First: 20250717]

293. Babagoltabar-Samakoush H, Aminikhah B, Bahiraei S. Effectiveness of dynamic neuromuscular stabilization training on strength, endurance, and flexibility in adults with intellectual disabilities, a randomized controlled trial. *Sci Rep* 2025;15(1):768. doi: 10.1038/s41598-024-85046-z [published Online First: 20250104]

294. Babagoltabar-Samakoush H, Aminikhah B, Bahiraei S. The effects and durability of an 8-week dynamic neuromuscular stabilization program on balance and coordination in adult males with intellectual disabilities: a randomized controlled trial. *BMC Sports Sci Med Rehabil* 2025;17(1):18. doi: 10.1186/s13102-025-01062-0 [published Online First: 20250131]

295. Zolghadr H, Sedaghati P, Daneshmandi H, et al. Effects of dual-task functional training on the movement competence of individuals with intellectual disabilities and developmental coordination disorders: a randomized controlled trial. *Disabil Rehabil* 2025:1–9. doi: 10.1080/09638288.2025.2519496 [published Online First: 20250619]

296. Sepehri Bonab H, Ebrahimi Sani S, Behzadnia B. The Impact of Virtual Reality Intervention on Emotion Regulation and Executive Functions in Autistic Children. *Games Health J* 2025;14(2):146–58. doi: 10.1089/g4h.2023.0240 [published Online First: 20240807]

297. Kakejani H, Farsi A, Abdoli B, et al. The Game-Based Training Improves Fundamental Movement Skills in Children with Down Syndrome. *International Journal of Disability Development and Education* 2025;72(1):77–91. doi: 10.1080/1034912x.2024.2344007

298. Miranda JM, Browne RAV, da Silva WQA, et al. Effects of a Session of Exergames and Traditional Games on Inhibitory Control in Children With Autism Spectrum Disorder: Randomized Controlled Crossover Trial. *JMIR Serious Games* 2025;13:e65562. doi: 10.2196/65562 [published Online First: 20250305]

299. Rallis K, Konstantinidou E, Hatzitaki V, et al. Proprioceptive Training Induced Adaptations of Static Balance Control: An RCT Study in Adults With Intellectual Disability. *J Intellect Disabil Res* 2025;69(4):298–309. doi: 10.1111/jir.13212 [published Online First: 20250208]

300. Wen L, Wu Z. The impact of sensory integration based sports training on motor and social skill development in children with autism spectrum disorder. *Sci Rep* 2025;15(1):19974. doi: 10.1038/s41598-025-05393-3 [published Online First: 20250606]

301. Lin L, Li N, Zhao S. The effect of intelligent monitoring of physical exercise on executive function in children with ADHD. *Alexandria Engineering Journal* 2025;122:355–63. doi: 10.1016/j.aej.2025.02.095

302. Hashempour Alooche M, Jamebozorgi A, Akbarzadeh Baghban A, et al. Effect of physical activities on cognition and the ability to perform a dual task in children with autism spectrum disorder: a randomized clinical trial. *J Bodyw Mov Ther* 2025;43:416–23. doi: 10.1016/j.jbmt.2025.05.015 [published Online First: 20250510]

303. Ahmed MM, Al Areefy AA, Alsayegh AA. Does Neurodevelopmental Approach of Physical Therapy Have an Impact on Gross Motor and Cognitive Function of Non-obese Children with Autism Spectrum Disorder (ASD)? A Randomized Controlled Trial. *Journal of Disability Research* 2025;4(1):1–11. doi: 10.57197/JDR-2024-0120

304. Firouzjah MH, Pourazar M, Nazari Kakvandi S, et al. The impact of physical activities on physical literacy and cognitive performance of children with autism spectrum disorder (ASD). *Early Hum Dev* 2025;210:106350. doi: 10.1016/j.earlhumdev.2025.106350 [published Online First: 20250731]

305. Fegh N, Khanmohammadi R, Mohammadzadeh H. The impact of perceptual-motor exercises on motor performance, social adaptation, and social self-efficacy in female students with mild intellectual disabilities: a randomized clinical trial. *Sport Sciences for Health* 2025 doi: 10.1007/s11332-025-01487-7

306. Ozsengezer N, Top E. Effects of life kinetic exercises on motor skills and cognitive health in individuals with mild intellectual disabilities: a controlled study. *International Journal of Developmental Disabilities* 2025 doi: 10.1080/20473869.2025.2514631

307. Wang Q, Jia S, Ding F, et al. Effect of fundamental movement skills practice on executive function and social interaction ability in children with moderate autism: a randomized controlled trial. *BMC Complement Med Ther* 2025;25(1):354. doi: 10.1186/s12906-025-05107-2 [published Online First: 20251002]

308. Wu QF, Cai WM. The effects of an integrated sports and arts intervention on response joint attention (RJA) eye-movement characteristics in children with mild autism. *Sci Rep* 2025;15(1):31925. doi: 10.1038/s41598-025-16970-x [published Online First: 20250829]

309. Zade RJ, Qureshi MI, Raghumahanti R, et al. Efficacy of Leap Motion Device on Fine Motor Function and Handwriting in Children With Developmental Coordination Disorder: A Randomized Control Trial. *Cureus* 2025;17(1):e78029. doi: 10.7759/cureus.78029 [published Online First: 20250126]

310. Rostami S, Fayazmilani R, Yarani E. Effectiveness of yoga training on the health-related physical fitness and balance in children with autism spectrum disorder. *Sport Sciences for Health* 2025;21(3):1653–61. doi: 10.1007/s11332-025-01391-0

311. Leung S-LA, Payne H. The Effects of a Dance/Movement Therapy Emotional Self-Regulation Program for Adolescents with Intellectual Disabilities in Hong Kong. *American Journal of Dance Therapy* 2025 doi: 10.1007/s10465-025-09423-5

312. Su WC, Srinivasan S, Bhat AN. Effects of Movement and Sedentary Play interventions on executive functioning and their relationships with sensory, repetitive, and negative behaviors of children with ASD – a pilot RCT. *Disability &amp; Rehabilitation* 2025;47(19):4999–5007. doi: 10.1080/09638288.2025.2465600

313. Su WC, Srinivasan S, Bhat AN. Effects of Creative Movement, General Movement, or Seated Play Interventions on Motor Performance in Children with Autism Spectrum Disorder: A Pilot Randomized Controlled Trial. *Res Autism Spectr Disord* 2025;119 doi: 10.1016/j.rasd.2024.102501 [published Online First: 20241030]

314. Zhang W, Cai K, Xiong X, et al. Alterations of triple network dynamic connectivity and repetitive behaviors after mini-basketball training program in children with autism spectrum disorder. *Sci Rep* 2025;15(1):2629. doi: 10.1038/s41598-025-87248-5 [published Online First: 20250121]

315. Feng XW, Hadizadeh M, Cheong JPG. Effects of Family-Professional Partnerships in Adapted Physical Education on the Fundamental Motor Skills, Adaptive Behaviors, and Physical Activity Levels of Children with Autism Spectrum Disorder and on Parent Satisfaction. *J Autism Dev Disord* 2025;55(5):1697–712. doi: 10.1007/s10803-024-06342-1 [published Online First: 20240412]

316. Wu X, Liang J, Dong Y, et al. Effects of VR-Based Serious Games on Gross Motor Skills in Chinese Children with Autism Spectrum Disorder in Special Education: A Pilot Study. *J Autism Dev Disord* 2025 doi: 10.1007/s10803-025-06810-2 [published Online First: 20250403]

317. Fu X, Shi P. The intervention effect of structured martial arts games on behavioral impairments and motor functions in children with autism spectrum disorder. *Front Psychol* 2025;16:1660040. doi: 10.3389/fpsyg.2025.1660040 [published Online First: 20250926]

318. Li Y, He YC, Wang Y, et al. Effects of Qigong vs. routine physical exercise in school-aged children with attention-deficit hyperactivity disorder: a randomized controlled trial. *World J Pediatr* 2025;21(7):720–30. doi: 10.1007/s12519-025-00890-x [published Online First: 20250310]

319. Zhang Y, Tian H, Tao Y, et al. A study on the effects of three game intervention programs on executive functions of preschool autistic children. *Int J Dev Disabil* 2025;71(1):168–78. doi: 10.1080/20473869.2023.2215606 [published Online First: 20230608]

320. Wang Y, Zhou D, Liu C, et al. Physical-intellectual badminton teaching intervention for children with intellectual disabilities. *Front Psychol* 2025;16:1445620. doi: 10.3389/fpsyg.2025.1445620 [published Online First: 20250311]

321. Deng C, Zhu Z, Luo G, et al. Effects of gymnastics exercises on executive function in children with autism spectrum disorder aged 6 to 9 years: a pilot study of a randomized controlled trial. *Front Psychol* 2025;16:1660305. doi: 10.3389/fpsyg.2025.1660305 [published Online First: 20251027]

322. Shen X, Huang P, Su M, et al. Impact of remote collaboration-based family physical activity on activity levels and quality of life in children with intellectual developmental disabilities. *Front Public Health* 2024;12:1464099. doi: 10.3389/fpubh.2024.1464099 [published Online First: 20241120]

323. Noetel M, Sanders T, Tracey D, et al. Effects of a school-based physical activity intervention on children with intellectual disability: a cluster randomized trial. *Int J Behav Nutr Phys Act* 2025;22(1):103. doi: 10.1186/s12966-025-01798-5 [published Online First: 20250725]
